# Urea cycle modulation by combined SGLT2 inhibitors and metformin

**DOI:** 10.1186/s12916-025-04609-7

**Published:** 2026-01-08

**Authors:** Makoto Harada, Jonathan Adam, Siyu Han, Mengya Shi, Jianhong Ge, Jutta Lintelmann, Alexander Cecil, Sven Zukunft, Cornelia Prehn, Michael Witting, Markus F. Scheerer, Susanne Neschen, Martin Irmler, Johannes Beckers, Jerzy Adamski, Daniel Teupser, Birgit Linkohr, Christian Gieger, Martin Hrabě de Angelis, Annette Peters, Rui Wang-Sattler

**Affiliations:** 1 Institute of Translational Genomics, Helmholtz Zentrum München, German Research Center for Environmental Health, Neuherberg, Germany; 2 German Center for Diabetes Research (DZD), München-Neuherberg, Germany; 3 Institute of Epidemiology, Helmholtz Zentrum München, German Research Center for Environmental Health, Neuherberg, Germany; 4 Research Unit of Molecular Epidemiology, Institute of Epidemiology, Helmholtz Zentrum München, German Research Center for Environmental Health, Neuherberg, Germany; 5 TUM School of Medicine and Health, Technical University of Munich, Munich, Germany; 6 Metabolomics and Proteomics Core, Helmholtz Zentrum München, German Research Center for Environmental Health, Neuherberg, Germany; 7 Chair of Analytical Food Chemistry, TUM School of Life Sciences, Technical University of Munich, Munich, Germany; 8 Institute of Experimental Genetics, Helmholtz Zentrum München, German Research Center for Environmental Health, Neuherberg, Germany; 9 Chair of Experimental Genetics, TUM School of Life Sciences, TUM, Freising, Germany; 10 Department of Biochemistry, Yong Loo Lin School of Medicine, National University of Singapore, Singapore, Singapore; 11 Institute of Biochemistry, Faculty of Medicine, University of Ljubljana, Ljubljana, Slovenia; 12 Institute of Laboratory Medicine, University Hospital, LMU Munich, Munich, Germany; 13 German Centre for Cardiovascular Research (DZHK), Partner Site Munich Heart Alliance, Munich, Germany; 14 Institute for Medical Information Processing, Biometry, and Epidemiology, Pettenkofer School of Public Health, Ludwig Maximilian University of Munich (LMU), Munich, Germany

**Keywords:** Liver fibrosis, Fibrosis-4 index, Male subfertility, Sodium-glucose co-transporter 2 inhibitors (SGLT2i), Metformin, Type 2 diabetes, Metabolic dysfunction-associated steatotic liver disease (MASLD), Hydroxybutyrylcarnitine (C4-OH), Threonine, Urea cycle

## Abstract

**Background:**

Sodium-glucose co-transporter 2 inhibitors (SGLT2i), when combined with metformin (COMBI), offer multi-organ protective effects in patients with type 2 diabetes (T2D), particularly those at high risk of cardiovascular or renal complications. However, the underlying molecular mechanisms remain poorly understood.

**Methods:**

We profiled 303 targeted serum metabolites in 1494 participants of the KORA study, including T2D patients treated with COMBI therapy, metformin monotherapy, or no glucose-lowering medication. Additionally, metabolomic profiling was quantified on seven tissues (plasma, liver, adrenal glands, adipose tissue, testis, lung, and cerebellum), and related hepatic transcripts were evaluated in 40 mice. Multivariable linear regression analyses, adjusted for age, sex, BMI, lifestyle, glycemic, and cardiovascular risk factors, were applied to human data; tissue-specific regression analyses were conducted for murine samples. Identified metabolites were further investigated using biochemical pathway analyses and literature review.

**Results:**

COMBI therapy was associated with significant changes in metabolite profiles. In humans, 10 metabolites were significantly altered compared to metformin monotherapy. In mice, 82 altered metabolites were identified in plasma, 52 in liver, 30 in adrenal glands, 12 in adipose tissue, seven in testis, seven in lung, and six in cerebellum. COMBI therapy lowered threonine concentrations in both human serum and murine plasma but raised threonine, glycine, and urea cycle metabolites (citrulline, asymmetric dimethyl arginine (ADMA), and ornithine) in murine liver. This was accompanied by enhanced hepatic expression of *Slc38a2,* a threonine transporter gene. In humans, urea cycle metabolites correlated strongly with the fibrosis-4 index, a marker of liver fibrosis. Additionally, COMBI therapy elevated ketone body markers, such as hydroxybutyrylcarnitine, across murine liver, plasma, adrenal glands, adipose tissue, and testis.

**Conclusions:**

COMBI therapy modulates amino acid metabolism, the urea cycle, and ketone body production, suggesting potential mechanisms underlying its protective effects against liver fibrosis and male subfertility. These findings provide novel insights into the systemic metabolic actions of COMBI therapy and highlight its translational potential to improve clinical outcomes in T2D patients.

**Supplementary Information:**

The online version contains supplementary material available at 10.1186/s12916-025-04609-7.

## Background

Type 2 diabetes (T2D) is a metabolic disorder associated with systemic complications that increase comorbidities and mortality [1]. Effective management of T2D requires achieving optimal glycemic control while preventing systemic complications [2]. Pharmacological interventions play a pivotal role in these efforts [3]. Clinical guidelines recommend metformin as the first-line therapy for T2D, with sodium-glucose co-transporter 2 inhibitors (SGLT2i) as an add-on therapy for patients at high risk of cardiovascular and renal complications [2, 4]. Beyond their cardio-renal protective properties, SGLT2i have demonstrated additional tissue-protective effects, such as attenuating metabolic dysfunction-associated steatotic liver disease (MASLD), improving testicular function, and suppressing sympathetic nerve activity [5–8]. However, the molecular mechanisms underlying these tissue-protective effects remain poorly understood.

Metabolomics is a powerful tool for elucidating molecular mechanisms and identifying molecular signatures associated with pharmacological interventions [9, 10]. Previous studies employing untargeted metabolomics have investigated the tissue-specific effects of SGLT2i in combination with metformin (COMBI) across multiple tissues in mice (e.g., plasma, kidney, liver, muscle, and epididymal adipose tissue) and in human serum [11, 12]. These studies demonstrated that COMBI therapy mitigates adverse effects associated with SGLT2i and metformin monotherapies, such as protein catabolism, diabetic ketoacidosis, and lactic acidosis [11], and revealed bidirectional modulation of the TCA cycle, including its anaplerosis [12].

While these findings have provided valuable insights into COMBI therapy’s metabolic effects, targeted metabolomics offers a more direct and quantitative evaluation of specific metabolites [13–16]. This approach allows for detailed dissection of tissue-specific pathways, advancing our understanding of physiological and pathological metabolic processes [17, 18]. Further investigation using targeted metabolomics is essential to comprehensively characterize the metabolic effects of COMBI therapy and to evaluate the translatability of murine findings to humans with T2D.

**Fig. 1:**
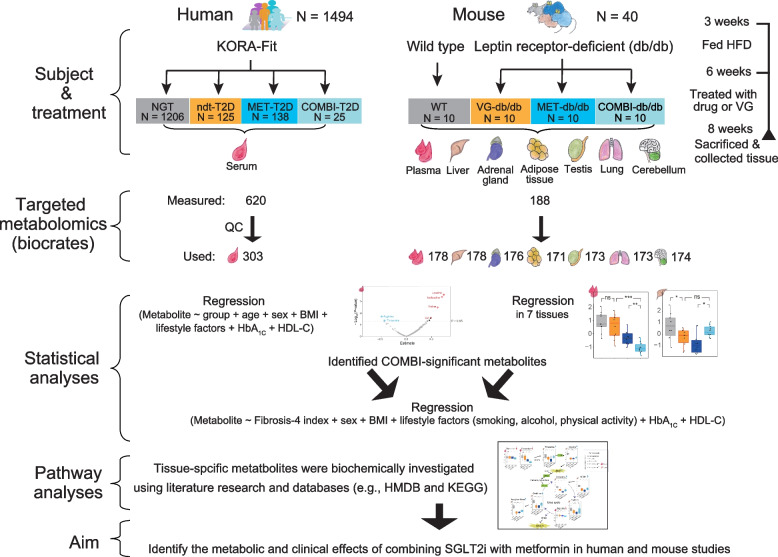
Study design. Abbreviations: BMI, body mass index; COMBI, SGLT2 inhibitor and metformin-treated; HbA_1C_, hemoglobin A1C; HDL-C, high-density lipoprotein cholesterol; HFD, high-fat diet; MET, metformin-treated; ndt-T2D, non-antidiabetic drug treated type 2 diabetes; NGT, normal glycemic tolerance; VG, vehicle-gavaged; WT, wild type; HMDB, human metabolomics database; KEGG, Kyoto Encyclopedia of Genes and Genomes

In this study, we applied targeted metabolomics to explore the metabolic effects of COMBI therapy: (1) in serum, by comparing T2D patients treated with COMBI therapy or metformin monotherapy in the Cooperative Health Research in the Region of Augsburg (KORA)-Fit study (Fig. 1) [19]. COMBI-significant metabolites were further evaluated in non-antidiabetic drug-treated T2D patients (ndt-T2D) and individuals with normal glucose tolerance (NGT); (2) across multiple tissues (plasma, liver, adrenal gland, adipose tissue, testis, lung, and cerebellum) of leptin receptor-deficient db/db mice treated with COMBI (COMBI-db/db), metformin alone (MET-db/db), vehicle (VG-db/db), or wild-type mice (WT) [11, 12]; and (3) by investigating urea cycle-related metabolites in relation to the fibrosis-4 index in humans to advance our understanding of the systemic benefits of COMBI therapy (Fig. 1).

## Methods

### Human study

This study utilized data from KORA-Fit, a follow-up survey conducted between 2018 and 2019 [19]. KORA (Cooperative Health Research in the Region of Augsburg) is a population-based cohort conducted in the Augsburg region of southern Germany [17]. The KORA cohort consists of four baseline surveys: S1 (1984–1985), S2 (1989–1990), S3 (1994–1995), and S4 (1999–2000), comprising a total of 18,079 participants. The KORA-Fit follow-up study invited all individuals from these four surveys who were aged 53–74 years during the 2018/2019 follow-up period [19].

Targeted metabolomics analyses were performed on serum samples from 2956 KORA-Fit participants between May 2021 and March 2022. After excluding participants receiving insulin and/or incretin-based therapies or those with prediabetes, a total of 1494 participants were included in this study (Fig. 1 and Table 1).

**Table 1:** Characteristics of the four groups of KORA-Fit participants

	Groups				*P*-value^§^		
	NGT	ndt-T2D	MET-T2D	COMBI-T2D	COMBI-T2D vs. MET-T2D	COMBI-T2D vs. ndt-T2D	ndt-T2D vs. NGT
N	1206	128	138	25			
Age, years	62.1 ± 5.5	64.7 ± 5.1	65.8 ± 5.0	64.7 ± 5.9	0.45	0.97	**2.48 × 10**^**−07**^
At T2D diagnosis, years	NA	58.0 ± 5.4	58.5 ± 7.4	55.5 ± 8.2	0.10	0.25	-
Duration of T2D, years	NA	6.6 ± 4.9	7.8 ± 6.2	9.5 ± 6.1	0.14	0.22	-
Male, %	39.5	57.8	55.8	40.0	0.19	0.13	**9.07 × 10**^**−05**^
BMI, kg/m^2^	26.5 ± 4.5	31.1 ± 5.9	30.8 ± 5.3	32.2 ± 7.4	0.30	0.39	**5.13 × 10**^**–20**^
**Lifestyle**							
Current smoking, %	13.7	14.1	10.9	12.0	0.74	1.00	0.89
Alcohol high intake^‡^, %	18.6	18.8	6.5	12.0	0.40	0.57	1.00
Physically active^†^, %	76.2	61.7	63.8	76.0	0.26	0.25	**1.00 × 10**^**−03**^
**Laboratory test**							
Serum albumin, g/L	4.6 ± 0.2	4.7 ± 0.2	4.6 ± 0.2	4.7 ± 0.2	0.62	0.53	0.45
AST, IU/L	24.7 ± 9.9	27.8 ± 12.4	27.9 ± 14.7	24.0 ± 6.5	0.28	0.14	**0.03**
ALT, IU/L	24.7 ± 12.2	34.1 ± 20.2	34.4 ± 18.8	31.7 ± 18.6	0.46	0.59	**2.08 × 10**^**–11**^
Fibrosis-4 index	1.5 ± 0.6	1.6 ± 0.8	1.5 ± 0.7	1.4 ± 0.6	0.78	0.33	0.28
eGFR, mL/min/1.73 m^2^	82.8 ± 12.1	79.1 ± 14.1	80.1 ± 14.6	75.4 ± 16.5	0.15	0.27	**7.00 × 10**^**−03**^
Total cholesterol, mg/dL	216.7 ± 39.5	199.9 ± 43.0	190.9 ± 39.0	196.3 ± 50.2	0.74	0.71	**1.29 × 10**^**−05**^
HDL-C, mg/dL	68.8 ± 18.8	56.1 ± 16.1	54.2 ± 14.4	57.5 ± 14.1	0.29	0.69	**1.97 × 10**^**–14**^
LDL-C, mg/dL	126.5 ± 35.3	114.1 ± 38.3	105.9 ± 34.9	108.2 ± 44.3	0.69	0.50	**2.46 × 10**^**−04**^
TG, mg/dL	108.2 ± 61.2	150.3 ± 81.1	157.5 ± 89.4	167.7 ± 93.7	0.43	0.34	**5.81 × 10**^**−13**^
hs-CRP, mg/L	2.2 ± 3.5	3.5 ± 4.6	2.9 ± 2.6	2.9 ± 3.0	0.72	0.63	**2.15 × 10**^**−08**^
Fasting glucose, mg/dL	91.7 ± 5.0	128.0 ± 23.3	138.1 ± 34.2	150.5 ± 34.8	0.05	**1.18 × 10**^**−03**^	**9.23 × 10**^**−70**^
HbA_1C_, %	5.3 ± 0.3	6.2 ± 0.7	6.7 ± 1.0	7.3 ± 0.8	**1.33 × 10**^**−04**^	**4.33 × 10**^**−09**^	**8.79 × 10**^**−56**^
Hypertension, %	37.6	76.6	81.6	80.0	0.79	0.80	**2.34 × 10**^**−17**^
**Medications**							
Anti-hypertensives, %	31.6	65.6	77.5	80.0	1.00	0.24	**9.25 × 10**^**–14**^
Fibrate, %	0.2	1.6	0.7	0.0	1.00	1.00	0.08
Statin, %	10.2	26.6	35.5	48.0	0.27	0.05	**8.95 × 10**^**−07**^
**Complications**							
Angina, %	4.1	5.5	5.1	8.0	0.63	0.64	0.48
Myocardial infarction, %	1.6	8.6	6.5	8.0	0.68	1.00	**4.66 × 10**^**−**05^
Stroke, %	1.7	6.2	6.5	0.0	0.36	0.36	**4.00 × 10**^**−03**^

Clinical and laboratory characteristics of the four groups of KORA-Fit participants are shown: normal glucose tolerance (NGT), non-antidiabetic drug treated type 2 diabetes (ndt-T2D), metformin-treated type 2 diabetes (MET-T2D), and metformin and SGLT2 inhibitor-treated type 2 diabetes (COMBI-T2D). For each variable, *P*-values for comparisons between the groups (COMBI-T2D vs. MET-T2D, COMBI-T2D vs. ndt-T2D, and ndt-T2D vs. NGT) are presented

*Abbreviations*: *ALT* alanine aminotransferase, *AST* aspartate aminotransferase, *BMI* body mass index, *HbA*_*1C*_ hemoglobin A1c, *HDL-C* high-density lipoprotein cholesterol, *hs-CRP* high sensitivity C-reactive protein, *LDL-C* low-density lipoprotein cholesterol, *TG* triglycerides

Additional notes:

^†^ > 1 h per week

^‡^ > 40 g/day in men; > 20 g/day in women

^§^ Continuous variables are presented as mean ± standard deviation (SD), and categorical variables are presented as percentages (%). Comparisons between COMBI-T2D and MET-T2D, COMBI-T2D and ndt-T2D, and ndt-T2D and NGT for continuous variables were performed using the Mann–Whitney *U* test, while comparisons for categorical variables were performed using the chi-square test. A *P*-value < 0.05 was considered statistically significant and is shown in bold

### Mouse study

The study used male C57BL6J-based BKS.Cg-m + / + Lepr m/m wild-type and C57BL6-based BKS.Cg-Dock7m + / + Leprdb/J db/db mice, a common T2D model. The mice were housed in a pathogen-free facility under light- and temperature-controlled conditions, following FELASA (Federation of Laboratory Animal Science Associations) guidelines.

Wild-type mice (*N* = 10) received a standard diet and vehicle treatment (5% solutol and 95% hydroxyethylcellulose) starting at 6 weeks of age. db/db mice were fed a high-fat diet (HFD) starting at 3 weeks of age [11, 12]. At 6 weeks, db/db mice were assigned to three treatment groups for 14 days: vehicle (VG, *N* = 10), metformin (MET, *N* = 10; 300 mg/kg), or combination therapy (COMBI, *N* = 10; 30 mg/kg SGLT2 inhibitor + 300 mg/kg metformin) [20]. Treatments were administered once daily via gavage prior to the dark phase.

Following the 14-day treatment, mice were fasted for 4 h, sacrificed by isoflurane overdose, and tissues (blood, liver, adrenal glands, epididymal adipose tissue, testis, lungs, and cerebellum) were collected. Plasma was prepared by centrifugation, and tissues were freeze-clamped and stored at − 80 °C. Additional details are described in prior publications [4, 10].

### Metabolomics analyses and quality control in human samples

Serum samples from the KORA-Fit study (*N* = 2956) were analyzed using the MxP® Quant 500 kit (biocrates Life Sciences AG, Innsbruck, Austria), which quantifies 620 metabolites across 40 kit plates. Each plate included three negative and nine positive quality control (QC) samples. The negative controls consisted of PBS-based zero samples, which were used to define background noise and calculate the limit of detection (LOD). The positive QC samples included three manufacturer-provided plasma samples (QC1, QC2, QC3), one plasma sample from the National Institute of Standards and Technology (NIST), and five reference plasma samples. These positive controls were used to assess metabolite variation and calculate the relative standard deviations (RSD, also known as coefficients of variation, CV) [21].

Metabolites were included in the analysis if they fulfilled the following criteria: (1) At least 50% of the 2956 measured sample values were above the LOD. (2) The median RSD (or CV) across QC1, QC2, QC3, NIST, and reference samples was < 25%. (3) Fewer than 10% of the values were missing in the total dataset of 2956 samples from the KORA-Fit study (Additional file 1: Table S1).

To minimize technical variation, metabolite concentrations were normalized using the non-parametric TIGER method, which employs a flexible ensemble learning architecture [22]. Missing data were imputed using GSimp [23], and the data were subsequently log-transformed and standardized (mean = 0, SD = 1) to ensure comparability across analyses.

### Metabolomics analyses in mouse samples

Mouse plasma, liver, adrenal gland, adipose tissue, testis, lung, and cerebellum samples were analyzed using the Absolute*IDQ* ® p180 Kit (biocrates Life Sciences AG, Innsbruck, Austria), quantifying 188 metabolites. Tissue preparation and LC–MS/MS methods were described previously [18, 24]. Metabolites with > 20% missing values and samples with > 10% missing metabolites were excluded.

Plate correction was not necessary because db/db and WT mouse samples of the same tissue type were measured on the same kit plate. Missing data were imputed using MICE [25], and metabolite concentrations were log-transformed standardized (mean = 0, SD = 1) for each tissue.

### Transcriptomics analyses in mouse samples

Transcriptomics analyses were conducted following previously established procedures [26–28]. Hepatic mRNA was extracted and hybridized to Illumina MouseRef-8 v2 BeadChip Arrays (Illumina, San Diego, CA, USA), followed by scanning on an Illumina HiScan system. Raw intensity data were processed on an Illumina GenomeStudio V2009.1, where background subtraction was applied and an offset added to ensure positive linear expression values.

Data normalization was performed using CARMAweb 1.5, which implements the quantile normalization algorithm. Probe sets with counts > 50 in at least one experimental group were retained for further analysis. Expression data were log-transformed prior to statistical analyses. Gene expression probes were included if they showed a detection *P*-value < 0.05 in ≥ 25% of the samples.

### Statistical analysis

Multivariable linear regression was used to identify metabolite associations through pairwise comparisons (COMBI-T2D vs. MET-T2D, MET-T2D vs. ndt-T2D, and ndt-T2D vs. NGT). All primary models were adjusted for age, sex, body mass index (BMI), smoking status, alcohol intake, physical activity, hemoglobin A_1C_ (HbA_1C_), and high-density lipoprotein cholesterol (HDL-C). The regression model was specified as:

*Y*_metabolite concentration_ ∼ *X*_groups_ + age _years_ + sex _female = 0, male = 1_ + BMI _kg/m_^2^ + smoking status _current smoker = 1, otherwise = 0_ + alcohol intake _male: >40 g/week or female: >20 g/week = 1, otherwise =0_ + physical activity _active = 1, otherwise = 0_ + HbA_1C %_ + HDL-C _mg/dL_.

To evaluate the robustness of the associations identified between COMBI-T2D and MET-T2D, six sensitivity models were conducted: S-model 1 adjusted for age and sex; S-model 2 additionally adjusted for smoking and physical activity; S-model 3 additionally adjusted for BMI; S-model 4 additionally adjusted for HDL-C and fasting glucose; S-model 5 additionally adjusted for alcohol consumption and hypertension; and S-model 6 applied the primary model without HbA_1C_.

The association between the fibrosis-4 index and urea cycle metabolites was also analyzed using multivariable linear regression, adjusted for the same covariates, including metabolite concentration as the dependent variable and fibrosis-4 index as predictors. The fibrosis-4 index, calculated using age, aspartate aminotransferase (AST), alanine aminotransferase (ALT), and platelet count, was used to assess liver fibrosis with a cutoff of 1.3 [29].

In the mouse study, pairwise linear regression was performed for each metabolite, with concentration values as the outcome and grouping comparisons (e.g., COMBI vs. MET, MET vs. VG, and VG vs. WT) as predictors.

To account for multiple testing, Bonferroni cutoffs were applied (*P* < 1.65 × 10^−04^ for 303 human metabolites; *P* < 2.92 − 2.81 × 10^−04^ for 176–178 murine metabolites across tissues). Statistical significance was assessed using false discovery rate (FDR < 0.05) and nominal *P* < 0.05.

Pairwise comparisons of gene expression between mouse groups were performed using the limma moderated t-tests, incorporating surrogate variable analysis to adjust for hidden batch effects [30]. Relative expression levels were calculated by normalizing each gene’s expression of the WT group, which was set to 1. Statistical significance was determined using a Bonferroni-adjusted threshold of *P* < 4.03 × 10^−06^.

All analyses were performed in R (version 4.3.2) [31].

### Pathway analysis

Pathway analyses were conducted using databases such as the Human Metabolome Database (HMDB) and the Kyoto Encyclopedia of Genes and Genomes (KEGG) [32, 33], with consideration of tissue and organ specificity, supplemented and double-checked by deep literature research.

## Results

### Characteristics of human and mouse studies treated with and without COMBI therapy

Participants from the KORA-Fit study (*N* = 1494) were analyzed for 303 metabolites to systematically assess metabolic changes across four groups: 1206 individuals with NGT, 125 ndt-T2D, 138 MET-T2D, and 25 COMBI-T2D (Fig. 1). COMBI-T2D patients displayed clinical characteristics comparable to the metformin-treated T2D group (Table 1). For example, the two groups had similar average age (64.7 vs. 65.8 years), age at T2D diagnosis (55.5 vs. 58.5 years), and T2D duration (9.5 vs. 7.8 years). Both groups also shared comparable lifestyle factors (smoking, alcohol intake, and physical activity), liver and kidney functions (serum albumin, AST, ALT, fibrosis-4 index, and estimated glomerular filtration rate [eGFR]), cholesterol levels (total, HDL-C, low-density lipoprotein (LDL-C), and triglycerides (TG)), inflammatory status (high sensitivity C-reactive protein (hs-CRP)), use of medications (anti-hypertensives, fibrates, and statins), and prevalence of complications (angina, myocardial infarction, and stroke). However, a significant difference was noted in HbA_1C_ levels, which were higher in the COMBI-T2D patients compared to the metformin group, despite similar fasting glucose values. In addition, COMBI-T2D patients displayed clinical characteristics comparable to the ndt-T2D group, while fasting glucose and HbA_1C_ levels were significantly higher in COMBI-T2D than those in the ndt-T2D group (Table 1).

In contrast, in db/db mice treated with COMBI therapy, a significant reduction in blood glucose levels was observed after 2 weeks of treatment. Blood glucose levels in the COMBI-db/db group decreased by 70.1%, whereas the following reductions were observed in the other groups: 1.9% in WT, 4.7% in VG-db/db, and 29.1% in MET-db/db (Additional file 1: Table S2).

### Identification of COMBI-significant metabolites in human and mouse blood

**Fig. 2:**
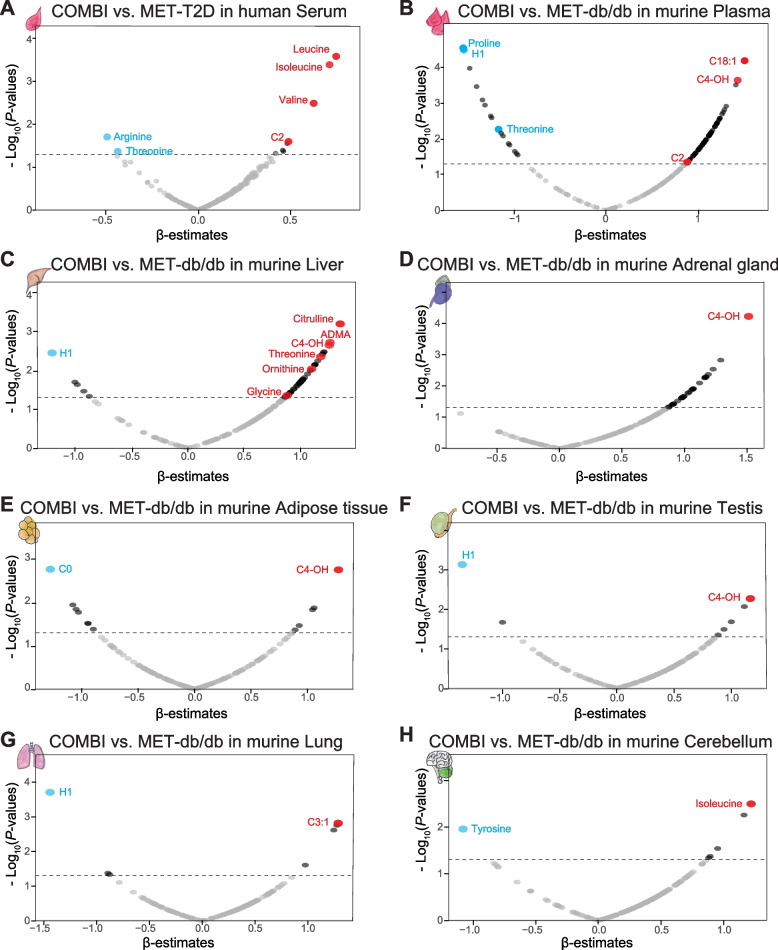
Identification of COMBI-significant metabolites in human serum and seven murine tissues. **A** Volcano plot of multivariate linear regression analysis results (β-estimates and *P*-values) for 303 serum metabolites in the pairwise comparison of COMBI-treated T2D patients versus MET-treated T2D patients. B-H: Volcano plots of linear regression analysis results (β-estimates and *P*-values) in pairwise comparisons of COMBI-treated db/db mice versus MET-treated db/db mice across seven tissues: 178 plasma metabolites (**B**), 178 liver metabolites (**C**), 176 adrenal gland metabolites (**D**), 171 adipose tissue metabolites (**E**), 173 testis metabolites (**F**), 173 lung metabolites (**G**), and 174 cerebellum metabolites (**H**). The gray dashed line represents a *P*-value threshold of 0.05

Out of the 303 metabolites analyzed, 10 were found as significantly different between COMBI-T2D compared to MET-T2D in the primary multivariable adjustment model, which included physiological variables (age, sex, BMI), lifestyle factors (smoking status, alcohol intake, physical activity), and clinical variables (HbA_1C_ and HDL-C) (Fig. 2A, Additional file 1: Tables S3, S4). Among the identified metabolites, branched-chain amino acids (BCAAs) such as leucine, isoleucine, and valine, were positively associated, while arginine and threonine showed negative associations with COMBI-T2D (Fig. 2A and Additional file 1: Table S4).

To further evaluate whether the 10 identified metabolites were specific to the COMBI-T2D group, we performed pairwise comparisons between COMBI-T2D and ndt-T2D, as well as between ndt-T2D and NGT. We found that six metabolites were significantly different between the COMBI-T2D and ndt-T2D groups, whereas only deoxycholic acid (DCA) showed a nominal difference between ndt-T2D and NGT. This suggests that the nine identified COMBI-metabolites are independent of diabetes progression (Additional file 1: Table S4).

**Fig. 3:**
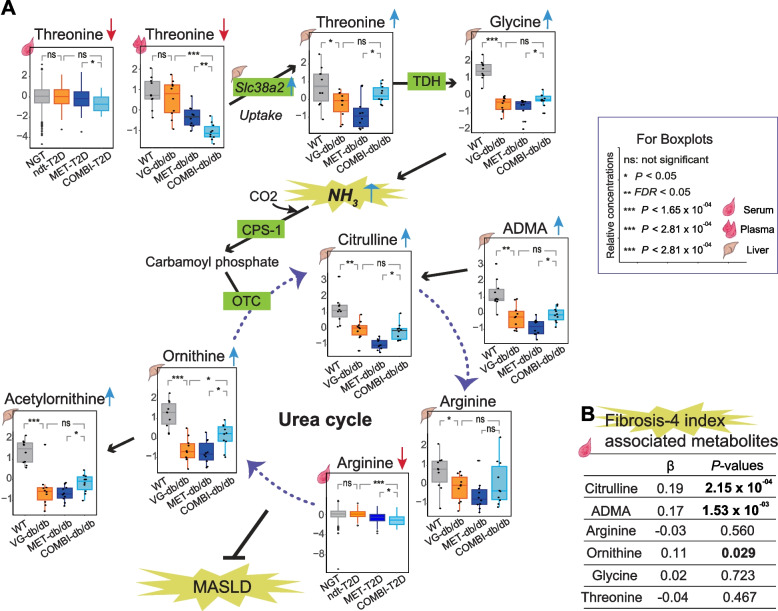
Threonine metabolism, urea cycle pathways, and fibrosis-4 index associations with metabolites in human and murine models. **A** Threonine metabolism and urea cycle-associated pathways are shown in relation to T2D and MASLD complications. Boxplots display selected metabolites levels across four groups in human serum (NGT, ndt-T2D, MET-T2D, and COMBI-T2D) and murine plasma and liver (WT, VG-db/db, MET-db/db, and COMBI-db/db). **B** Multivariate regression results (β-estimates and *P*-values) are presented for six serum metabolites associated with the fibrosis-4 index, adjusted for sex, BMI, lifestyle factors, HbA1C and HDL-C. Abbreviations: ADMA asymmetric dimethylarginine; CAT, carnitine acetyltransferase; CO2, carbon dioxide; CPS-1, carbamoyl phosphate synthetase 1; C4-OH, hydroxybutyrylcarnitine; MASLD, metabolic dysfunction-associated steatotic liver disease; NH3, ammonia; OTC, ornithine transcarbamylase; TDH, threonine dehydrogenase

In the mouse model, regression analyses comparing COMBI-db/db and MET-db/db groups revealed 82 nominally significant plasma metabolites (Fig. 2B and Additional file 1: Table S5). While BCAAs had comparable concentrations between COMBI-db/db and MET-db/db groups in plasma, threonine and acetylcarnitine (C2) were consistently observed in both human and mouse studies. Specifically, threonine levels were comparable between WT and VG-db/db mice, whereas metformin-treated db/db mice had significantly lower values, and the COMBI-db/db group had the lowest concentration of threonine in murine plasma (see boxplot in Fig. 3A and Additional file 1: Table S5).

### Identification of COMBI-significant metabolites in six additional murine tissues

Comparative analyses between COMBI-db/db and MET-db/db identified 52 nominally significant metabolites in the liver (Fig. 2C and Additional file 1: Table S6), 30 in the adrenal gland (Fig. 2D and Additional file 1: Table S7), 12 in adipose tissue (Fig. 2E and Additional file 1: Table S8), seven each in the testis and lung (Fig. 2F, G and Additional file 1: Tables S9, S10), and six metabolites in the cerebellum (Fig. 2H and Additional file 1: Table S11).

In three murine tissues (liver, testis, and lung), as well as in plasma, H1 (hexoses including glucose) was significantly negatively associated with COMBI-db/db, consistent with the clinical observation of reduced glucose levels following 2 weeks of treatment in db/db mice.

In the liver, out of 52 COMBI-significant metabolites, 47 (approximately 90%) showed positive associations with COMBI-db/db, including threonine, glycine, and urea cycle-related metabolites (ornithine, acetylornithine, and asymmetric dimethylarginine, [ADMA]) (Additional file 1: Table S6). For example, the concentration of threonine in the liver of the COMBI-db/db group was higher than in the MET-db/db group, which is opposite to the pattern observed in circulating blood in both mice and humans (Fig. 2A–C and Additional file 1: Tables S4–S6). Moreover, WT mice had the highest levels of threonine, while VG-db/db mice had lower levels, and metformin-treated mice had the lowest levels. The combination therapy significantly reverted threonine levels in the liver (see boxplot in Fig. 3A). A similar pattern of reversion was also observed for glycine in the liver (see boxplot in Fig. 3A).

**Fig. 4:**
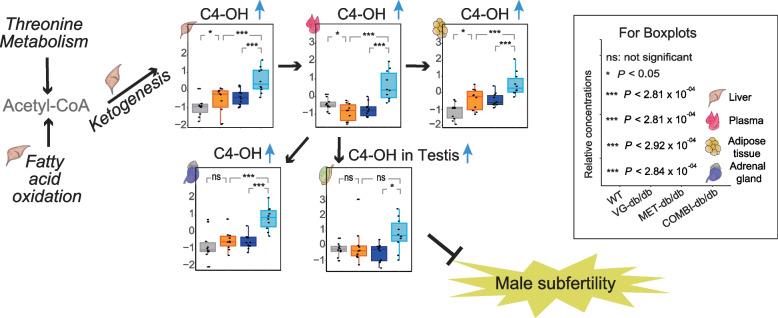
Ketogenesis and C4-OH metabolic pathway in systemic tissues of mice suggest protection against T2D-associated male subfertility. A schematic overview illustrates the role of ketogenesis and the effects of COMBI treatment on C4 OH (hydroxybutyrylcarnitine) levels in the liver, plasma, adrenal gland, adipose tissue, and testis. Boxplots display C4-OH levels across four groups of mice: WT and three db/db groups

Of particular note, C4-OH (hydroxybutyrylcarnitine, also referred to as C3-DC or malonylcarnitine) was consistently associated with COMBI-db/db across five tissues: plasma, liver, adrenal gland, adipose tissue, and testis. This highlights its relevance across multiple biological compartments (Fig. 2B–F and Additional file 1: Tables S5–S9). Furthermore, C4-OH concentrations in the COMBI-db/db mice were higher in all five tissues compared to the three investigated groups: WT, VG-db/db, and MET-db/db (Fig. 4).

### Identification of threonine metabolism and urea cycle pathway-associated gene expressions

In the liver, among the 24 transcripts significantly altered by COMBI therapy, *Slc38a2* was notably linked to threonine metabolism. *Slc38a2* encodes a well-characterized sodium-dependent transporter for neutral amino acids, including threonine, and mediates their uptake into hepatocytes. Hepatic *Slc38a2* expression was significantly higher in the COMBI-db/db group compared with the metformin-db/db group (*P* = 4.08 × 10^−8^; Additional file 1: Fig. S1, Table S12). Similarly, *Slc38a2* expression in the COMBI-db/db group was significantly higher than in the VG-db/db group, while expression levels were comparable between the VG-db/db and WT groups (Additional file 1: Table S12).

In contrast, the other three genes associated with threonine metabolism, *Shmt2*, *Shmt1*, and *Gldc*, did not show significant drug effects but displayed marked differences between the VG-db/db and WT groups (Additional file 1: Table S12). These findings suggest that COMBI therapy selectively upregulates hepatic *Slc38a2* expression, whereas the expression of other threonine metabolism–related genes remains primarily influenced by the diabetic state rather than by drug treatment.

### Identification of fibrosis-4 index associated serum metabolites of the urea cycle

Multivariable linear regression analysis revealed a significant positive association between the fibrosis-4 index, an indicator of liver fibrosis, and metabolites in the urea cycle and related pathways. Specifically, serum levels of citrulline, ADMA, and ornithine, but not arginine, glycine, or threonine, were significantly positively associated with the fibrosis-4 index in the studied KORA-Fit participants, including individuals with NGT, ndt-T2D, MET-T2D, and COMBI-T2D (Fig. 3B).

### Sensitivity analyses

Given the relatively small sample size in the COMBI group (*N* = 25), we first evaluated the reliability of the findings using post hoc power analyses for the 10 metabolites that differed significantly between the COMBI-T2D and MET-T2D (Additional file 1: Table S13). Four metabolites showed strong effect sizes and adequate statistical power (> 0.80), three showed moderate power (0.61–0.73), and three had limited power (< 0.55). Isoleucine, leucine, valine, and TG (20:4_36:4) demonstrated the strongest effects (Cohen’s *d* ≈ 0.6–0.7). These results support the robustness of the major associations, although metabolites with lower power, particularly C2, DCA, and 3-IAA, should be interpreted cautiously.

To further assess robustness, six additional sensitivity models were applied to the 10 COMBI-associated metabolites, each incorporating incremental adjustments for different sets of potential confounders (Additional file 1: Table S14). Across these models, most metabolites remained statistically significant, and their β estimates were consistent in both magnitude and direction. Arginine, isoleucine, leucine, and valine showed stable associations across all models. Some metabolites, including C2 and TG (20:4_36:4), exhibited attenuated significance in the fully adjusted S-mode 5, although their effect sizes and directions remained comparable. In contrast, 3-IAA became significant in both S-model 5 and S-model 6.

Given the substantial difference in sample size between the COMBI-T2D and MET-T2D groups, we additionally performed propensity score (PS) matching using a 1:2 ratio (COMBI:MET). After matching, HbA_1C_ remained slightly higher in the COMBI group; however, the standardized mean difference (SMD) was < 0.2, and all other covariates were well balanced (SMD < 0.1) (Additional file 1: Fig. S2A). Multivariate regression using the primary adjustment model in the PS matched samples further confirmed that seven metabolites (threonine, arginine, valine, isoleucine, leucine, C2, and TG (20:4_36:4)) remained significantly associated with COMBI therapy (Additional file 1: Fig. S2B).

## Discussion

Combined SGLT2i and metformin therapy lowered circulating threonine levels in both T2D patients and db/db mice compared with metformin monotherapy. In contrast, hepatic threonine, glycine, and urea cycle metabolites were higher in mice, accompanied by upregulation of the threonine transporter gene *Slc38a2*. These findings suggest enhanced hepatic threonine uptake and metabolism under combination therapy. The strong correlations of hepatic and circulating urea cycle-related metabolites with the fibrosis-4 index support the possibility that this metabolic shift contributes to protection against MASLD and liver fibrosis. In parallel, the observed enhancement of fatty acid oxidation and ketogenesis was reflected by elevated C4-OH levels across multiple tissues, particularly the testis, suggesting a potential protective role against male subfertility (Fig. 4).

Biochemically, circulating threonine is taken up by the liver through neutral amino acid transporters encoded by *Slc38a2* and metabolized into glycine via threonine dehydrogenase (TDH), a reaction that generates ammonia (NH₃) as a byproduct [34]. Ammonia is subsequently processed by carbamoyl phosphate synthetase I (CPS-1) and incorporated into the urea cycle, primarily within hepatocytes [35, 36]. In our study, combination therapy resulted in higher hepatic threonine and glycine levels, leading to increased ammonia production and urea cycle flux. Although circulating threonine concentrations were lower in combination therapy-treated mice and humans than their respective metformin monotherapy groups, hepatic *Slc38a2* expression was significantly upregulated, consistent with enhanced hepatic uptake of threonine. All five urea cycle-related metabolites (citrulline, ornithine, arginine, acetylornithine, and ADMA) were highest in the WT group, lowered in the VG-db/db and MET-db/db groups, and restored by the combination therapy (Fig. 3A). The lowest serum arginine level observed in the COMBI-T2D group may reflect its increased utilization within the liver. Overall, these findings indicate that the combination therapy enhances urea cycle activity, which may contribute to protection against MASLD [37]. Furthermore, enhanced urea cycle flux could reduce NO production from arginine, thereby limiting NO-mitigated inflammatory and cytotoxic effects and potentially contributing to the attenuation of MASLD [38].

Our findings align with recent work highlighting hierarchical regulation of the urea-TCA cycle and its disruption in fibroinflammatory liver diseases, including MASLD [39]. Previously, we reported that combination therapy modulates multiple metabolic pathways related to glutaminolysis (hepatic glutamate), tumorigenesis, and anti-inflammation (kidney 2-HG), anti-oxidation (hepatic taurine), and urea/NO cycle perturbations (hepatic citrulline) [12]. In the current study, targeted metabolomics in human and mouse consistently identified threonine metabolism as a central node influenced by combination therapy. The observed associations of citrulline, ADMA, and ornithine with the fibrosis-4 index in human serum further support the hypothesis that impaired urea cycle metabolism contributes to liver fibrosis progression, and that combination therapy may mitigate this progression by restoring hepatic metabolite levels.

Beyond hepatic effects, we observed consistently higher concentrations of C4-OH in COMBI-treatment mice across multiple tissues, including plasma, liver, adrenal gland, adipose tissue, and testis. Elevated levels of C4-OH reflect increased fatty acid oxidation and ketogenesis, as C4-OH is co-produced with ketone bodies [40]. Our results suggest that enhanced ketogenesis in the liver leads to elevated ketone body levels in plasma, subsequently contributing to increased ketone levels in systemic tissues such as adipose tissue, adrenal glands, and testis (Fig. 4). High levels of ketone bodies such as β-hydroxybutyrate in the adrenal gland may suppress sympathetic nerve hyperactivity via inhibition of G protein-coupled receptor 41, potentially reducing heart rate, blood pressure, and vascular resistance [41–44]. In the testis, the high ketone body levels induced by combination therapy may have protective effects against male subfertility. T2D is a well-known cause of male subfertility, with a prevalence of 35.1% in diabetic men compared to non-diabetic men [45], and a causal relationship has also been identified by Mendelian randomization [46]. SGLT2i has been reported to counteract hyperglycemia-induced inflammation, oxidative stress, and reductions in androgen-dependent testicular enzymes in the testis [7]. Ketone bodies exert anti-inflammatory effects (via suppression of the NLRP3 inflammasome and NF-κB) and anti-oxidative effects (via increased NAD + /NADH ratios) [44, 47, 48]. These mechanisms may underlie the combination therapy’s potential benefits for male subfertility. The observed highest concentration of C4-OH in COMBI-treated mice, compared to WT, VG, and MET-db/db mice, suggests a synergistic effect that could be particularly beneficial. This supports the need for future clinical investigations to evaluate the effects of combination therapy on male subfertility in T2D patients.

Taken together, the combination therapy modulates key mitochondrial processes, including threonine metabolism via TDH, as well as the urea cycle, fatty acid oxidation, and ketogenesis. These findings collectively suggest that the therapeutic effects of SGLT2i combined with metformin are mediated through mitochondrial metabolic regulation. Our previous finding on bidirectional modulation of TCA cycle anaplerosis further supports the hypothesis that mitochondria are central to the combination therapy’s mechanism of action [12].

### Limitations of this study

This study has several limitations. First, the targeted metabolomics platform (MxP® Quant 500 kit) cannot distinguish between certain isobaric or isomeric lipid species, such as hydroxybutyrylcarnitine (C4-OH) and malonylcarnitine (C3-DC). For simplicity, we use the terms C4-OH and/or “hydroxybutyrylcarnitine” throughout the text. Although C4-OH was excluded from the human dataset during QC due to a high median RSD of 34.13% across the nine positive plasma control samples measured over 11 months, serum concentrations of C4-OH were valid, with levels exceeding 50% of the LOD (Additional file 1: Table S1). Mouse tissues were analyzed on the same kit plate, eliminating plate effects [18]. Moreover, although plasma and serum metabolite profiles exhibit some differences, they are highly correlated [49]. Prior studies have also consistently reported elevated ketone body levels in patients treated with SGLT2 inhibitors [50, 51].

Second, the T2D patients treated with combination therapy had a relatively small sample size (N = 25), reflecting the number of eligible participants during the 2018–2019 collection period. Power analyses indicated adequate power for metabolites with strong effect sizes (e.g., isoleucine, leucine, valine, TG (20:4_36:4)) but limited power for metabolites such as C2, DCA, and 3-IAA. These should therefore be interpreted cautiously.

Third, causality cannot be inferred from our cross-sectional human data. The interpretation that combination therapy may reduce fibrosis via enhanced urea cycle activity remains speculative. Functional studies, such as hepatic stellate cell activation assays or fibrosis models assessing collagen deposition, are needed to validate causal pathways.

Fourth, although multivariable adjustment, correlation analyses, and multiple sensitivity analyses, including six statistical models and propensity score matching, indicated that the COMBI-associated metabolites are unlikely to be confounded by differences in HbA_1C_, some degree of residual confounding cannot be fully excluded in observational research. The higher HbA_1C_ levels in the COMBI group in the human cohort likely reflect differences in clinical context rather than treatment effects. In the KORA-Fit study (2018–2019), combination therapy was typically prescribed to patients with insufficient glycemic control on metformin monotherapy; thus, COMBI-treated patients generally had more advanced disease and greater difficulty achieving glycemic targets, contributing to baseline glycemic differences.

In contrast, mice in our experimental model began from comparable diabetic baselines, and subsequent HbA_1C_ differences reflected only pharmacological effects under controlled conditions. The discrepancy between human and mouse HbA_1C_ patterns therefore highlights the distinction between heterogeneous clinical populations and homogeneous laboratory models. Importantly, removing HbA_1C_ from the adjustment (S-model 6) did not alter the significance of the 10 COMBI-associated metabolites (Additional file 1: Table S14). This stability, together with their weak to moderate correlations with HbA_1C_ (*r* = − 0.18 to 0.22; Additional file 1: Table S15), indicates that these metabolite associations are unlikely to be driven solely by differences in glycemic control.

Fifth, model-related limitations must be considered. The leptin receptor-deficient db/db mouse reflects obesity-related insulin resistance but does not capture the full heterogeneity of human T2D, where varying levels of β-cell dysfunction, obesity, and comorbidities coexist. The 14-day treatment duration represents an early adaptive phase, and long-term effects remain uncertain. Future experiments using diet-induced obesity or β-cell dysfunction models, with longer treatment durations and dedicated fibrosis endpoints, will help evaluate chronic and translational relevance.

Finally, species-specific differences in SGLT2i metabolism may limit direct extrapolation from mice to humans. Empagliflozin undergoes predominantly oxidative metabolism in mice, whereas glucuronidation represents the major metabolic pathway in humans [52]. Although in vitro hepatocyte assays show minimal species differences, in vivo studies indicate interspecies variation in secretion and excretion patterns [53]. Therefore, the metabolic profile observed in mice may not fully reflect human pharmacokinetics.

## Conclusions

The combination of SGLT2 inhibitors and metformin enhances key mitochondrial processes, including threonine metabolism, the urea cycle, and ketogenesis. These metabolic changes may provide tissue-specific protective effects against T2D-associated complications, such as MASLD, liver fibrosis, and male subfertility. Our findings highlight the potential value of incorporating SGLT2i into first-line therapy alongside metformin for T2D patients at high risk of these complications.

## Supplementary Information

Additional file 1. Table S1. Quality control results for 620 metabolites in KORA-Fit. Table S2. Characteristics of the four mouse groups. Table S3. List of metabolites utilized in human serum and seven murine tissues. Table S4. Ten significant serum metabolites in COMBI vs. MET-T2D. Table S5. 82 significant plasma metabolites in COMBI vs. MET-db/db. Table S6. 52 significant liver metabolites in COMBI vs. MET-db/db. Table S7. 30 significant adrenal gland metabolites in COMBI vs. MET-db/db. Table S8. 12 significant adipose tissue metabolites in COMBI vs. MET-db/db. Table S9. Seven significant testis metabolites in COMBI vs. MET-db/db. Table S10. Seven significant lung metabolites in COMBI vs. MET-db/db. Table S11. Six significant cerebellum metabolites in COMBI vs. MET-db/db. Table S12. Threonine metabolism-associated liver transcripts in COMBI vs. MET-db/db. Table S13. Group comparisons between COMBI-T2D (N = 25) and MET-T2D (N = 138): effect sizes, confidence intervals, and post-hoc power. Table S14. Sensitivity analyses of the 10 metabolites in six models. Table S15. Correlation between HbA1C and 10 metabolites in T2D patients treated with COMBI or MET. Figure S1. Comparisons of the liver transcripts between COMBI-db/db and MET-db/db. Figure S2. Sensitivity analyses using propensity score matching (PSM).

## Data Availability

KORA data sets are not publicly available because of data protection agreements; however can be provided upon request through the KORA-PASST (Project application self-service tool, https://helmholtz-muenchen.managed-otrs.com/external). The Mouse200 project data sets generated and/or analyzed during the current study are available from the corresponding author upon reasonable request.

## Abbreviations

ADMA: Asymmetric dimethyl arginine
ALT: Alanine aminotransferase
AST: Aspartate aminotransferase
HbA_1C_: Hemoglobin A_1C_
BCAAs: Branched chain amino acids
BMI: Body mass index
COMBI: Combination of Sodium glucose co-transporter 2 inhibitor and metformin
CV: Coefficient of variation
C2: Acetylcarnitine
C3-DC: Malonylcarnitine
C4-OH: Hydroxybutyrylcarnitine
DCA: Deoxycholic acid
eGFR: Estimated glomerular filtration rate
HDL-C: High-density lipoprotein cholesterol
HFD: High-fat diet
HMDB: The Human Metabolome Database (HMDB)
hs-CRP: High-sensitivity C-reactive protein
KEGG: The Kyoto Encyclopedia of Genes and Genomes
KORA: The Cooperative Health Research in the Region of Augsburg
KORA-PASST: The Cooperative Health Research in the Region of Augsburg-Project application self-service tool
LDL-C: Low-density lipoprotein cholesterol
LOD: Limit of detection
MASLD: Metabolic dysfunction-associated steatotic liver disease
MET: Metformin
NAD: Nicotinamide adenine dinucleotide
NADH: Nicotinamide adenine dinucleotide, reduced form
ndt-T2D: Non-glucose-lowering drug-treated type 2 diabetes
NGT: Normal glucose tolerance
NIST: The National Institute of Standards and Technology
NH₃: Ammonia
NLRP3: Nod-like receptor family pyrin domain containing 3
NO: Nitric oxide
PBS: Phosphate buffer saline
RSD: Relative standard deviation
QC: Quality control
SD: Standard deviation
SGLT2i: Sodium glucose co-transporter 2 inhibitor
Slc38a2: Solute carrier family 38 member 2
TDH: Threonine dehydrogenase
TG: Triglycerides
T2D: Type 2 diabetes
VG: Vehicle-gaveged
WT: Wild type
