# Supplementary material for: Urea cycle modulation by combined SGLT2 inhibitors and metformin
Source: BMC Med. 2026 Jan 8;24:55. doi: 10.1186/s12916-025-04609-7 (PMC12849117; doi:10.1186/s12916-025-04609-7)
Supplement: Supplementary file 1 — Additional file 1. Table S1. Quality control results for 620 metabolites in KORA-Fit. Table S2. Characteristics of the four mouse groups. Table S3. List of metabolites utilized in human serum and seven murine tissues. Table S4. Ten significant serum metabolites in COMBI vs. MET-T2D. Table S5. 82 significant plasma metabolites in COMBI vs. MET-db/db. Table S6. 52 significant liver metabolites in COMBI vs. MET-db/db. Table S7. 30 significant adrenal gland metabolites in COMBI vs. MET-db/db. Table S8. 12 significant adipose tissue metabolites in COMBI vs. MET-db/db. Table S9. Seven significant testis metabolites in COMBI vs. MET-db/db. Table S10. Seven significant lung metabolites in COMBI vs. MET-db/db. Table S11. Six significant cerebellum metabolites in COMBI vs. MET-db/db. Table S12. Threonine metabolism-associated liver transcripts in COMBI vs. MET-db/db. Table S13. Group comparisons between COMBI-T2D (N = 25) and MET-T2D (N = 138): effect sizes, confidence intervals, and post-hoc power. Table S14. Sensitivity analyses of the 10 metabolites in six models. Table S15. Correlation between HbA1C and 10 metabolites in T2D patients treated with COMBI or MET. Figure S1. Comparisons of the liver transcripts between COMBI-db/db and MET-db/db. Figure S2. Sensitivity analyses using propensity score matching (PSM). [file 12916_2025_4609_MOESM1_ESM.docx]

# Urea Cycle Modulation by Combined SGLT2 Inhibitors and Metformin

[Additional file 1: 2](#_Toc216337847)

[Table S1. Quality control results for 620 metabolites in KORA-Fit 2](#_Toc216337848)

[Table S2. Characteristics of the four mouse groups 10](#_Toc216337849)

[Table S3. List of metabolites utilized in human serum and seven murine tissues 11](#_Toc216337850)

[Table S4. Ten significant serum metabolites in COMBI vs. MET-T2D 17](#_Toc216337851)

[Table S5. 82 significant plasma metabolites in COMBI vs. MET-db/db 18](#_Toc216337852)

[Table S6. 52 significant liver metabolites in COMBI vs. MET-db/db 20](#_Toc216337853)

[Table S7. 30 significant adrenal gland metabolites in COMBI vs. MET-db/db 21](#_Toc216337854)

[Table S8. 12 significant adipose tissue metabolites in COMBI vs. MET-db/db 22](#_Toc216337855)

[Table S9. Seven significant testis metabolites in COMBI vs. MET-db/db 22](#_Toc216337856)

[Table S10. Seven significant lung metabolites in COMBI vs. MET-db/db 23](#_Toc216337857)

[Table S11. Six significant cerebellum metabolites in COMBI vs. MET-db/db 23](#_Toc216337858)

[Table S12. Threonine metabolism-associated hepatic transcripts in COMBI-db/db mice compared with control groups 23](#_Toc216337859)

[Table S13. Group comparisons between COMBI-T2D (N = 25) and MET-T2D (N = 138): effect sizes, confidence intervals, and post-hoc power 24](#_Toc216337860)

[Table S14. Sensitivity analyses of the 10 metabolites in six models 24](#_Toc216337861)

[Table S15. Correlation between HbA_1C_ and 10 metabolites in T2D patients treated with COMBI or MET 25](#_Toc216337862)

[Figure S1. Comparisons of the liver transcripts between COMBI-db/db and MET-db/db 26](#_Toc216337863)

[Figure S2. Sensitivity analyses using propensity score matching (PSM) 26](#_Toc216337864)

##

## Additional file 1:

## Table S1. Quality control results for 620 metabolites in KORA-Fit

| Metabolite  abbreviation | Biochemical name | % above LOD | Median RSD (%) | % Missing | Application |
| --- | --- | --- | --- | --- | --- |
| C0 | Carnitine | **100.00** | **11.03** | **0.00** | **Used** |
| C2 | Acetylcarnitine | **100.00** | **7.36** | **0.00** | **Used** |
| C3 | Propionylcarnitine | **99.83** | **11.87** | **0.00** | **Used** |
| C3-DC (C4-OH) | Malonylcarnitine (Hydroxybutyrylcarnitine) | **52.20** | 34.13 | **1.59** | Excluded |
| C3-OH | Hydroxypropionylcarnitine | 8.49 | 60.91 | 15.87 | Excluded |
| C3:1 | Propenoylcarnitine | 22.12 | 69.18 | 15.56 | Excluded |
| C4 | Butyrylcarnitine | **96.31** | **10.14** | **0.00** | **Used** |
| C4:1 | Butenylcarnitine | 43.84 | 50.92 | 11.71 | Excluded |
| C5 | Valerylcarnitine | **80.38** | **18.06** | **0.14** | **Used** |
| C5-DC (C6-OH) | Glutarylcarnitine (Hydroxyhexanoylcarnitine) | 6.80 | 49.35 | **1.62** | Excluded |
| C5-M-DC | Methylglutarylcarnitine | 0.85 | 30.80 | **0.47** | Excluded |
| C5-OH (C3-DC-M) | Hydroxyvalerylcarnitine (Methylmalonylcarnitine) | 20.60 | 68.00 | 12.62 | Excluded |
| C5:1 | Tiglylcarnitine | 6.39 | 81.17 | 47.02 | Excluded |
| C5:1-DC | Glutaconylcarnitine | 6.02 | 53.58 | **7.44** | Excluded |
| C6 (C4:1-DC) | Hexanoylcarnitine (Fumarylcarnitine) | 41.98 | 38.23 | **0.03** | Excluded |
| C6:1 | Hexenoylcarnitine | 7.31 | 47.97 | **1.59** | Excluded |
| C7-DC | Pimeloylcarnitine | 4.77 | 79.70 | **0.00** | Excluded |
| C8 | Octanoylcarnitine | 23.92 | **19.68** | **0.00** | Excluded |
| C9 | Nonaylcarnitine | 39.85 | 45.01 | **0.00** | Excluded |
| C10 | Decanoylcarnitine | **89.58** | **21.17** | **0.00** | **Used** |
| C10:1 | Decenoylcarnitine | 1.32 | **20.74** | **0.00** | Excluded |
| C10:2 | Decadienoylcarnitine | 15.46 | 71.05 | **0.00** | Excluded |
| C12 | Dodecanoylcarnitine | **56.09** | **21.57** | **0.00** | **Used** |
| C12-DC | Dodecanedioylcarnitine | 0.00 | 28.14 | **0.00** | Excluded |
| C12:1 | Dodecenoylcarnitine | 31.87 | **21.68** | **0.00** | Excluded |
| C14 | Tetradecanoylcarnitine | 7.85 | **22.11** | **0.00** | Excluded |
| C14:1 | Tetradecenoylcarnitine | **88.60** | **19.71** | **0.00** | **Used** |
| C14:1-OH | Hydroxytetradecenoylcarnitine | 6.09 | 26.60 | **2.33** | Excluded |
| C14:2 | Tetradecadienoylcarnitine | 22.12 | **20.96** | **0.98** | Excluded |
| C14:2-OH | Hydroxytetradecadienoylcarnitine | 2.17 | 29.89 | **0.03** | Excluded |
| C16 | Hexadecanoylcarnitine | **93.03** | **11.45** | **0.00** | **Used** |
| C16-OH | Hydroxyhexadecenoylcarnitine | 1.83 | 31.49 | **0.24** | Excluded |
| C16:1 | Hexadecenoylcarnitine | 7.71 | 28.51 | **0.00** | Excluded |
| C16:1-OH | Hydroxyhexadecenoylcarnitine | 1.89 | **23.24** | **0.14** | Excluded |
| C16:2 | Hexadecadienoylcarnitine | 0.88 | 26.84 | **0.03** | Excluded |
| C16:2-OH | Hydroxyhexadecadienoylcarnitine | 0.58 | 30.61 | **0.17** | Excluded |
| C18 | Octadecanoylcarnitine | **78.89** | **14.47** | **0.00** | **Used** |
| C18:1 | Octadecenoylcarnitine | **82.24** | **14.19** | **0.00** | **Used** |
| C18:1-OH | Hydroxyoctadecenoylcarnitine | 0.07 | 34.46 | **0.00** | Excluded |
| C18:2 | Octadecadienylcarnitine | **54.23** | **13.56** | **0.00** | **Used** |
| Trigonelline | Trigonelline | **84.30** | 97.62 | **0.03** | Excluded |
| TMAO | Trimethylamine N-oxide | **100.00** | 34.24 | **0.00** | Excluded |
| Ala | Alanine | **100.00** | **8.78** | **0.00** | **Used** |
| Arg | Arginine | **100.00** | **11.04** | **0.00** | **Used** |
| Asn | Asparagine | **100.00** | **9.77** | **0.00** | **Used** |
| Asp | Aspartate | **76.86** | **14.05** | **2.47** | **Used** |
| Cys | Cysteine | **100.00** | 26.43 | **0.00** | Excluded |
| Gln | Glutamine | **100.00** | **10.56** | **0.00** | **Used** |
| Glu | Glutamate | **100.00** | **10.16** | **0.00** | **Used** |
| Gly | Glycine | **100.00** | **9.14** | **0.00** | **Used** |
| His | Histidine | **100.00** | **9.11** | **0.00** | **Used** |
| Ile | Isoleucine | **100.00** | **9.41** | **0.00** | **Used** |
| Leu | Leucine | **100.00** | **9.60** | **0.00** | **Used** |
| Lys | Lysine | **100.00** | **11.04** | **0.00** | **Used** |
| Met | Methionine | **100.00** | **11.02** | **0.00** | **Used** |
| Phe | Phenylalanine | **100.00** | **9.62** | **0.00** | **Used** |
| Pro | Proline | **100.00** | **9.44** | **0.00** | **Used** |
| Ser | Serine | **100.00** | **9.93** | **0.00** | **Used** |
| Thr | Threonine | **100.00** | **10.25** | **0.00** | **Used** |
| Trp | Tryptophan | **100.00** | **10.62** | **0.00** | **Used** |
| Tyr | Tyrosine | **100.00** | **10.51** | **0.00** | **Used** |
| Val | Valine | **100.00** | **9.14** | **0.00** | **Used** |
| 1-Met-His | 1-Methylhistidine | **100.00** | **6.54** | **0.00** | **Used** |
| 3-Met-His | 3-Methylhistidine | **100.00** | **10.80** | **0.00** | **Used** |
| 5-AVA | 5-Aminovaleric acid | 49.97 | **11.63** | **0.00** | Excluded |
| AABA | α-Aminobutyric acid | **100.00** | **14.99** | **0.00** | **Used** |
| Ac-Orn | Acetylornithine | 0.07 | 85.71 | 95.53 | Excluded |
| ADMA | Asymmetric dimethylarginine | **100.00** | **9.89** | **0.00** | **Used** |
| alpha-AAA | α-Aminoadipic acid | **94.59** | **16.22** | **0.07** | **Used** |
| Anserine | Anserine | 17.35 | **17.59** | **4.36** | Excluded |
| BABA | β-Aminobutyric acid | **75.00** | 34.79 | **0.14** | Excluded |
| Betaine | Betaine | **99.97** | 25.48 | **0.00** | Excluded |
| c4-OH-Pro | cis -4-Hydroxyproline | 0.03 | 89.74 | 76.12 | Excluded |
| Carnosine | Carnosine | 0.30 | 51.94 | 55.31 | Excluded |
| Cit | Citrulline | **100.00** | **10.52** | **0.00** | **Used** |
| Creatinine | Creatinine | **100.00** | **9.32** | **0.00** | **Used** |
| Cystine | Cystine | 100.00 | 61.96 | **0.00** | Excluded |
| DOPA | Dihydroxyphenylalanine | 3.76 | 62.56 | 35.83 | Excluded |
| HArg | Homoarginine | **100.00** | **19.86** | **0.00** | **Used** |
| HCys | Homocysteine | **100.00** | 36.81 | **0.00** | Excluded |
| Kynurenine | Kynurenine | **99.97** | **11.86** | **0.00** | **Used** |
| Met-SO | Methionine sulfoxide | **96.11** | **15.30** | **0.88** | **Used** |
| Nitro-Tyr | Nitrotyrosine | 2.37 | 69.35 | 63.26 | Excluded |
| Orn | Ornithine | **100.00** | **10.28** | **0.00** | **Used** |
| PAG | Phenylacetylglycine | 17.02 | 52.49 | 16.58 | Excluded |
| PheAlaBetaine | Phenylalanine betaine | 17.35 | 23.86 | **0.00** | Excluded |
| ProBetaine | Proline betaine | **100.00** | **21.79** | **0.00** | **Used** |
| Sarcosine | Sarcosine | **99.90** | **12.76** | **0.03** | **Used** |
| SDMA | Symmetric dimethylarginine | **100.00** | **11.81** | **0.00** | **Used** |
| t4-OH-Pro | trans -4-Hydroxyproline | **100.00** | **9.65** | **0.00** | **Used** |
| Taurine | Taurine | **100.00** | **9.50** | **0.00** | **Used** |
| TrpBetaine | Tryptophan betaine | **98.44** | **15.70** | **0.00** | **Used** |
| CA | Cholic acid | **68.50** | **12.00** | **2.54** | **Used** |
| CDCA | Chenodeoxycholic acid | **55.35** | **17.98** | **3.21** | **Used** |
| DCA | Deoxycholic acid | **98.34** | **11.79** | **0.03** | **Used** |
| GCA | Glycocholic acid | **99.46** | **8.71** | **0.00** | **Used** |
| GCDCA | Glycochenodeoxycholic acid | **100.00** | **19.30** | **0.00** | **Used** |
| GDCA | Glycodeoxycholic acid | **98.88** | **14.28** | **0.61** | **Used** |
| GLCA | Glycolithocholic acid | **75.14** | 27.65 | **0.00** | Excluded |
| GLCAS | Glycolithocholic acid sulfate | **99.59** | 28.82 | **0.00** | Excluded |
| GUDCA | Glycoursodeoxycholic acid | **99.32** | **10.00** | **0.07** | **Used** |
| TCA | Taurocholic acid | **78.42** | **10.41** | **0.00** | **Used** |
| TCDCA | Taurochenodeoxycholic acid | **91.81** | **9.15** | **0.00** | **Used** |
| TDCA | Taurodeoxycholic acid | **85.01** | **22.63** | **0.41** | **Used** |
| TMCA | Tauromurocholic acid | 29.13 | 37.27 | 50.91 | Excluded |
| beta-Ala | β-Alanine | **100.00** | **12.28** | **0.00** | **Used** |
| Dopamine | Dopamine | 1.93 | 94.24 | 55.21 | Excluded |
| GABA | γ-Aminobutyric acid | **95.57** | **13.61** | **0.00** | **Used** |
| Histamine | Histamine | 0.20 | 61.76 | 59.84 | Excluded |
| PEA | Phenylethylamine | 0.00 | **1.93** | 97.46 | Excluded |
| Putrescine | Putrescine | **76.73** | 25.44 | **0.00** | Excluded |
| Serotonin | Serotonin | **97.29** | 37.74 | **0.41** | Excluded |
| Spermidine | Spermidine | 15.66 | 26.60 | **0.00** | Excluded |
| Spermine | Spermine | 1.42 | 39.41 | 36.81 | Excluded |
| AconAcid | Aconitic acid | **99.93** | 33.51 | **0.00** | Excluded |
| DiCA(12:0) | Dodecanedioic acid | 2.74 | 46.14 | **0.00** | Excluded |
| DiCA(14:0) | Tetradecanedioic acid | 13.84 | 42.70 | **0.00** | Excluded |
| HipAcid | Hippuric acid | **99.39** | 26.77 | **0.00** | Excluded |
| Lac | Lactic acid | **100.00** | **8.56** | **0.00** | **Used** |
| OH-GlutAcid | 3-Hydroxyglutaric acid | 6.43 | 30.40 | **0.00** | Excluded |
| Suc | Succinic acid | 0.00 | 23.34 | **0.00** | Excluded |
| Cer (d16:1/18:0) | Ceramide d16:1/18:0 | **86.84** | 35.93 | **0.00** | Excluded |
| Cer (d16:1/20:0) | Ceramide d16:1/20:0 | **98.88** | 34.08 | **0.00** | Excluded |
| Cer (d16:1/22:0) | Ceramide d16:1/22:0 | **100.00** | 36.12 | **0.00** | Excluded |
| Cer (d16:1/23:0) | Ceramide d16:1/23:0 | **99.97** | 41.64 | **0.00** | Excluded |
| Cer (d16:1/24:0) | Ceramide d16:1/24:0 | **100.00** | 43.52 | **0.00** | Excluded |
| Cer (d18:1/14:0) | Ceramide d18:1/14:0 | **66.91** | 35.05 | **0.00** | Excluded |
| Cer (d18:1/16:0) | Ceramide d18:1/16:0 | **100.00** | 25.19 | **0.00** | Excluded |
| Cer (d18:1/18:0(OH)) | Ceramide d18:1/18:0(OH) | 9.81 | 44.16 | **0.03** | Excluded |
| Cer (d18:1/18:0) | Ceramide d18:1/18:0 | **100.00** | 29.87 | **0.00** | Excluded |
| Cer (d18:1/18:1) | Ceramide d18:1/18:1 | 39.11 | 29.87 | **0.00** | Excluded |
| Cer (d18:1/20:0(OH)) | Ceramide d18:1/20:0(OH) | 17.22 | 145.74 | **0.00** | Excluded |
| Cer (d18:1/20:0) | Ceramide d18:1/20:0 | **99.90** | 33.67 | **0.00** | Excluded |
| Cer (d18:1/22:0) | Ceramide d18:1/22:0 | **100.00** | 46.43 | **0.00** | Excluded |
| Cer (d18:1/23:0) | Ceramide d18:1/23:0 | **100.00** | 47.12 | **0.00** | Excluded |
| Cer (d18:1/24:0) | Ceramide d18:1/24:0 | **100.00** | 54.58 | **0.00** | Excluded |
| Cer (d18:1/24:1) | Ceramide d18:1/24:0 | **100.00** | 49.57 | **0.00** | Excluded |
| Cer (d18:1/25:0) | Ceramide d18:1/25:0 | **97.70** | 39.82 | **0.00** | Excluded |
| Cer (d18:1/26:0) | Ceramide d18:1/26:0 | **96.08** | 59.29 | **0.00** | Excluded |
| Cer (d18:1/26:1) | Ceramide d18:1/26:1 | **96.11** | 57.41 | **0.00** | Excluded |
| Cer (d18:2/14:0) | Ceramide d18:2/14:0 | 41.07 | 51.19 | **0.27** | Excluded |
| Cer (d18:2/16:0) | Ceramide d18:2/16:0 | **99.83** | 29.79 | **0.00** | Excluded |
| Cer (d18:2/18:0) | Ceramide d18:2/18:0 | **99.63** | 33.67 | **0.00** | Excluded |
| Cer (d18:2/18:1) | Ceramide d18:2/18:1 | 29.91 | 48.14 | **0.24** | Excluded |
| Cer (d18:2/20:0) | Ceramide d18:2/20:0 | **99.90** | 41.38 | **0.00** | Excluded |
| Cer (d18:2/22:0) | Ceramide d18:2/22:0 | **100.00** | 38.15 | **0.00** | Excluded |
| Cer (d18:2/23:0) | Ceramide d18:2/23:0 | **99.97** | 41.36 | **0.00** | Excluded |
| Cer (d18:2/24:0) | Ceramide d18:2/24:0 | **100.00** | 48.13 | **0.00** | Excluded |
| Cer (d18:2/24:1) | Ceramide d18:2/24:1 | **100.00** | 41.94 | **0.00** | Excluded |
| CE (14:0) | Cholesteryl ester 14:0 | **99.97** | 26.49 | **0.03** | Excluded |
| CE (14:1) | Cholesteryl ester 14:1 | **99.42** | 35.06 | **0.00** | Excluded |
| CE (15:0) | Cholesteryl ester 15:0 | **99.83** | 30.85 | **0.03** | Excluded |
| CE (15:1) | Cholesteryl ester 15:1 | 22.33 | 42.56 | **0.03** | Excluded |
| CE (16:0) | Cholesteryl ester 16:0 | **99.93** | 33.07 | **0.03** | Excluded |
| CE (16:1) | Cholesteryl ester 16:1 | **100.00** | 28.33 | **0.00** | Excluded |
| CE (17:0) | Cholesteryl ester 17:0 | **99.83** | 25.94 | **0.00** | Excluded |
| CE (17:1) | Cholesteryl ester 17:1 | **99.97** | 29.03 | **0.00** | Excluded |
| CE (18:0) | Cholesteryl ester 18:0 | **99.97** | 27.72 | **0.00** | Excluded |
| CE (18:1) | Cholesteryl ester 18:1 | **100.00** | 27.38 | **0.00** | Excluded |
| CE (18:2) | Cholesteryl ester 18:2 | **100.00** | **21.83** | **0.00** | **Used** |
| CE (18:3) | Cholesteryl ester 18:3 | **100.00** | **20.60** | **0.00** | **Used** |
| CE (20:0) | Cholesteryl ester 20:0 | **69.96** | **19.86** | **1.59** | **Used** |
| CE (20:1) | Cholesteryl ester 20:1 | **85.08** | **21.50** | **0.41** | **Used** |
| CE (20:3) | Cholesteryl ester 20:3 | **100.00** | **23.55** | **0.00** | **Used** |
| CE (20:4) | Cholesteryl ester 20:4 | **100.00** | **20.84** | **0.00** | **Used** |
| CE (20:5) | Cholesteryl ester 20:5 | **100.00** | **19.77** | **0.00** | **Used** |
| CE (22:0) | Cholesteryl ester 22:0 | 24.42 | 34.31 | **0.34** | Excluded |
| CE (22:1) | Cholesteryl ester 22:1 | 27.23 | 68.81 | **0.37** | Excluded |
| CE (22:2) | Cholesteryl ester 22:2 | **59.20** | **17.37** | **0.17** | **Used** |
| CE (22:5) | Cholesteryl ester 22:5 | **99.97** | **20.83** | **0.00** | **Used** |
| CE (22:6) | Cholesteryl ester 22:6 | **100.00** | **20.67** | **0.00** | **Used** |
| p-Cresol-SO4 | p-Cresol sulfate | **100.00** | 28.23 | **0.00** | Excluded |
| DG(14:0_14:0) | Diglyceride 14:0_14:0 | 18.81 | 80.29 | **0.00** | Excluded |
| DG(14:0_18:1) | Diglyceride 14:0_18:1 | 42.69 | 36.02 | **0.00** | Excluded |
| DG(14:0_18:2) | Diglyceride 14:0_18:2 | 31.26 | 29.23 | **0.00** | Excluded |
| DG(14:0_20:0) | Diglyceride 14:0_20:0 | 2.94 | 107.74 | **0.00** | Excluded |
| DG(14:1_18:1) | Diglyceride 14:1_18:1 | **53.59** | 30.70 | **0.07** | Excluded |
| DG(14:1_20:2) | Diglyceride 14:1_20:2 | 0.71 | 47.83 | **0.74** | Excluded |
| DG(16:0_16:0) | Diglyceride 16:0_16:0 | 0.00 | **18.87** | **0.00** | Excluded |
| DG(16:0_16:1) | Diglyceride 16:0_16:1 | **65.70** | **24.07** | **0.17** | **Used** |
| DG(16:0_18:1) | Diglyceride 16:0_18:1 | **83.42** | **22.86** | **0.00** | **Used** |
| DG(16:0_18:2) | Diglyceride 16:0_18:2 | **99.42** | **18.29** | **0.00** | **Used** |
| DG(16:0_20:0) | Diglyceride 16:0_20:0 | 4.53 | **22.31** | **0.00** | Excluded |
| DG(16:0_20:3) | Diglyceride 16:0_20:3 | 19.55 | 31.99 | **0.24** | Excluded |
| DG(16:0_20:4) | Diglyceride 16:0_20:4 | 7.78 | 63.95 | **0.07** | Excluded |
| DG(16:1_18:0) | Diglyceride 16:1_18:0 | 30.72 | 26.50 | **0.07** | Excluded |
| DG(16:1_18:1) | Diglyceride 16:1_18:1 | 27.77 | 92.36 | **0.00** | Excluded |
| DG(16:1_18:2) | Diglyceride 16:1_18:2 | **94.89** | **20.09** | **0.00** | **Used** |
| DG(16:1_20:0) | Diglyceride 16:1_20:0 | 3.89 | 41.05 | **0.30** | Excluded |
| DG(17:0_17:1) | Diglyceride 17:0_17:1 | 2.44 | 65.22 | **0.20** | Excluded |
| DG(17:0_18:1) | Diglyceride 17:0_18:1 | **91.27** | **20.06** | **0.00** | **Used** |
| DG(18:0_20:0) | Diglyceride 18:0_20:0 | 1.08 | 134.82 | **0.00** | Excluded |
| DG(18:0_20:4) | Diglyceride 18:0_20:4 | 13.19 | 49.13 | **0.24** | Excluded |
| DG(18:1_18:1) | Diglyceride 18:1_18:1 | **100.00** | **19.88** | **0.00** | **Used** |
| DG(18:1_18:2) | Diglyceride 18:1_18:2 | **100.00** | **19.55** | **0.00** | **Used** |
| DG(18:1_18:3) | Diglyceride 18:1_18:3 | **79.13** | **20.95** | **0.00** | **Used** |
| DG(18:1_18:4) | Diglyceride 18:1_18:4 | 4.47 | 40.87 | **0.37** | Excluded |
| DG(18:1_20:0) | Diglyceride 18:1_20:0 | **71.31** | **24.77** | **0.00** | **Used** |
| DG(18:1_20:1) | Diglyceride 18:1_20:1 | **98.99** | **24.24** | **0.00** | **Used** |
| DG(18:1_20:2) | Diglyceride 18:1_20:2 | 28.55 | 27.60 | **0.00** | Excluded |
| DG(18:1_20:3) | Diglyceride 18:1_20:3 | **59.64** | 27.45 | **0.00** | Excluded |
| DG(18:1_20:4) | Diglyceride 18:1_20:4 | **54.53** | 27.79 | **0.00** | Excluded |
| DG(18:1_22:5) | Diglyceride 18:1_22:5 | 10.49 | 76.31 | **0.00** | Excluded |
| DG(18:1_22:6) | Diglyceride 18:1_22:6 | 31.63 | 78.78 | **0.24** | Excluded |
| DG(18:2_18:2) | Diglyceride 18:2_18:2 | **99.29** | **18.82** | **0.00** | **Used** |
| DG(18:2_18:3) | Diglyceride 18:2_18:3 | **57.85** | 27.07 | **0.47** | Excluded |
| DG(18:2_18:4) | Diglyceride 18:2_18:4 | 20.03 | 44.76 | **0.34** | Excluded |
| DG(18:2_20:0) | Diglyceride 18:2_20:0 | 25.37 | 33.18 | **0.03** | Excluded |
| DG(18:2_20:4) | Diglyceride 18:2_20:4 | 35.39 | 27.16 | **0.00** | Excluded |
| DG(18:3_18:3) | Diglyceride 18:3_18:3 | 11.67 | 69.54 | **0.44** | Excluded |
| DG(18:3_20:2) | Diglyceride 18:3_20:2 | 6.02 | 80.93 | **0.47** | Excluded |
| DG(21:0_22:6) | Diglyceride 21:0_22:6 | 8.86 | 53.57 | **1.79** | Excluded |
| DG(22:1_22:2) | Diglyceride 22:1_22:2 | 5.21 | 62.68 | **1.29** | Excluded |
| DG-O(14:0_18:2) | Diglyceride-O 14:0_18:2 | 2.03 | 82.89 | **0.20** | Excluded |
| DG-O(16:0_18:1) | Diglyceride-O 16:0_18:1 | 12.89 | 31.52 | **0.27** | Excluded |
| DG-O(16:0_20:4) | Diglyceride-O 16:0_20:4 | 3.18 | 51.33 | **0.98** | Excluded |
| Cer (d18:0/18:0(OH)) | Dihydroceramide d18:0/18:0(OH) | 3.48 | 53.76 | **0.00** | Excluded |
| Cer (d18:0/18:0) | Dihydroceramide d18:0/18:0 | 21.85 | 29.52 | **1.32** | Excluded |
| Cer (d18:0/20:0) | Dihydroceramide d18:0/20:0 | 23.68 | 39.92 | **0.00** | Excluded |
| Cer (d18:0/22:0) | Dihydroceramide d18:0/22:0 | **59.10** | 41.25 | **0.00** | Excluded |
| Cer (d18:0/24:0) | Dihydroceramide d18:0/24:0 | **99.26** | **19.22** | **0.00** | **Used** |
| Cer (d18:0/24:1) | Dihydroceramide d18:0/24:1 | **96.89** | **23.16** | **0.00** | **Used** |
| Cer (d18:0/26:1(OH)) | Dihydroceramide d18:0/26:1(OH) | 2.64 | 140.02 | **0.00** | Excluded |
| Cer (d18:0/26:1) | Dihydroceramide d18:0/26:1 | 9.20 | 48.58 | **0.61** | Excluded |
| AA | Arachidonic acid | **99.66** | 46.57 | **0.00** | Excluded |
| DHA | Docosahexaenoid acid | **100.00** | 35.42 | **0.00** | Excluded |
| EPA | Eicosapentaenoic acid | **90.49** | 33.18 | **0.14** | Excluded |
| LysoPC a C14:0 | LysoPhosphatidylcholine acyl C14:0 | **99.90** | **10.81** | **0.00** | **Used** |
| LysoPC a C16:0 | LysoPhosphatidylcholine acyl C16:0 | **100.00** | **11.41** | **0.00** | **Used** |
| LysoPC a C16:1 | LysoPhosphatidylcholine acyl C16:1 | **100.00** | **11.40** | **0.00** | **Used** |
| LysoPC a C17:0 | LysoPhosphatidylcholine acyl C17:0 | **100.00** | **11.98** | **0.00** | **Used** |
| LysoPC a C18:0 | LysoPhosphatidylcholine acyl C18:0 | **100.00** | **13.58** | **0.00** | **Used** |
| LysoPC a C18:1 | LysoPhosphatidylcholine acyl C18:1 | **100.00** | **12.47** | **0.00** | **Used** |
| LysoPC a C18:2 | LysoPhosphatidylcholine acyl C18:2 | **100.00** | **12.69** | **0.00** | **Used** |
| LysoPC a C20:3 | LysoPhosphatidylcholine acyl C20:3 | **100.00** | **14.39** | **0.00** | **Used** |
| LysoPC a C20:4 | LysoPhosphatidylcholine acyl C20:4 | **100.00** | **13.15** | **0.00** | **Used** |
| LysoPC a C24:0 | LysoPhosphatidylcholine acyl C24:0 | **99.59** | **23.80** | **0.00** | **Used** |
| LysoPC a C26:0 | LysoPhosphatidylcholine acyl C26:0 | **98.44** | 49.02 | **0.00** | Excluded |
| LysoPC a C26:1 | LysoPhosphatidylcholine acyl C26:1 | **98.11** | 48.91 | **0.00** | Excluded |
| LysoPC a C28:0 | LysoPhosphatidylcholine acyl C28:0 | **94.05** | 36.55 | **0.00** | Excluded |
| LysoPC a C28:1 | LysoPhosphatidylcholine acyl C28:1 | **100.00** | 31.13 | **0.00** | Excluded |
| PC aa C24:0 | Phosphatidylcholine diacyl C24:0 | **68.50** | 42.14 | **0.00** | Excluded |
| PC aa C26:0 | Phosphatidylcholine diacyl C26:0 | 16.14 | 40.10 | **0.00** | Excluded |
| PC aa C28:1 | Phosphatidylcholine diacyl C28:1 | **100.00** | **7.75** | **0.00** | **Used** |
| PC aa C30:0 | Phosphatidylcholine diacyl C30:0 | **100.00** | **10.74** | **0.00** | **Used** |
| PC aa C30:2 | Phosphatidylcholine diacyl C30:2 | 16.20 | 78.31 | 83.86 | Excluded |
| PC aa C32:0 | Phosphatidylcholine diacyl C32:0 | **100.00** | 44.67 | **0.00** | Excluded |
| PC aa C32:1 | Phosphatidylcholine diacyl C32:1 | **100.00** | 46.77 | **0.00** | Excluded |
| PC aa C32:2 | Phosphatidylcholine diacyl C32:2 | **99.97** | 51.58 | **0.03** | Excluded |
| PC aa C32:3 | Phosphatidylcholine diacyl C32:3 | **100.00** | 50.76 | **0.00** | Excluded |
| PC aa C34:1 | Phosphatidylcholine diacyl C34:1 | **100.00** | 41.05 | **0.00** | Excluded |
| PC aa C34:2 | Phosphatidylcholine diacyl C34:2 | **100.00** | 46.54 | **0.00** | Excluded |
| PC aa C34:3 | Phosphatidylcholine diacyl C34:3 | **100.00** | 43.69 | **0.00** | Excluded |
| PC aa C34:4 | Phosphatidylcholine diacyl C34:4 | **100.00** | 46.36 | **0.00** | Excluded |
| PC aa C36:0 | Phosphatidylcholine diacyl C36:0 | **99.86** | 48.05 | **0.03** | Excluded |
| PC aa C36:1 | Phosphatidylcholine diacyl C36:1 | **100.00** | 29.43 | **0.00** | Excluded |
| PC aa C36:2 | Phosphatidylcholine diacyl C36:2 | **100.00** | 36.58 | **0.00** | Excluded |
| PC aa C36:3 | Phosphatidylcholine diacyl C36:3 | **100.00** | 37.34 | **0.00** | Excluded |
| PC aa C36:4 | Phosphatidylcholine diacyl C36:4 | **100.00** | 39.49 | **0.00** | Excluded |
| PC aa C36:5 | Phosphatidylcholine diacyl C36:5 | **100.00** | 38.62 | **0.00** | Excluded |
| PC aa C36:6 | Phosphatidylcholine diacyl C36:6 | **100.00** | 40.93 | **0.00** | Excluded |
| PC aa C38:0 | Phosphatidylcholine diacyl C38:0 | **100.00** | 28.08 | **0.00** | Excluded |
| PC aa C38:1 | Phosphatidylcholine diacyl C38:1 | **97.12** | 70.50 | **2.23** | Excluded |
| PC aa C38:3 | Phosphatidylcholine diacyl C38:3 | **100.00** | 26.22 | **0.00** | Excluded |
| PC aa C38:4 | Phosphatidylcholine diacyl C38:4 | **100.00** | 29.47 | **0.00** | Excluded |
| PC aa C38:5 | Phosphatidylcholine diacyl C38:5 | **100.00** | 29.98 | **0.00** | Excluded |
| PC aa C38:6 | Phosphatidylcholine diacyl C38:6 | **100.00** | 32.79 | **0.00** | Excluded |
| PC aa C40:1 | Phosphatidylcholine diacyl C40:1 | 11.33 | **19.30** | **0.00** | Excluded |
| PC aa C40:2 | Phosphatidylcholine diacyl C40:2 | **100.00** | **24.82** | **0.00** | **Used** |
| PC aa C40:3 | Phosphatidylcholine diacyl C40:3 | **100.00** | 26.30 | **0.00** | Excluded |
| PC aa C40:4 | Phosphatidylcholine diacyl C40:4 | **100.00** | **17.78** | **0.00** | **Used** |
| PC aa C40:5 | Phosphatidylcholine diacyl C40:5 | **100.00** | **20.71** | **0.00** | **Used** |
| PC aa C40:6 | Phosphatidylcholine diacyl C40:6 | **100.00** | **20.99** | **0.00** | **Used** |
| PC aa C42:0 | Phosphatidylcholine diacyl C42:0 | **100.00** | **10.77** | **0.00** | **Used** |
| PC aa C42:1 | Phosphatidylcholine diacyl C42:1 | **100.00** | **14.79** | **0.00** | **Used** |
| PC aa C42:2 | Phosphatidylcholine diacyl C42:2 | **99.76** | **20.40** | **0.00** | **Used** |
| PC aa C42:4 | Phosphatidylcholine diacyl C42:4 | **100.00** | **13.53** | **0.00** | **Used** |
| PC aa C42:5 | Phosphatidylcholine diacyl C42:5 | **100.00** | **12.52** | **0.00** | **Used** |
| PC aa C42:6 | Phosphatidylcholine diacyl C42:6 | **99.86** | **16.34** | **0.00** | **Used** |
| PC ae C30:0 | Phosphatidylcholine acyl-alkyl C30:0 | **100.00** | **14.86** | **0.00** | **Used** |
| PC ae C30:1 | Phosphatidylcholine acyl-alkyl C30:1 | **64.61** | 78.20 | 32.27 | Excluded |
| PC ae C30:2 | Phosphatidylcholine acyl-alkyl C30:2 | **100.00** | **23.87** | **0.00** | **Used** |
| PC ae C32:1 | Phosphatidylcholine acyl-alkyl C32:1 | **100.00** | 47.38 | **0.00** | Excluded |
| PC ae C32:2 | Phosphatidylcholine acyl-alkyl C32:2 | **100.00** | 51.98 | **0.00** | Excluded |
| PC ae C34:0 | Phosphatidylcholine acyl-alkyl C34:0 | **100.00** | 41.73 | **0.00** | Excluded |
| PC ae C34:1 | Phosphatidylcholine acyl-alkyl C34:1 | **100.00** | 41.69 | **0.00** | Excluded |
| PC ae C34:2 | Phosphatidylcholine acyl-alkyl C34:2 | **100.00** | 44.26 | **0.00** | Excluded |
| PC ae C34:3 | Phosphatidylcholine acyl-alkyl C34:3 | **100.00** | 46.11 | **0.00** | Excluded |
| PC ae C36:0 | Phosphatidylcholine acyl-alkyl C36:0 | **100.00** | 40.89 | **0.00** | Excluded |
| PC ae C36:1 | Phosphatidylcholine acyl-alkyl C36:1 | **100.00** | 39.21 | **0.00** | Excluded |
| PC ae C36:2 | Phosphatidylcholine acyl-alkyl C36:2 | **100.00** | 38.13 | **0.00** | Excluded |
| PC ae C36:3 | Phosphatidylcholine acyl-alkyl C36:3 | **100.00** | 38.94 | **0.00** | Excluded |
| PC ae C36:4 | Phosphatidylcholine acyl-alkyl C36:4 | **100.00** | 38.82 | **0.00** | Excluded |
| PC ae C36:5 | Phosphatidylcholine acyl-alkyl C36:5 | **100.00** | 40.49 | **0.00** | Excluded |
| PC ae C38:0 | Phosphatidylcholine acyl-alkyl C38:0 | **100.00** | 26.00 | **0.00** | Excluded |
| PC ae C38:1 | Phosphatidylcholine acyl-alkyl C38:1 | **94.55** | 37.98 | **3.72** | Excluded |
| PC ae C38:2 | Phosphatidylcholine acyl-alkyl C38:2 | **100.00** | 33.87 | **0.00** | Excluded |
| PC ae C38:3 | Phosphatidylcholine acyl-alkyl C38:3 | **100.00** | 35.78 | **0.00** | Excluded |
| PC ae C38:4 | Phosphatidylcholine acyl-alkyl C38:4 | **100.00** | 31.26 | **0.00** | Excluded |
| PC ae C38:5 | Phosphatidylcholine acyl-alkyl C38:5 | **100.00** | 31.34 | **0.00** | Excluded |
| PC ae C38:6 | Phosphatidylcholine acyl-alkyl C38:6 | **100.00** | 34.14 | **0.00** | Excluded |
| PC ae C40:1 | Phosphatidylcholine acyl-alkyl C40:1 | **100.00** | 28.74 | **0.00** | Excluded |
| PC ae C40:2 | Phosphatidylcholine acyl-alkyl C40:2 | **100.00** | 20.44 | **0.00** | **Used** |
| PC ae C40:3 | Phosphatidylcholine acyl-alkyl C40:3 | **100.00** | 25.28 | **0.00** | Excluded |
| PC ae C40:4 | Phosphatidylcholine acyl-alkyl C40:4 | **100.00** | 25.43 | **0.00** | Excluded |
| PC ae C40:5 | Phosphatidylcholine acyl-alkyl C40:5 | **100.00** | 27.44 | **0.00** | Excluded |
| PC ae C40:6 | Phosphatidylcholine acyl-alkyl C40:6 | **100.00** | 26.15 | **0.00** | Excluded |
| PC ae C42:0 | Phosphatidylcholine acyl-alkyl C42:0 | 44.93 | **11.24** | **0.00** | Excluded |
| PC ae C42:1 | Phosphatidylcholine acyl-alkyl C42:1 | **99.46** | **24.52** | **0.00** | **Used** |
| PC ae C42:2 | Phosphatidylcholine acyl-alkyl C42:2 | **100.00** | **14.77** | **0.00** | **Used** |
| PC ae C42:3 | Phosphatidylcholine acyl-alkyl C42:3 | **100.00** | **22.54** | **0.00** | **Used** |
| PC ae C42:4 | Phosphatidylcholine acyl-alkyl C42:4 | **100.00** | **12.44** | **0.00** | **Used** |
| PC ae C42:5 | Phosphatidylcholine acyl-alkyl C42:5 | **99.93** | **13.02** | **0.00** | **Used** |
| PC ae C44:3 | Phosphatidylcholine acyl-alkyl C44:3 | **100.00** | **24.04** | **0.00** | **Used** |
| PC ae C44:4 | Phosphatidylcholine acyl-alkyl C44:4 | **99.90** | **11.15** | **0.00** | **Used** |
| PC ae C44:5 | Phosphatidylcholine acyl-alkyl C44:5 | **100.00** | **11.55** | **0.00** | **Used** |
| PC ae C44:6 | Phosphatidylcholine acyl-alkyl C44:6 | **100.00** | **10.07** | **0.00** | **Used** |
| Hex2Cer (d18:1/14:0) | Dihexosylceramide d18:1/14:0 | **74.39** | 91.07 | **0.00** | Excluded |
| Hex2Cer (d18:1/16:0) | Dihexosylceramide d18:1/16:0 | **100.00** | **12.25** | **0.00** | **Used** |
| Hex2Cer (d18:1/18:0) | Dihexosylceramide d18:1/18:0 | **100.00** | **17.34** | **0.00** | **Used** |
| Hex2Cer (d18:1/20:0) | Dihexosylceramide d18:1/20:0 | **99.93** | 28.46 | **0.00** | Excluded |
| Hex2Cer (d18:1/22:0) | Dihexosylceramide d18:1/22:0 | **100.00** | 25.08 | **0.00** | Excluded |
| Hex2Cer (d18:1/24:0) | Dihexosylceramide d18:1/24:0 | **100.00** | 32.75 | **0.00** | Excluded |
| Hex2Cer (d18:1/24:1) | Dihexosylceramide d18:1/24:1 | **100.00** | 26.32 | **0.00** | Excluded |
| Hex2Cer (d18:1/26:0) | Dihexosylceramide d18:1/26:0 | **55.35** | 57.67 | **8.29** | Excluded |
| Hex2Cer (d18:1/26:1) | Dihexosylceramide d18:1/26:1 | **61.27** | 72.37 | **0.14** | Excluded |
| Hex3Cer (d18:1/16:0) | Trihexosylceramide d18:1/16:0 | **100.00** | 30.46 | **0.00** | Excluded |
| Hex3Cer (d18:1/18:0) | Trihexosylceramide d18:1/18:0 | **99.53** | 43.82 | **0.00** | Excluded |
| Hex3Cer (d18:1/24:1) | Trihexosylceramide d18:1/24:1 | **99.49** | 46.11 | **0.00** | Excluded |
| Hex3Cer (d18:1/26:1) | Trihexosylceramide d18:1/26:1 | **60.72** | 97.96 | **2.67** | Excluded |
| Hex3Cer (d18:1_20:0) | Trihexosylceramide d18:1_20:0 | **87.52** | 60.58 | **0.03** | Excluded |
| Hex3Cer (d18:1_22:0) | Trihexosylceramide d18:1_22:0 | **99.42** | 50.66 | **0.00** | Excluded |
| HexCer (d16:1/22:0) | Hexosylceramide d16:1/22:0 | **100.00** | **22.43** | **0.00** | **Used** |
| HexCer (d16:1/24:0) | Hexosylceramide d16:1/24:0 | **100.00** | 32.74 | **0.00** | Excluded |
| HexCer (d18:1/14:0) | Hexosylceramide d18:1/14:0 | **79.80** | 31.88 | **0.00** | Excluded |
| HexCer (d18:1/16:0) | Hexosylceramide d18:1/16:0 | **100.00** | **9.25** | **0.00** | **Used** |
| HexCer (d18:1/18:0) | Hexosylceramide d18:1/18:0 | **100.00** | **16.27** | **0.00** | **Used** |
| HexCer (d18:1/18:1) | Hexosylceramide d18:1/18:1 | **98.31** | **24.45** | **0.00** | **Used** |
| HexCer (d18:1/20:0) | Hexosylceramide d18:1/20:0 | **100.00** | **18.02** | **0.00** | **Used** |
| HexCer (d18:1/22:0) | Hexosylceramide d18:1/22:0 | **100.00** | **18.11** | **0.00** | **Used** |
| HexCer (d18:1/23:0) | Hexosylceramide d18:1/23:0 | **100.00** | **19.75** | **0.00** | **Used** |
| HexCer (d18:1/24:0) | Hexosylceramide d18:1/24:0 | **100.00** | **22.24** | **0.00** | **Used** |
| HexCer (d18:1/24:1) | Hexosylceramide d18:1/24:1 | **100.00** | **22.14** | **0.00** | **Used** |
| HexCer (d18:1/26:0) | Hexosylceramide d18:1/26:0 | **95.16** | 43.84 | **0.00** | Excluded |
| HexCer (d18:1/26:1) | Hexosylceramide d18:1/26:1 | **88.87** | 40.76 | **0.00** | Excluded |
| HexCer (d18:2/16:0) | Hexosylceramide d18:2/16:0 | **95.94** | 25.40 | **0.00** | Excluded |
| HexCer (d18:2/18:0) | Hexosylceramide d18:2/18:0 | **96.14** | 28.74 | **0.00** | Excluded |
| HexCer (d18:2/20:0) | Hexosylceramide d18:2/20:0 | **91.68** | 29.36 | **0.00** | Excluded |
| HexCer (d18:2/22:0) | Hexosylceramide d18:2/22:0 | **100.00** | **20.25** | **0.00** | **Used** |
| HexCer (d18:2/23:0) | Hexosylceramide d18:2/23:0 | **100.00** | 25.18 | **0.00** | Excluded |
| HexCer (d18:2/24:0) | Hexosylceramide d18:2/24:0 | **100.00** | **24.10** | **0.00** | **Used** |
| AbsAcid | Abscisic acid | 1.01 | 78.72 | **3.11** | Excluded |
| Cortisol | Cortisol | **99.86** | **10.60** | **0.00** | **Used** |
| Cortisone | Cortisone | **56.29** | **10.20** | **0.00** | **Used** |
| DHEAS | Dehydroepiandrosterone sulfate | **99.76** | 30.82 | **0.00** | Excluded |
| 3-IAA | 3-Indoleacetic acid | **100.00** | **21.37** | **0.00** | **Used** |
| 3-IPA | 3-Indolepropionic acid | **99.39** | **24.75** | **0.00** | **Used** |
| Ind-SO4 | Indoxyl sulfate | **100.00** | 29.86 | **0.00** | Excluded |
| Indole | Indole | 36.06 | 66.27 | **9.74** | Excluded |
| Hypoxanthine | Hypoxanthine | **98.44** | 25.85 | **0.00** | Excluded |
| Xanthine | Xanthine | **100.00** | 26.19 | **0.00** | Excluded |
| SM (OH) C14:1 | Hydroxysphingomyeline C14:1 | **100.00** | **15.78** | **0.00** | **Used** |
| SM (OH) C16:1 | Hydroxysphingomyeline C16:1 | **100.00** | **18.70** | **0.00** | **Used** |
| SM (OH) C22:1 | Hydroxysphingomyeline C22:1 | **100.00** | 38.67 | **0.00** | Excluded |
| SM (OH) C22:2 | Hydroxysphingomyeline C22:2 | **100.00** | 36.15 | **0.00** | Excluded |
| SM (OH) C24:1 | Hydroxysphingomyeline C24:1 | **100.00** | 48.62 | **0.00** | Excluded |
| SM C16:0 | Sphingomyelin C16:0 | **100.00** | **16.80** | **0.00** | **Used** |
| SM C16:1 | Sphingomyelin C16:1 | **100.00** | **16.91** | **0.00** | **Used** |
| SM C18:0 | Sphingomyelin C18:0 | **100.00** | **19.82** | **0.00** | **Used** |
| SM C18:1 | Sphingomyelin C18:1 | **100.00** | **18.60** | **0.00** | **Used** |
| SM C20:2 | Sphingomyelin C20:2 | **100.00** | **21.22** | **0.00** | **Used** |
| SM C22:3 | Sphingomyelin C22:3 | 34.91 | 58.14 | 63.26 | Excluded |
| SM C24:0 | Sphingomyelin C24:0 | **100.00** | 42.86 | **0.00** | Excluded |
| SM C24:1 | Sphingomyelin C24:1 | **100.00** | 42.25 | **0.00** | Excluded |
| SM C26:0 | Sphingomyelin C26:0 | **99.97** | 47.70 | **0.00** | Excluded |
| SM C26:1 | Sphingomyelin C26:1 | **100.00** | 41.92 | **0.00** | Excluded |
| H1 | Hexoses (including glucose) | **100.00** | **6.69** | **0.00** | **Used** |
| TG (14:0_32:2) | Triglyceride 14:0_32:2 | **96.58** | **22.47** | **0.00** | **Used** |
| TG (14:0_34:0) | Triglyceride 14:0_34:0 | **98.99** | **19.25** | **0.00** | **Used** |
| TG (14:0_34:1) | Triglyceride 14:0_34:1 | **100.00** | **16.64** | **0.00** | **Used** |
| TG (14:0_34:2) | Triglyceride 14:0_34:2 | **100.00** | **16.98** | **0.00** | **Used** |
| TG (14:0_34:3) | Triglyceride 14:0_34:3 | **99.97** | **17.05** | **0.00** | **Used** |
| TG (14:0_35:1) | Triglyceride 14:0_35:1 | **94.62** | **18.57** | **0.00** | **Used** |
| TG (14:0_35:2) | Triglyceride 14:0_35:2 | **95.57** | **20.12** | **0.00** | **Used** |
| TG (14:0_36:1) | Triglyceride 14:0_36:1 | **99.97** | **17.47** | **0.00** | **Used** |
| TG (14:0_36:2) | Triglyceride 14:0_36:2 | **100.00** | **16.93** | **0.00** | **Used** |
| TG (14:0_36:3) | Triglyceride 14:0_36:3 | **100.00** | **15.35** | **0.00** | **Used** |
| TG (14:0_36:4) | Triglyceride 14:0_36:4 | **100.00** | **15.62** | **0.00** | **Used** |
| TG (14:0_38:4) | Triglyceride 14:0_38:4 | **99.59** | **17.74** | **0.00** | **Used** |
| TG (14:0_38:5) | Triglyceride 14:0_38:5 | **99.49** | **17.71** | **0.00** | **Used** |
| TG (14:0_39:3) | Triglyceride 14:0_39:3 | 35.42 | 37.80 | **0.17** | Excluded |
| TG (16:0_28:1) | Triglyceride 16:0_28:1 | **94.05** | **22.26** | **0.03** | **Used** |
| TG (16:0_28:2) | Triglyceride 16:0_28:2 | **89.88** | **22.07** | **0.00** | **Used** |
| TG (16:0_30:2) | Triglyceride 16:0_30:2 | **99.29** | **19.58** | **0.00** | **Used** |
| TG (16:0_32:0) | Triglyceride 16:0_32:0 | **98.55** | **18.13** | **0.03** | **Used** |
| TG (16:0_32:1) | Triglyceride 16:0_32:1 | **99.93** | **17.23** | **0.00** | **Used** |
| TG (16:0_32:2) | Triglyceride 16:0_32:2 | **99.97** | **16.33** | **0.00** | **Used** |
| TG (16:0_32:3) | Triglyceride 16:0_32:3 | **99.97** | **16.96** | **0.00** | **Used** |
| TG (16:0_33:1) | Triglyceride 16:0_33:1 | **99.56** | **15.56** | **0.03** | **Used** |
| TG (16:0_33:2) | Triglyceride 16:0_33:2 | **99.32** | **15.83** | **0.00** | **Used** |
| TG (16:0_34:0) | Triglyceride 16:0_34:0 | **99.90** | **21.13** | **0.00** | **Used** |
| TG (16:0_34:1) | Triglyceride 16:0_34:1 | **100.00** | **22.01** | **0.00** | **Used** |
| TG (16:0_34:2) | Triglyceride 16:0_34:2 | **100.00** | **15.74** | **0.00** | **Used** |
| TG (16:0_34:3) | Triglyceride 16:0_34:3 | **100.00** | **15.51** | **0.00** | **Used** |
| TG (16:0_34:4) | Triglyceride 16:0_34:4 | **100.00** | **15.54** | **0.00** | **Used** |
| TG (16:0_35:1) | Triglyceride 16:0_35:1 | **99.83** | **23.57** | **0.00** | **Used** |
| TG (16:0_35:2) | Triglyceride 16:0_35:2 | **100.00** | **18.67** | **0.00** | **Used** |
| TG (16:0_35:3) | Triglyceride 16:0_35:3 | **100.00** | **15.81** | **0.00** | **Used** |
| TG (16:0_36:2) | Triglyceride 16:0_36:2 | **100.00** | **19.40** | **0.00** | **Used** |
| TG (16:0_36:3) | Triglyceride 16:0_36:3 | **100.00** | **18.25** | **0.00** | **Used** |
| TG (16:0_36:4) | Triglyceride 16:0_36:4 | **100.00** | **16.89** | **0.00** | **Used** |
| TG (16:0_36:5) | Triglyceride 16:0_36:5 | **100.00** | **16.20** | **0.00** | **Used** |
| TG (16:0_36:6) | Triglyceride 16:0_36:6 | **99.22** | **18.28** | **0.00** | **Used** |
| TG (16:0_37:3) | Triglyceride 16:0_37:3 | **99.73** | **23.88** | **0.00** | **Used** |
| TG (16:0_38:1) | Triglyceride 16:0_38:1 | **99.76** | 41.15 | **0.00** | Excluded |
| TG (16:0_38:2) | Triglyceride 16:0_38:2 | **100.00** | 33.51 | **0.00** | Excluded |
| TG (16:0_38:3) | Triglyceride 16:0_38:3 | **100.00** | 29.16 | **0.00** | Excluded |
| TG (16:0_38:4) | Triglyceride 16:0_38:4 | **100.00** | 26.91 | **0.00** | Excluded |
| TG (16:0_38:5) | Triglyceride 16:0_38:5 | **100.00** | 25.46 | **0.00** | Excluded |
| TG (16:0_38:6) | Triglyceride 16:0_38:6 | **100.00** | 25.25 | **0.00** | Excluded |
| TG (16:0_38:7) | Triglyceride 16:0_38:7 | **99.63** | 29.89 | **0.00** | Excluded |
| TG (16:0_40:6) | Triglyceride 16:0_40:6 | **100.00** | 29.67 | **0.00** | Excluded |
| TG (16:0_40:7) | Triglyceride 16:0_40:7 | **99.93** | 29.27 | **0.00** | Excluded |
| TG (16:0_40:8) | Triglyceride 16:0_40:8 | **99.70** | 26.79 | **0.00** | Excluded |
| TG (16:1_28:0) | Triglyceride 16:1_28:0 | **74.86** | 29.76 | **2.44** | Excluded |
| TG (16:1_30:1) | Triglyceride 16:1_30:1 | **89.88** | 28.04 | **1.12** | Excluded |
| TG (16:1_32:0) | Triglyceride 16:1_32:0 | **98.99** | **17.84** | **0.17** | **Used** |
| TG (16:1_32:1) | Triglyceride 16:1_32:1 | **99.05** | **17.84** | **0.14** | **Used** |
| TG (16:1_32:2) | Triglyceride 16:1_32:2 | **97.56** | **18.37** | **0.17** | **Used** |
| TG (16:1_33:1) | Triglyceride 16:1_33:1 | **92.15** | 28.01 | **0.95** | Excluded |
| TG (16:1_34:0) | Triglyceride 16:1_34:0 | **99.97** | **16.65** | **0.00** | **Used** |
| TG (16:1_34:1) | Triglyceride 16:1_34:1 | **100.00** | **16.98** | **0.00** | **Used** |
| TG (16:1_34:2) | Triglyceride 16:1_34:2 | **100.00** | **15.28** | **0.00** | **Used** |
| TG (16:1_34:3) | Triglyceride 16:1_34:3 | **100.00** | **15.25** | **0.00** | **Used** |
| TG (16:1_36:1) | Triglyceride 16:1_36:1 | **100.00** | **18.17** | **0.00** | **Used** |
| TG (16:1_36:2) | Triglyceride 16:1_36:2 | **100.00** | **18.07** | **0.00** | **Used** |
| TG (16:1_36:3) | Triglyceride 16:1_36:3 | **100.00** | **16.09** | **0.00** | **Used** |
| TG (16:1_36:4) | Triglyceride 16:1_36:4 | **100.00** | **16.09** | **0.00** | **Used** |
| TG (16:1_36:5) | Triglyceride 16:1_36:5 | **100.00** | **15.98** | **0.00** | **Used** |
| TG (16:1_38:3) | Triglyceride 16:1_38:3 | **99.76** | 26.84 | **0.00** | Excluded |
| TG (16:1_38:4) | Triglyceride 16:1_38:4 | **99.93** | 24.62 | **0.00** | **Used** |
| TG (16:1_38:5) | Triglyceride 16:1_38:5 | **100.00** | **22.76** | **0.00** | **Used** |
| TG (17:0_32:1) | Triglyceride 17:0_32:1 | **95.37** | **23.57** | **0.14** | **Used** |
| TG (17:0_34:1) | Triglyceride 17:0_34:1 | **99.86** | **20.40** | **0.00** | **Used** |
| TG (17:0_34:2) | Triglyceride 17:0_34:2 | **99.93** | **17.19** | **0.00** | **Used** |
| TG (17:0_34:3) | Triglyceride 17:0_34:3 | **99.46** | **20.12** | **0.00** | **Used** |
| TG (17:0_36:3) | Triglyceride 17:0_36:3 | **100.00** | **19.14** | **0.00** | **Used** |
| TG (17:0_36:4) | Triglyceride 17:0_36:4 | **99.97** | **19.96** | **0.00** | **Used** |
| TG (17:1_32:1) | Triglyceride 17:1_32:1 | **95.37** | **19.37** | **0.17** | **Used** |
| TG (17:1_34:1) | Triglyceride 17:1_34:1 | **100.00** | **17.55** | **0.00** | **Used** |
| TG (17:1_34:2) | Triglyceride 17:1_34:2 | **99.93** | **16.44** | **0.00** | **Used** |
| TG (17:1_34:3) | Triglyceride 17:1_34:3 | **95.53** | **24.64** | **0.00** | **Used** |
| TG (17:1_36:3) | Triglyceride 17:1_36:3 | **100.00** | **17.95** | **0.00** | **Used** |
| TG (17:1_36:4) | Triglyceride 17:1_36:4 | **99.49** | **19.74** | **0.00** | **Used** |
| TG (17:1_36:5) | Triglyceride 17:1_36:5 | **92.59** | **24.52** | **0.00** | **Used** |
| TG (17:1_38:5) | Triglyceride 17:1_38:5 | **92.79** | 26.86 | **0.00** | Excluded |
| TG (17:1_38:6) | Triglyceride 17:1_38:6 | **83.80** | 30.56 | **0.00** | Excluded |
| TG (17:1_38:7) | Triglyceride 17:1_38:7 | **50.64** | 40.26 | **0.24** | Excluded |
| TG (17:2_34:2) | Triglyceride 17:2_34:2 | **98.88** | **22.87** | **0.00** | **Used** |
| TG (17:2_34:3) | Triglyceride 17:2_34:3 | **94.82** | **22.25** | **0.00** | **Used** |
| TG (17:2_36:2) | Triglyceride 17:2_36:2 | **99.36** | **24.39** | **0.00** | **Used** |
| TG (17:2_36:3) | Triglyceride 17:2_36:3 | **97.87** | **22.65** | **0.00** | **Used** |
| TG (17:2_36:4) | Triglyceride 17:2_36:4 | **99.83** | 26.43 | **0.00** | Excluded |
| TG (17:2_38:5) | Triglyceride 17:2_38:5 | **90.76** | 28.00 | **0.00** | Excluded |
| TG (17:2_38:6) | Triglyceride 17:2_38:6 | **65.90** | 34.38 | **0.00** | Excluded |
| TG (17:2_38:7) | Triglyceride 17:2_38:7 | **85.32** | 35.21 | **0.07** | Excluded |
| TG (18:0_30:0) | Triglyceride 18:0_30:0 | **89.34** | **18.75** | **0.20** | **Used** |
| TG (18:0_30:1) | Triglyceride 18:0_30:1 | **95.47** | **18.10** | **0.07** | **Used** |
| TG (18:0_32:0) | Triglyceride 18:0_32:0 | **95.64** | **20.18** | **0.03** | **Used** |
| TG (18:0_32:1) | Triglyceride 18:0_32:1 | **99.90** | **17.77** | **0.03** | **Used** |
| TG (18:0_32:2) | Triglyceride 18:0_32:2 | **99.66** | **19.06** | **0.00** | **Used** |
| TG (18:0_34:2) | Triglyceride 18:0_34:2 | **100.00** | **20.13** | **0.00** | **Used** |
| TG (18:0_34:3) | Triglyceride 18:0_34:3 | **100.00** | **19.70** | **0.00** | **Used** |
| TG (18:0_36:1) | Triglyceride 18:0_36:1 | **99.63** | **21.68** | **0.10** | **Used** |
| TG (18:0_36:2) | Triglyceride 18:0_36:2 | **99.86** | 38.53 | **0.07** | Excluded |
| TG (18:0_36:3) | Triglyceride 18:0_36:3 | **100.00** | 27.40 | **0.00** | Excluded |
| TG (18:0_36:4) | Triglyceride 18:0_36:4 | **100.00** | 27.60 | **0.00** | Excluded |
| TG (18:0_36:5) | Triglyceride 18:0_36:5 | **100.00** | **22.66** | **0.00** | **Used** |
| TG (18:0_38:6) | Triglyceride 18:0_38:6 | **100.00** | 27.40 | **0.00** | Excluded |
| TG (18:0_38:7) | Triglyceride 18:0_38:7 | **97.90** | 28.23 | **0.00** | Excluded |
| TG (18:1_26:0) | Triglyceride 18:1_26:0 | **97.73** | **22.13** | **0.00** | **Used** |
| TG (18:1_28:1) | Triglyceride 18:1_28:1 | **98.00** | **20.16** | **0.03** | **Used** |
| TG (18:1_30:0) | Triglyceride 18:1_30:0 | **99.97** | **16.54** | **0.00** | **Used** |
| TG (18:1_30:1) | Triglyceride 18:1_30:1 | **99.93** | **16.83** | **0.00** | **Used** |
| TG (18:1_30:2) | Triglyceride 18:1_30:2 | **99.97** | **17.22** | **0.00** | **Used** |
| TG (18:1_31:0) | Triglyceride 18:1_31:0 | **98.24** | **15.09** | **0.00** | **Used** |
| TG (18:1_32:0) | Triglyceride 18:1_32:0 | **100.00** | **17.05** | **0.00** | **Used** |
| TG (18:1_32:1) | Triglyceride 18:1_32:1 | **100.00** | **16.61** | **0.00** | **Used** |
| TG (18:1_32:2) | Triglyceride 18:1_32:2 | **100.00** | **15.50** | **0.00** | **Used** |
| TG (18:1_32:3) | Triglyceride 18:1_32:3 | **100.00** | **15.12** | **0.00** | **Used** |
| TG (18:1_33:0) | Triglyceride 18:1_33:0 | **99.97** | **19.65** | **0.00** | **Used** |
| TG (18:1_33:1) | Triglyceride 18:1_33:1 | **100.00** | **18.27** | **0.00** | **Used** |
| TG (18:1_33:2) | Triglyceride 18:1_33:2 | **99.97** | **16.46** | **0.00** | **Used** |
| TG (18:1_33:3) | Triglyceride 18:1_33:3 | **99.53** | **18.17** | **0.00** | **Used** |
| TG (18:1_34:1) | Triglyceride 18:1_34:1 | **100.00** | **18.75** | **0.00** | **Used** |
| TG (18:1_34:2) | Triglyceride 18:1_34:2 | **100.00** | **18.01** | **0.00** | **Used** |
| TG (18:1_34:3) | Triglyceride 18:1_34:3 | **100.00** | **16.43** | **0.00** | **Used** |
| TG (18:1_34:4) | Triglyceride 18:1_34:4 | **100.00** | **17.15** | **0.00** | **Used** |
| TG (18:1_35:2) | Triglyceride 18:1_35:2 | **100.00** | **20.25** | **0.00** | **Used** |
| TG (18:1_35:3) | Triglyceride 18:1_35:3 | **100.00** | **18.37** | **0.00** | **Used** |
| TG (18:1_36:0) | Triglyceride 18:1_36:0 | **100.00** | 33.67 | **0.00** | Excluded |
| TG (18:1_36:1) | Triglyceride 18:1_36:1 | **100.00** | 30.45 | **0.00** | Excluded |
| TG (18:1_36:2) | Triglyceride 18:1_36:2 | **100.00** | 28.10 | **0.00** | Excluded |
| TG (18:1_36:3) | Triglyceride 18:1_36:3 | **100.00** | 25.88 | **0.00** | Excluded |
| TG (18:1_36:4) | Triglyceride 18:1_36:4 | **100.00** | **24.13** | **0.00** | **Used** |
| TG (18:1_36:5) | Triglyceride 18:1_36:5 | **100.00** | **21.61** | **0.00** | **Used** |
| TG (18:1_36:6) | Triglyceride 18:1_36:6 | **99.80** | **21.66** | **0.00** | **Used** |
| TG (18:1_38:5) | Triglyceride 18:1_38:5 | **100.00** | 27.27 | **0.00** | Excluded |
| TG (18:1_38:6) | Triglyceride 18:1_38:6 | **100.00** | 27.16 | **0.00** | Excluded |
| TG (18:1_38:7) | Triglyceride 18:1_38:7 | **99.93** | **24.38** | **0.00** | **Used** |
| TG (18:2_28:0) | Triglyceride 18:2_28:0 | **99.29** | **20.38** | **0.00** | **Used** |
| TG (18:2_30:0) | Triglyceride 18:2_30:0 | **100.00** | **16.32** | **0.00** | **Used** |
| TG (18:2_30:1) | Triglyceride 18:2_30:1 | **100.00** | **17.34** | **0.00** | **Used** |
| TG (18:2_31:0) | Triglyceride 18:2_31:0 | **91.07** | **15.33** | **0.00** | **Used** |
| TG (18:2_32:0) | Triglyceride 18:2_32:0 | **100.00** | **16.73** | **0.00** | **Used** |
| TG (18:2_32:1) | Triglyceride 18:2_32:1 | **100.00** | **14.93** | **0.00** | **Used** |
| TG (18:2_32:2) | Triglyceride 18:2_32:2 | **100.00** | **15.26** | **0.00** | **Used** |
| TG (18:2_33:0) | Triglyceride 18:2_33:0 | **99.93** | **18.76** | **0.00** | **Used** |
| TG (18:2_33:1) | Triglyceride 18:2_33:1 | **99.97** | **15.84** | **0.00** | **Used** |
| TG (18:2_33:2) | Triglyceride 18:2_33:2 | **99.90** | **16.19** | **0.00** | **Used** |
| TG (18:2_34:0) | Triglyceride 18:2_34:0 | **100.00** | **18.15** | **0.00** | **Used** |
| TG (18:2_34:1) | Triglyceride 18:2_34:1 | **100.00** | **18.14** | **0.00** | **Used** |
| TG (18:2_34:2) | Triglyceride 18:2_34:2 | **100.00** | **15.82** | **0.00** | **Used** |
| TG (18:2_34:3) | Triglyceride 18:2_34:3 | **100.00** | **15.88** | **0.00** | **Used** |
| TG (18:2_34:4) | Triglyceride 18:2_34:4 | **99.97** | **15.57** | **0.00** | **Used** |
| TG (18:2_35:1) | Triglyceride 18:2_35:1 | **100.00** | **21.15** | **0.00** | **Used** |
| TG (18:2_35:2) | Triglyceride 18:2_35:2 | **99.97** | **18.42** | **0.00** | **Used** |
| TG (18:2_35:3) | Triglyceride 18:2_35:3 | **99.86** | **18.36** | **0.00** | **Used** |
| TG (18:2_36:0) | Triglyceride 18:2_36:0 | **100.00** | 29.36 | **0.00** | Excluded |
| TG (18:2_36:1) | Triglyceride 18:2_36:1 | **100.00** | 26.79 | **0.00** | Excluded |
| TG (18:2_36:2) | Triglyceride 18:2_36:2 | **100.00** | 26.13 | **0.00** | Excluded |
| TG (18:2_36:3) | Triglyceride 18:2_36:3 | **100.00** | **23.92** | **0.00** | **Used** |
| TG (18:2_36:4) | Triglyceride 18:2_36:4 | **100.00** | **24.06** | **0.00** | **Used** |
| TG (18:2_36:5) | Triglyceride 18:2_36:5 | **99.97** | **19.49** | **0.00** | **Used** |
| TG (18:2_38:4) | Triglyceride 18:2_38:4 | **100.00** | 28.08 | **0.00** | Excluded |
| TG (18:2_38:5) | Triglyceride 18:2_38:5 | **100.00** | 26.53 | **0.00** | Excluded |
| TG (18:2_38:6) | Triglyceride 18:2_38:6 | **100.00** | 26.22 | **0.00** | Excluded |
| TG (18:3_30:0) | Triglyceride 18:3_30:0 | **99.90** | **16.34** | **0.00** | **Used** |
| TG (18:3_32:0) | Triglyceride 18:3_32:0 | **96.89** | **18.18** | **0.00** | **Used** |
| TG (18:3_32:1) | Triglyceride 18:3_32:1 | **99.83** | **19.38** | **0.00** | **Used** |
| TG (18:3_33:2) | Triglyceride 18:3_33:2 | **88.23** | 52.06 | **0.03** | Excluded |
| TG (18:3_34:0) | Triglyceride 18:3_34:0 | **99.93** | 18.54 | **0.00** | **Used** |
| TG (18:3_34:1) | Triglyceride 18:3_34:1 | **100.00** | **16.67** | **0.00** | **Used** |
| TG (18:3_34:2) | Triglyceride 18:3_34:2 | **100.00** | **15.71** | **0.00** | **Used** |
| TG (18:3_34:3) | Triglyceride 18:3_34:3 | **99.22** | **19.25** | **0.00** | **Used** |
| TG (18:3_35:2) | Triglyceride 18:3_35:2 | **97.87** | 27.25 | **0.00** | Excluded |
| TG (18:3_36:1) | Triglyceride 18:3_36:1 | **99.97** | 28.43 | **0.00** | Excluded |
| TG (18:3_36:2) | Triglyceride 18:3_36:2 | **100.00** | **24.55** | **0.00** | **Used** |
| TG (18:3_36:3) | Triglyceride 18:3_36:3 | **100.00** | **22.68** | **0.00** | **Used** |
| TG (18:3_36:4) | Triglyceride 18:3_36:4 | **99.97** | **20.81** | **0.00** | **Used** |
| TG (18:3_38:5) | Triglyceride 18:3_38:5 | **99.46** | 25.04 | **0.00** | Excluded |
| TG (18:3_38:6) | Triglyceride 18:3_38:6 | **94.22** | 30.78 | **0.00** | Excluded |
| TG (20:0_32:3) | Triglyceride 20:0_32:3 | **99.90** | **20.59** | **0.00** | **Used** |
| TG (20:0_32:4) | Triglyceride 20:0_32:4 | **98.38** | **19.57** | **0.00** | **Used** |
| TG (20:0_34:1) | Triglyceride 20:0_34:1 | 47.50 | 53.51 | **0.00** | Excluded |
| TG (20:1_24:3) | Triglyceride 20:1_24:3 | 2.50 | 81.11 | **0.00** | Excluded |
| TG (20:1_26:1) | Triglyceride 20:1_26:1 | 4.19 | 87.96 | **0.00** | Excluded |
| TG (20:1_30:1) | Triglyceride 20:1_30:1 | **78.15** | **21.66** | **0.07** | **Used** |
| TG (20:1_31:0) | Triglyceride 20:1_31:0 | 0.07 | 62.08 | **0.00** | Excluded |
| TG (20:1_32:1) | Triglyceride 20:1_32:1 | **99.22** | **19.14** | **0.00** | **Used** |
| TG (20:1_32:2) | Triglyceride 20:1_32:2 | **95.03** | **17.30** | **0.00** | **Used** |
| TG (20:1_32:3) | Triglyceride 20:1_32:3 | **82.81** | **23.08** | **0.00** | **Used** |
| TG (20:1_34:0) | Triglyceride 20:1_34:0 | **93.37** | 30.24 | **0.00** | Excluded |
| TG (20:1_34:1) | Triglyceride 20:1_34:1 | **99.97** | 29.05 | **0.00** | Excluded |
| TG (20:1_34:2) | Triglyceride 20:1_34:2 | **100.00** | **24.04** | **0.00** | **Used** |
| TG (20:1_34:3) | Triglyceride 20:1_34:3 | **97.09** | 26.38 | **0.00** | Excluded |
| TG (20:2_32:0) | Triglyceride 20:2_32:0 | **94.62** | **19.22** | **0.00** | **Used** |
| TG (20:2_32:1) | Triglyceride 20:2_32:1 | **99.63** | **18.69** | **0.00** | **Used** |
| TG (20:2_34:1) | Triglyceride 20:2_34:1 | **100.00** | 28.06 | **0.00** | Excluded |
| TG (20:2_34:2) | Triglyceride 20:2_34:2 | **99.97** | 26.55 | **0.00** | Excluded |
| TG (20:2_34:3) | Triglyceride 20:2_34:3 | **98.95** | 26.61 | **0.00** | Excluded |
| TG (20:2_34:4) | Triglyceride 20:2_34:4 | **66.75** | 34.70 | **0.10** | Excluded |
| TG (20:2_36:5) | Triglyceride 20:2_36:5 | **85.55** | 28.91 | **0.00** | Excluded |
| TG (20:3_32:0) | Triglyceride 20:3_32:0 | **99.56** | **19.12** | **0.00** | **Used** |
| TG (20:3_32:1) | Triglyceride 20:3_32:1 | **100.00** | **17.60** | **0.00** | **Used** |
| TG (20:3_32:2) | Triglyceride 20:3_32:2 | **98.75** | **20.57** | **0.00** | **Used** |
| TG (20:3_34:0) | Triglyceride 20:3_34:0 | **99.53** | 30.51 | **0.00** | Excluded |
| TG (20:3_34:1) | Triglyceride 20:3_34:1 | **100.00** | 28.42 | **0.00** | Excluded |
| TG (20:3_34:2) | Triglyceride 20:3_34:2 | **100.00** | **23.43** | **0.00** | **Used** |
| TG (20:3_34:3) | Triglyceride 20:3_34:3 | **99.93** | 25.71 | **0.00** | Excluded |
| TG (20:3_36:3) | Triglyceride 20:3_36:3 | **100.00** | 25.15 | **0.00** | Excluded |
| TG (20:3_36:4) | Triglyceride 20:3_36:4 | **99.97** | 28.51 | **0.00** | Excluded |
| TG (20:3_36:5) | Triglyceride 20:3_36:5 | **92.96** | 25.38 | **0.00** | Excluded |
| TG (20:4_30:0) | Triglyceride 20:4_30:0 | **99.12** | **17.14** | **0.00** | **Used** |
| TG (20:4_32:0) | Triglyceride 20:4_32:0 | **100.00** | **18.03** | **0.00** | **Used** |
| TG (20:4_32:1) | Triglyceride 20:4_32:1 | **100.00** | **16.64** | **0.00** | **Used** |
| TG (20:4_32:2) | Triglyceride 20:4_32:2 | **99.63** | **17.76** | **0.00** | **Used** |
| TG (20:4_33:2) | Triglyceride 20:4_33:2 | **91.81** | **21.33** | **0.00** | **Used** |
| TG (20:4_34:0) | Triglyceride 20:4_34:0 | **100.00** | 26.29 | **0.00** | Excluded |
| TG (20:4_34:1) | Triglyceride 20:4_34:1 | **100.00** | 25.66 | **0.00** | Excluded |
| TG (20:4_34:2) | Triglyceride 20:4_34:2 | **100.00** | **22.78** | **0.00** | **Used** |
| TG (20:4_34:3) | Triglyceride 20:4_34:3 | **99.97** | **21.68** | **0.00** | **Used** |
| TG (20:4_35:3) | Triglyceride 20:4_35:3 | **83.46** | 28.41 | **0.00** | Excluded |
| TG (20:4_36:2) | Triglyceride 20:4_36:2 | **100.00** | 27.92 | **0.00** | Excluded |
| TG (20:4_36:3) | Triglyceride 20:4_36:3 | **100.00** | 26.34 | **0.00** | Excluded |
| TG (20:4_36:4) | Triglyceride 20:4_36:4 | **100.00** | **24.36** | **0.00** | **Used** |
| TG (20:4_36:5) | Triglyceride 20:4_36:5 | **98.24** | **23.92** | **0.00** | **Used** |
| TG (20:5_34:0) | Triglyceride 20:5_34:0 | **96.55** | 32.76 | **0.00** | Excluded |
| TG (20:5_34:1) | Triglyceride 20:5_34:1 | **100.00** | **24.14** | **0.00** | **Used** |
| TG (20:5_34:2) | Triglyceride 20:5_34:2 | **99.97** | **21.08** | **0.00** | **Used** |
| TG (20:5_36:2) | Triglyceride 20:5_36:2 | **100.00** | 28.18 | **0.00** | Excluded |
| TG (20:5_36:3) | Triglyceride 20:5_36:3 | **99.90** | **24.94** | **0.00** | **Used** |
| TG (22:0_32:4) | Triglyceride 22:0_32:4 | **54.63** | 33.28 | **0.10** | Excluded |
| TG (22:1_32:5) | Triglyceride 22:1_32:5 | **61.03** | 35.83 | **0.00** | Excluded |
| TG (22:2_32:4) | Triglyceride 22:2_32:4 | **51.49** | 30.30 | **0.03** | Excluded |
| TG (22:3_30:2) | Triglyceride 22:3_30:2 | 8.49 | 40.54 | **0.03** | Excluded |
| TG (22:4_32:0) | Triglyceride 22:4_32:0 | **97.33** | 28.59 | **0.00** | Excluded |
| TG (22:4_32:2) | Triglyceride 22:4_32:2 | **75.27** | 26.19 | **0.00** | Excluded |
| TG (22:4_34:2) | Triglyceride 22:4_34:2 | **99.86** | 27.38 | **0.00** | Excluded |
| TG (22:5_32:0) | Triglyceride 22:5_32:0 | **98.61** | 28.59 | **0.00** | Excluded |
| TG (22:5_32:1) | Triglyceride 22:5_32:1 | **100.00** | 25.44 | **0.00** | Excluded |
| TG (22:5_34:1) | Triglyceride 22:5_34:1 | **99.97** | 28.88 | **0.00** | Excluded |
| TG (22:5_34:2) | Triglyceride 22:5_34:2 | **100.00** | 27.45 | **0.00** | Excluded |
| TG (22:5_34:3) | Triglyceride 22:5_34:3 | **99.12** | **24.66** | **0.00** | **Used** |
| TG (22:6_32:0) | Triglyceride 22:6_32:0 | **99.90** | 28.54 | **0.00** | Excluded |
| TG (22:6_32:1) | Triglyceride 22:6_32:1 | **100.00** | **23.46** | **0.00** | **Used** |
| TG (22:6_34:1) | Triglyceride 22:6_34:1 | **100.00** | 29.13 | **0.00** | Excluded |
| TG (22:6_34:2) | Triglyceride 22:6_34:2 | **100.00** | 26.23 | **0.00** | Excluded |
| TG (22:6_34:3) | Triglyceride 22:6_34:3 | **99.53** | **24.87** | **0.00** | **Used** |
| Choline | Choline | **100.00** | **10.17** | **0.00** | **Used** |

The first and second columns display the metabolite abbreviations and biochemical names, respectively. For each metabolite, the percentage of the 2,956 measured KORA-Fit samples above the limit of detection (LOD) is presented. Median relative standard deviations (RSD) of 360 quality control samples (QC1, QC2, QC3, NIST, and five reference sample on each of the 40 utilized kit plates) are listed. The percentage of missing values in the total 2,956 measured samples is also shown. The used or excluded metabolite is shown in the last column. Used metabolites met all three criteria: (1) more than 50% of the 2,956 measured sample concentrations are above the LOD, (2) a median RSD below 25%, and (3) fewer than 10% missing values among the 2,956 samples. Values that met each of the three criteria are shown in bold.

## Table S2. Characteristics of the four mouse groups

| **Clinical parameters** | | **WT** | **VG-db/db** | **MET-db/db** | **COMBI-db/db** |
| --- | --- | --- | --- | --- | --- |
|  |  | **(n = 10)** | **(n = 10)** | **(n = 10)** | **(n = 10)** |
| **Weight, g** | **Body** | 22.0 ± 0.6 | 47.9 ± 2.4 | 47.8 ± 2.1 | 46.8 ± 1.7 |
|  | **Liver** | 1.02 ± 0.09 | 2.56 ± 0.29 | 2.61 ± 0.09 | 2.36 ± 0.19 |
|  | **Kidney** | 0.16 ± 0.02 | 0.20 ± 0.02 | 0.21 ± 0.02 | 0.21 ± 0.02 |
| **Blood glucose, mg/dL** | **6 weeks** | 108.8 ± 14.3 | 442.5 ± 65.1 | 454.8 ± 60.2 | 439.1 ± 62.0 |
|  | **8 weeks** | 106.7 ± 16.8 | 421.6 ± 41.2 | 322.6 ± 92.7 | 129.9 ± 46.3 |
|  | **Changed, %** | 1.9 | 4.7 | 29.1 | 70.1 |
| **HbA_1C_, %** | **8 weeks** | 4.3 ± 0.2 | 6.1 ± 0.5 | 6.3 ± 0.6 | 6.2 ± 0.5 |
|  | **HDL** | 84.3 ± 8.6 | 125.3 ± 13.1 | 135.5 ± 9.8 | 156.0 ± 22.9 |
| **Cholesterol, mg/dL** | **LDL** | 14.5 ± 2.1 | 18.8 ± 3.7 | 19.5 ± 2.6 | 25.2 ± 7.6 |
|  | **Total** | 100.6 ± 12.2 | 153.2 ± 16.1 | 164.5 ± 12.6 | 188.8 ± 29.9 |
|  | **Triglycerides** | 122.2 ± 24.5 | 224.8 ± 106.5 | 262.4 ± 63.4 | 199.9 ± 46.7 |
| **Albumin, g/dL** | **8 weeks** | 2.6 ± 0.1 | 3.1 ± 0.3 | 3.3 ± 0.3 | 3.7 ± 0.3 |
| **C-reactive protein, mg/L** | **8 weeks** | 5.4 ± 1.1 | 13.1 ± 3.3 | 14.0 ± 4.1 | 17.1 ± 3.7 |
| **Insulin, µg/L** | **8 weeks** | 1.0 ± 0.4 | 7.8 ± 2.3 | 7.9 ± 1.7 | 6.8 ± 2.4 |

Means ± standard deviation of clinical variables in four mice groups are shown: wild type mice (WT), vehicle‑gavaged diabetic mice (VG-db/db), metformin-treated diabetic mice (MET-db/db), and diabetic mice treated with both a Sodium-glucose-cotransporter-2-inhibitor and metformin (COMBI-db/db). **Abbreviations:** HbA_1C_, hemoglobin A_1C_; HDL, high-density lipoprotein; LDL, low-density lipoprotein.

## Table S3. List of metabolites utilized in human serum and seven murine tissues

| Metabolites | Human Serum | Murine Plasma | Liver | Adrenal  gland | Adipose tissue | Testis | Lung | Cerebellum |
| --- | --- | --- | --- | --- | --- | --- | --- | --- |
| H1 | x | x | x | x | x | x | x | x |
| Lac | x | - | - | - | - | - | - | - |
| C0 | x | x | x | x | x | x | x | x |
| C2 | x | x | x | x | x | x | x | x |
| C3 | x | x | x | x | x | x | x | x |
| C3:1 | - | x | x | x | x | x | x | x |
| C3-DC (C4-OH)* | - | x | x | x | x | x | x | x |
| C3-OH | - | x | x | x | x | x | x | x |
| C4 | x | x | x | x | x | x | x | x |
| C4:1 | - | x | x | x | x | x | x | x |
| C5 | x | x | x | x | x | x | x | x |
| C5:1 | - | x | x | x | x | x | x | x |
| C5:1-DC | - | x | x | x | x | x | x | x |
| C5-DC (C6-OH)* | - | x | x | x | x | x | x | x |
| C5-M-DC | - | x | x | x | x | x | x | x |
| C5-OH (C3-DC-M)* | - | x | x | x | x | x | x | x |
| C6 (C4:1-DC)* | - | x | x | x | x | x | x | x |
| C6:1 | - | x | x | x | x | x | x | x |
| C7-DC | - | x | x | x | x | x | x | x |
| C8 | - | x | x | x | x | x | x | x |
| C9 | - | x | x | x | x | x | x | x |
| C10 | x | x | x | x | x | x | x | x |
| C10:1 | - | x | x | x | x | x | x | x |
| C10:2 | - | x | x | x | x | x | x | x |
| C12 | x | x | x | x | x | x | x | x |
| C12:1 | - | x | x | x | x | x | x | x |
| C12-DC | - | x | x | x | x | x | x | x |
| C14 | - | x | x | x | x | x | x | x |
| C14:1 | x | x | x | x | x | x | x | x |
| C14:1-OH | - | x | x | x | x | x | x | x |
| C14:2 | - | x | x | x | x | x | x | x |
| C14:2-OH | - | x | x | x | x | x | x | x |
| C16 | x | x | x | x | x | x | x | x |
| C16:1 | - | x | x | x | x | x | x | x |
| C16:1-OH | - | x | x | x | x | x | x | x |
| C16:2 | - | x | x | x | x | x | x | x |
| C16:2-OH | - | x | x | x | x | x | x | x |
| C16-OH | - | x | x | x | x | x | x | x |
| C18 | x | x | x | x | x | x | x | x |
| C18:1 | x | x | x | x | x | x | x | x |
| C18:1-OH | - | x | x | x | x | x | x | x |
| C18:2 | x | x | x | x | x | x | x | x |
| Ala | x | x | x | x | x | x | x | x |
| Arg | x | x | x | x | x | x | x | x |
| Asn | x | x | x | x | - | x | x | x |
| Asp | x | x | x | x | x | x | x | x |
| Cit | x | x | x | x | - | x | x | x |
| Gln | x | x | x | x | x | x | x | x |
| Glu | x | x | x | x | x | - | x | - |
| Gly | x | x | x | x | x | x | x | x |
| His | x | x | x | x | x | x | x | x |
| Ile | x | x | x | x | x | x | x | x |
| Leu | x | x | x | x | x | x | x | x |
| Lys | x | x | x | x | x | x | x | x |
| Met | x | x | x | x | - | x | x | x |
| Orn | x | x | x | x | x | x | x | x |
| Phe | x | x | x | x | x | x | x | x |
| Pro | x | x | x | x | x | x | x | x |
| Ser | x | x | x | x | - | x | x | x |
| Thr | x | x | x | x | x | x | - | x |
| Trp | x | x | x | x | x | x | - | x |
| Tyr | x | x | x | x | x | x | - | x |
| Val | x | x | x | x | - | x | - | x |
| 1-Met-His | x | - | - | - | - | - | - | - |
| 3-Met-His | x | - | - | - | - | - | - | - |
| AABA | x | - | - | - | - | - | - | - |
| alpha-AAA | x | x | x | x | - | x | x | x |
| Ac-Orn | - | x | x | - | - | x | x | x |
| ADMA | x | x | x | x | - | x | x | - |
| beta-Ala | x | - | - | - | - | - | - | - |
| Choline | x | - | - | - | - | - | - | - |
| Creatinine | x | x | x | x | x | x | x | x |
| Dopamine | - | - | - | x | x | - | - | - |
| GABA | x | - | - | - | - | - | - | - |
| HArg | x | - | - | - | - | - | - | - |
| Carnosine | - | x | - | - | x | x | x | x |
| Histamine | - | x | x | x | x | x | x | x |
| Kynurenine | x | x | x | x | - | x | x | x |
| Met-SO | x | x | x | - | x | x | x | - |
| ProBetaine | x | - | - | - | - | - | - | - |
| Putrescine | - | x | x | x | x | x | x | x |
| Sarcosine | x | x | x | x | x | x | x | x |
| SDMA | x | - | x | - | x | - | - | - |
| Serotonin | - | x | x | x | x | x | x | x |
| Spermidine | - | x | x | x | - | x | x | x |
| Spermine | - | x | x | x | x | x | - | x |
| t4-OH-Pro | x | - | - | - | - | - | - | - |
| Taurine | x | x | x | x | x | x | - | x |
| TrpBetaine | x | - | - | - | - | - | - | - |
| 3-IAA | x | - | - | - | - | - | - | - |
| 3-IPA | x | - | - | - | - | - | - | - |
| Cortisol | x | - | - | - | - | - | - | - |
| Cortisone | x | - | - | - | - | - | - | - |
| CA | x | - | - | - | - | - | - | - |
| CDCA | x | - | - | - | - | - | - | - |
| DCA | x | - | - | - | - | - | - | - |
| GCA | x | - | - | - | - | - | - | - |
| GDCA | x | - | - | - | - | - | - | - |
| GCDCA | x | - | - | - | - | - | - | - |
| GUDCA | x | - | - | - | - | - | - | - |
| TCA | x | - | - | - | - | - | - | - |
| TCDCA | x | - | - | - | - | - | - | - |
| TDCA | x | - | - | - | - | - | - | - |
| CE (18:2) | x | - | - | - | - | - | - | - |
| CE (18:3) | x | - | - | - | - | - | - | - |
| CE (20:0) | x | - | - | - | - | - | - | - |
| CE (20:1) | x | - | - | - | - | - | - | - |
| CE (20:3) | x | - | - | - | - | - | - | - |
| CE (20:4) | x | - | - | - | - | - | - | - |
| CE (20:5) | x | - | - | - | - | - | - | - |
| CE (22:2) | x | - | - | - | - | - | - | - |
| CE (22:5) | x | - | - | - | - | - | - | - |
| CE (22:6) | x | - | - | - | - | - | - | - |
| Cer (d18:0/24:0) | x | - | - | - | - | - | - | - |
| Cer (d18:0/24:1) | x | - | - | - | - | - | - | - |
| DG(16:0_16:1) | x | - | - | - | - | - | - | - |
| DG(16:0_18:1) | x | - | - | - | - | - | - | - |
| DG(16:0_18:2) | x | - | - | - | - | - | - | - |
| DG(16:1_18:2) | x | - | - | - | - | - | - | - |
| DG(17:0_18:1) | x | - | - | - | - | - | - | - |
| DG(18:1_18:1) | x | - | - | - | - | - | - | - |
| DG(18:1_18:2) | x | - | - | - | - | - | - | - |
| DG(18:1_18:3) | x | - | - | - | - | - | - | - |
| DG(18:1_20:0) | x | - | - | - | - | - | - | - |
| DG(18:1_20:1) | x | - | - | - | - | - | - | - |
| DG(18:2_18:2) | x | - | - | - | - | - | - | - |
| Hex2Cer (d18:1/16:0) | x | - | - | - | - | - | - | - |
| Hex2Cer (d18:1/18:0) | x | - | - | - | - | - | - | - |
| HexCer (d16:1/22:0) | x | - | - | - | - | - | - | - |
| HexCer (d18:1/16:0) | x | - | - | - | - | - | - | - |
| HexCer (d18:1/18:0) | x | - | - | - | - | - | - | - |
| HexCer (d18:1/18:1) | x | - | - | - | - | - | - | - |
| HexCer (d18:1/20:0) | x | - | - | - | - | - | - | - |
| HexCer (d18:1/22:0) | x | - | - | - | - | - | - | - |
| HexCer (d18:1/23:0) | x | - | - | - | - | - | - | - |
| HexCer (d18:1/24:0) | x | - | - | - | - | - | - | - |
| HexCer (d18:1/24:1) | x | - | - | - | - | - | - | - |
| HexCer (d18:2/22:0) | x | - | - | - | - | - | - | - |
| HexCer (d18:2/24:0) | x | - | - | - | - | - | - | - |
| PC aa C24:0 | - | x | x | x | x | x | x | x |
| PC aa C26:0 | - | x | x | x | x | x | x | x |
| PC aa C28:1 | x | x | x | x | x | x | x | x |
| PC aa C30:0 | x | x | x | x | x | x | x | x |
| PC aa C30:2 | - | - | x | x | x | - | x | x |
| PC aa C32:0 | - | x | x | x | x | x | x | x |
| PC aa C32:1 | - | x | x | x | x | x | x | x |
| PC aa C32:2 | - | x | x | x | x | x | x | x |
| PC aa C32:3 | - | x | x | x | x | x | x | x |
| PC aa C34:1 | - | x | x | x | x | x | x | x |
| PC aa C34:2 | - | x | x | x | x | x | x | x |
| PC aa C34:3 | - | x | x | x | x | x | x | x |
| PC aa C34:4 | - | x | x | x | x | x | x | x |
| PC aa C36:0 | - | x | x | x | x | x | x |  |
| PC aa C36:1 | - | x | x | x | x | x | x | x |
| PC aa C36:2 | - | x | x | x | x | x | x | x |
| PC aa C36:3 | - | x | x | x | x | x | x | x |
| PC aa C36:4 | - | x | x | x | x | x | x | x |
| PC aa C36:5 | - | x | x | x | x | x | x | x |
| PC aa C36:6 | - | x | x | x | x | x | x | x |
| PC aa C38:0 | - | x | x | x | x | x | x | x |
| PC aa C38:1 | - | x | x | x | x | x | x | x |
| PC aa C38:3 | - | x | x | x | x | x | x | x |
| PC aa C38:4 | - | x | x | x | x | x | x | x |
| PC aa C38:5 | - | x | x | x | x | x | x | x |
| PC aa C38:6 | - | x | x | x | x | x | x | x |
| PC aa C40:1 | - | x | x | x | x | x | x | x |
| PC aa C40:2 | x | x | x | x | x | x | x | x |
| PC aa C40:3 | - | x | x | x | x | x | x | x |
| PC aa C40:4 | x | x | x | x | x | x | x | x |
| PC aa C40:5 | x | x | x | x | x | x | x | x |
| PC aa C40:6 | x | x | x | x | x | x | x | x |
| PC aa C42:0 | x | x | x | x | x | x | x | x |
| PC aa C42:1 | x | x | x | x | x | x | x | x |
| PC aa C42:2 | x | x | x | x | x | x | x | x |
| PC aa C42:4 | x | x | x | x | x | x | x | x |
| PC aa C42:5 | x | x | x | x | x | x | x | x |
| PC aa C42:6 | x | x | x | x | x | x | x | x |
| PC ae C30:0 | x | x | x | x | x | x | x | x |
| PC ae C30:1 | - | x | x | x | x | x | x | x |
| PC ae C30:2 | x | x | x | x | x | x | x | x |
| PC ae C32:1 | - | x | x | x | x | x | x | x |
| PC ae C32:2 | - | x | x | x | x | x | x | x |
| PC ae C34:0 | - | x | x | x | x | x | x | x |
| PC ae C34:1 | - | x | x | x | x | x | x | x |
| PC ae C34:2 | - | x | x | x | x | x | x | x |
| PC ae C34:3 | - | x | x | x | x | x | x | x |
| PC ae C36:0 | - | x | x | x | x | x | x | x |
| PC ae C36:1 | - | x | x | x | x | x | x | x |
| PC ae C36:2 | - | x | x | x | x | x | x | x |
| PC ae C36:3 | - | x | x | x | x | x | x | x |
| PC ae C36:4 | - | x | x | x | x | x | x | x |
| PC ae C36:5 | - | x | x | x | x | x | x | x |
| PC ae C38:0 | - | x | x | x | x | x | x | x |
| PC ae C38:1 | - | x | x | x | x | x | x | x |
| PC ae C38:2 | - | x | x | x | x | x | x | x |
| PC ae C38:3 | - | x | x | x | x | x | x | x |
| PC ae C38:4 | - | x | x | x | x | x | x | x |
| PC ae C38:5 | - | x | x | x | x | x | x | x |
| PC.ae.C38.6 | - | x | x | x | x | x | x | x |
| PC ae C40:1 | - | x | x | x | x | x | x | x |
| PC ae C40:2 | x | x | x | x | x | x | x | x |
| PC ae C40:3 | - | x | x | x | x | x | x | x |
| PC ae C40:4 | - | x | x | x | x | x | x | x |
| PC ae C40:5 | - | x | x | x | x | x | x | x |
| PC ae C40:6 | - | x | x | x | x | x | x | x |
| PC ae C42:0 | - | x | x | x | x | x | x | x |
| PC ae C42:1 | x | x | x | x | x | x | x | x |
| PC ae C42:2 | x | x | x | x | x | x | x | x |
| PC ae C42:3 | x | x | x | x | - | x | x | x |
| PC ae C42:4 | x | x | x | x | x | - | x | x |
| PC ae C42:5 | x | x | x | x | x | x | x | x |
| PC ae C44:3 | x | x | x | x | x | x | x | x |
| PC ae C44:4 | x | x | x | x | x | x | x | x |
| PC ae C44:5 | x | x | x | x | x | x | x | x |
| PC ae C44:6 | x | x | x | x | x | x | x | x |
| lysoPC a C14:0 | x | x | x | x | x | x | x | x |
| lysoPC a C16:0 | x | x | x | x | x | x | x | x |
| lysoPC a C16:1 | x | x | x | x | x | x | x | x |
| lysoPC a C17:0 | x | x | x | x | x | x | x | x |
| lysoPC a C18:0 | x | x | x | x | x | x | x | x |
| lysoPC a C18:1 | x | x | x | x | x | x | x | x |
| lysoPC a C18:2 | x | x | x | x | x | x | x | x |
| lysoPC a C20:3 | x | x | x | x | x | x | x | x |
| lysoPC a C20:4 | x | x | x | x | x | x | x | x |
| lysoPC a C24:0 | x | x | x | x | x | x | x | x |
| lysoPC a C26:0 | - | x | x | x | x | x | x | x |
| lysoPC a C26:1 | - | x | x | x | x | x | x | x |
| lysoPC a C28:0 | - | x | x | x | x | x | x | x |
| lysoPC a C28:1 | - | x | x | x | x | x | x | x |
| SM (OH) C14:1 | x | x | x | x | x | x | x | x |
| SM (OH) C16:1 | x | x | x | x | x | x | x | x |
| SM (OH) C22:1 | - | x | x | x | x | x | x | x |
| SM (OH) C22:2 | - | x | x | x | x | - | x | x |
| SM (OH) C24:1 | - | x | x | x | x | x | x | x |
| SM C16:0 | x | x | x | x | x | x | x | x |
| SM C16:1 | x | x | x | x | x | x | x | x |
| SM C18:0 | x | x | x | x | x | x | x | x |
| SM C18:1 | x | x | x | x | x | x | x | x |
| SM C20:2 | x | x | x | x | x | - | - | x |
| SM C24:0 | - | x | x | x | x | x | x | x |
| SM C24:1 | - | x | x | x | x | x | x | x |
| SM C26:0 | - | x | - | - | x | - | x | - |
| SM C26:1 | - | - | - | - | x | - | x | - |
| TG (14:0_32:2) | x | - | - | - | - | - | - | - |
| TG (14:0_34:0) | x | - | - | - | - | - | - | - |
| TG (14:0_34:1) | x | - | - | - | - | - | - | - |
| TG (14:0_34:2) | x | - | - | - | - | - | - | - |
| TG (14:0_34:3) | x | - | - | - | - | - | - | - |
| TG (14:0_35:1) | x | - | - | - | - | - | - | - |
| TG (14:0_35:2) | x | - | - | - | - | - | - | - |
| TG (14:0_36:1) | x | - | - | - | - | - | - | - |
| TG (14:0_36:2) | x | - | - | - | - | - | - | - |
| TG (14:0_36:3) | x | - | - | - | - | - | - | - |
| TG (14:0_36:4) | x | - | - | - | - | - | - | - |
| TG (14:0_38:4) | x | - | - | - | - | - | - | - |
| TG (14:0_38:5) | x | - | - | - | - | - | - | - |
| TG (16:0_28:1) | x | - | - | - | - | - | - | - |
| TG (16:0_28:2) | x | - | - | - | - | - | - | - |
| TG (16:0_30:2) | x | - | - | - | - | - | - | - |
| TG (16:0_32:0) | x | - | - | - | - | - | - | - |
| TG (16:0_32:1) | x | - | - | - | - | - | - | - |
| TG (16:0_32:2) | x | - | - | - | - | - | - | - |
| TG (16:0_32:3) | x | - | - | - | - | - | - | - |
| TG (16:0_33:1) | x | - | - | - | - | - | - | - |
| TG (16:0_33:2) | x | - | - | - | - | - | - | - |
| TG (16:0_34:0) | x | - | - | - | - | - | - | - |
| TG (16:0_34:1) | x | - | - | - | - | - | - | - |
| TG (16:0_34:2) | x | - | - | - | - | - | - | - |
| TG (16:0_34:3) | x | - | - | - | - | - | - | - |
| TG (16:0_34:4) | x | - | - | - | - | - | - | - |
| TG (16:0_35:1) | x | - | - | - | - | - | - | - |
| TG (16:0_35:2) | x | - | - | - | - | - | - | - |
| TG (16:0_35:3) | x | - | - | - | - | - | - | - |
| TG (16:0_36:2) | x | - | - | - | - | - | - | - |
| TG (16:0_36:3) | x | - | - | - | - | - | - | - |
| TG (16:0_36:4) | x | - | - | - | - | - | - | - |
| TG (16:0_36:5) | x | - | - | - | - | - | - | - |
| TG (16:0_36:6) | x | - | - | - | - | - | - | - |
| TG (16:0_37:3) | x | - | - | - | - | - | - | - |
| TG (16:1_32:0) | x | - | - | - | - | - | - | - |
| TG (16:1_32:1) | x | - | - | - | - | - | - | - |
| TG (16:1_32:2) | x | - | - | - | - | - | - | - |
| TG (16:1_34:0) | x | - | - | - | - | - | - | - |
| TG (16:1_34:1) | x | - | - | - | - | - | - | - |
| TG (16:1_34:2) | x | - | - | - | - | - | - | - |
| TG (16:1_34:3) | x | - | - | - | - | - | - | - |
| TG (16:1_36:1) | x | - | - | - | - | - | - | - |
| TG (16:1_36:2) | x | - | - | - | - | - | - | - |
| TG (16:1_36:3) | x | - | - | - | - | - | - | - |
| TG (16:1_36:4) | x | - | - | - | - | - | - | - |
| TG (16:1_36:5) | x | - | - | - | - | - | - | - |
| TG (16:1_38:4) | x | - | - | - | - | - | - | - |
| TG (16:1_38:5) | x | - | - | - | - | - | - | - |
| TG (17:0_32:1) | x | - | - | - | - | - | - | - |
| TG (17:0_34:1) | x | - | - | - | - | - | - | - |
| TG (17:0_34:2) | x | - | - | - | - | - | - | - |
| TG (17:0_34:3) | x | - | - | - | - | - | - | - |
| TG (17:0_36:3) | x | - | - | - | - | - | - | - |
| TG (17:0_36:4) | x | - | - | - | - | - | - | - |
| TG (17:1_32:1) | x | - | - | - | - | - | - | - |
| TG (17:1_34:1) | x | - | - | - | - | - | - | - |
| TG (17:1_34:2) | x | - | - | - | - | - | - | - |
| TG (17:1_34:3) | x | - | - | - | - | - | - | - |
| TG (17:1_36:3) | x | - | - | - | - | - | - | - |
| TG (17:1_36:4) | x | - | - | - | - | - | - | - |
| TG (17:1_36:5) | x | - | - | - | - | - | - | - |
| TG (17:2_34:2) | x | - | - | - | - | - | - | - |
| TG (17:2_34:3) | x | - | - | - | - | - | - | - |
| TG (17:2_36:2) | x | - | - | - | - | - | - | - |
| TG (17:2_36:3) | x | - | - | - | - | - | - | - |
| TG (18:0_30:0) | x | - | - | - | - | - | - | - |
| TG (18:0_30:1) | x | - | - | - | - | - | - | - |
| TG (18:0_32:0) | x | - | - | - | - | - | - | - |
| TG (18:0_32:1) | x | - | - | - | - | - | - | - |
| TG (18:0_32:2) | x | - | - | - | - | - | - | - |
| TG (18:0_34:2) | x | - | - | - | - | - | - | - |
| TG (18:0_34:3) | x | - | - | - | - | - | - | - |
| TG (18:0_36:1) | x | - | - | - | - | - | - | - |
| TG (18:0_36:5) | x | - | - | - | - | - | - | - |
| TG (18:1_26:0) | x | - | - | - | - | - | - | - |
| TG (18:1_28:1) | x | - | - | - | - | - | - | - |
| TG (18:1_30:0) | x | - | - | - | - | - | - | - |
| TG (18:1_30:1) | x | - | - | - | - | - | - | - |
| TG (18:1_30:2) | x | - | - | - | - | - | - | - |
| TG (18:1_31:0) | x | - | - | - | - | - | - | - |
| TG (18:1_32:0) | x | - | - | - | - | - | - | - |
| TG (18:1_32:1) | x | - | - | - | - | - | - | - |
| TG (18:1_32:2) | x | - | - | - | - | - | - | - |
| TG (18:1_32:3) | x | - | - | - | - | - | - | - |
| TG (18:1_33:0) | x | - | - | - | - | - | - | - |
| TG (18:1_33:1) | x | - | - | - | - | - | - | - |
| TG (18:1_33:2) | x | - | - | - | - | - | - | - |
| TG (18:1_33:3) | x | - | - | - | - | - | - | - |
| TG (18:1_34:1) | x | - | - | - | - | - | - | - |
| TG (18:1_34:2) | x | - | - | - | - | - | - | - |
| TG (18:1_34:3) | x | - | - | - | - | - | - | - |
| TG (18:1_34:4) | x | - | - | - | - | - | - | - |
| TG (18:1_35:2) | x | - | - | - | - | - | - | - |
| TG (18:1_35:3) | x | - | - | - | - | - | - | - |
| TG (18:1_36:4) | x | - | - | - | - | - | - | - |
| TG (18:1_36:5) | x | - | - | - | - | - | - | - |
| TG (18:1_36:6) | x | - | - | - | - | - | - | - |
| TG (18:1_38:7) | x | - | - | - | - | - | - | - |
| TG (18:2_28:0) | x | - | - | - | - | - | - | - |
| TG (18:2_30:0) | x | - | - | - | - | - | - | - |
| TG (18:2_30:1) | x | - | - | - | - | - | - | - |
| TG (18:2_31:0) | x | - | - | - | - | - | - | - |
| TG (18:2_32:0) | x | - | - | - | - | - | - | - |
| TG (18:2_32:1) | x | - | - | - | - | - | - | - |
| TG (18:2_32:2) | x | - | - | - | - | - | - | - |
| TG (18:2_33:0) | x | - | - | - | - | - | - | - |
| TG (18:2_33:1) | x | - | - | - | - | - | - | - |
| TG (18:2_33:2) | x | - | - | - | - | - | - | - |
| TG (18:2_34:0) | x | - | - | - | - | - | - | - |
| TG (18:2_34:1) | x | - | - | - | - | - | - | - |
| TG (18:2_34:2) | x | - | - | - | - | - | - | - |
| TG (18:2_34:3) | x | - | - | - | - | - | - | - |
| TG (18:2_34:4) | x | - | - | - | - | - | - | - |
| TG (18:2_35:1) | x | - | - | - | - | - | - | - |
| TG (18:2_35:2) | x | - | - | - | - | - | - | - |
| TG (18:2_35:3) | x | - | - | - | - | - | - | - |
| TG (18:2_36:3) | x | - | - | - | - | - | - | - |
| TG (18:2_36:4) | x | - | - | - | - | - | - | - |
| TG (18:2_36:5) | x | - | - | - | - | - | - | - |
| TG (18:3_30:0) | x | - | - | - | - | - | - | - |
| TG (18:3_32:0) | x | - | - | - | - | - | - | - |
| TG (18:3_32:1) | x | - | - | - | - | - | - | - |
| TG (18:3_34:0) | x | - | - | - | - | - | - | - |
| TG (18:3_34:1) | x | - | - | - | - | - | - | - |
| TG (18:3_34:2) | x | - | - | - | - | - | - | - |
| TG (18:3_34:3) | x | - | - | - | - | - | - | - |
| TG (18:3_36:2) | x | - | - | - | - | - | - | - |
| TG (18:3_36:3) | x | - | - | - | - | - | - | - |
| TG (18:3_36:4) | x | - | - | - | - | - | - | - |
| TG (20:0_32:3) | x | - | - | - | - | - | - | - |
| TG (20:0_32:4) | x | - | - | - | - | - | - | - |
| TG (20:1_30:1) | x | - | - | - | - | - | - | - |
| TG (20:1_32:1) | x | - | - | - | - | - | - | - |
| TG (20:1_32:2) | x | - | - | - | - | - | - | - |
| TG (20:1_32:3) | x | - | - | - | - | - | - | - |
| TG (20:1_34:2) | x | - | - | - | - | - | - | - |
| TG (20:2_32:0) | x | - | - | - | - | - | - | - |
| TG (20:2_32:1) | x | - | - | - | - | - | - | - |
| TG (20:3_32:0) | x | - | - | - | - | - | - | - |
| TG (20:3_32:1) | x | - | - | - | - | - | - | - |
| TG (20:3_32:2) | x | - | - | - | - | - | - | - |
| TG (20:3_34:2) | x | - | - | - | - | - | - | - |
| TG (20:4_30:0) | x | - | - | - | - | - | - | - |
| TG (20:4_32:0) | x | - | - | - | - | - | - | - |
| TG (20:4_32:1) | x | - | - | - | - | - | - | - |
| TG (20:4_32:2) | x | - | - | - | - | - | - | - |
| TG (20:4_33:2) | x | - | - | - | - | - | - | - |
| TG (20:4_34:2) | x | - | - | - | - | - | - | - |
| TG (20:4_34:3) | x | - | - | - | - | - | - | - |
| TG (20:4_36:4) | x | - | - | - | - | - | - | - |
| TG (20:4_36:5) | x | - | - | - | - | - | - | - |
| TG (20:5_34:1) | x | - | - | - | - | - | - | - |
| TG (20:5_34:2) | x | - | - | - | - | - | - | - |
| TG (20:5_36:3) | x | - | - | - | - | - | - | - |
| TG (22:5_34:3) | x | - | - | - | - | - | - | - |
| TG (22:6_32:1) | x | - | - | - | - | - | - | - |
| TG (22:6_34:3) | x | - | - | - | - | - | - | - |

The table lists metabolites analyzed in human serum and seven murine tissues: plasma, liver, adrenal gland, adipose tissue, testis, lung, and cerebellum. A sign of ‘x’ indicates that the metabolite was utilized, while ‘-’ indicates it was not. A total of 303 metabolites were used in human serum, compared to 178, 178, 176, 171, 173, 173, and 174 metabolites in murine plasma, liver, adrenal gland, adipose tissue, testis, lung, and cerebellum, respectively.

*: An analytical differentiation of isobaric/isomeric metabolites was not possible. The metabolite synonyms (or names) represent a set of isomeric and isobaric compounds.

## Table S4. Ten significant serum metabolites in COMBI vs. MET-T2D

|  | COMBI-T2D vs. MET-T2D | | COMBI-T2D vs. ndt-T2D | | ndt-T2D vs. NGT | |
| --- | --- | --- | --- | --- | --- | --- |
| Metabolites | β | *P*-values | β | *P*-values | β | *P*-values |
| C2 | 0.49 | **0.025** | 0.72 | **2.72x10^-03^** | 0.15 | 0.218 |
| Arg | -0.49 | **0.020** | -1.03 | **1.55x10^-05^** | -0.14 | 0.253 |
| Ile | 0.71 | **4.13x10^-04^** | 1.01 | **2.99x10^-06^** | 0.18 | 0.067 |
| Leu | 0.75 | **2.63x10^-04^** | 1.03 | **3.22x10^-06^** | 0.06 | 0.561 |
| Thr | -0.43 | **0.043** | -0.33 | 0.182 | 0.04 | 0.770 |
| Val | 0.63 | **3.26x10^-03^** | 0.97 | **2.81x10^-05^** | 0.16 | 0.131 |
| 3-IAA | 0.46 | **0.040** | 0.47 | 0.064 | 0.19 | 0.121 |
| DCA | 0.46 | **0.043** | 0.59 | **0.015** | 0.32 | **0.008** |
| HexCer (d16:1/22:0) | 0.42 | **0.048** | 0.29 | 0.234 | -0.20 | 0.093 |
| TG (20:4_36:4) | 0.48 | **0.028** | 0.31 | 0.193 | -0.01 | 0.962 |

The table shows results from multivariable linear regression analyses (β and *P*-values) adjusted for age, sex, BMI, smoking, alcohol, physical activity, HbA_1C_, and HDL cholesterol across pairwise comparisons: COMBI-T2D vs. MET-T2D, COMBI-T2D vs. ndt-T2D, and ndt-T2D vs. NGT. A *P*‑value < 0.05 was considered statistically significant and is shown in bold. Abbreviations: NGT, normal glucose tolerance; ndt-T2D, non-antidiabetic drug treated type 2 diabetes; MET-T2D, metformin-treated type 2 diabetes; COMBI-T2D, Sodium-glucose-cotransporter-2-inhibitor and metformin-treated type 2 diabetes.

## Table S5. 82 significant plasma metabolites in COMBI vs. MET-db/db

|  | COMBI-db/db vs. MET-db/db | | COMBI-db/db vs. VG-db/db | | VG-db/db vs. WT | |
| --- | --- | --- | --- | --- | --- | --- |
| Metabolites | β | *P*-values | β | *P*-values | β | *P*-values |
| H1 | -1.55 | **2.95x10^-05^** | -1.76 | **1.98x10^-08^** | 1.89 | **2.94x10^-12^** |
| C0 | -1.54 | **3.29x10^-05^** | -1.31 | **1.18x10^-03^** | -1.44 | **1.84x10^-04^** |
| C2 | 0.87 | **0.049** | 0.59 | 0.196 | -1.61 | **7.94x10^-06^** |
| C3 | -1.24 | **2.59x10^-03^** | 0.35 | 0.447 | -1.07 | **0.012** |
| C3:1 | 0.95 | **0.030** | 0.25 | 0.595 | 0.83 | 0.060 |
| C3-DC (C4-OH) | 1.43 | **2.31x10^-04^** | 1.47 | **1.25x10^-04^** | -0.92 | **0.036** |
| C4:1 | -0.97 | **0.026** | -1.35 | **7.61x10^-04^** | 1.70 | **6.28x10^-07^** |
| C5-DC (C6-OH) | 1.06 | **0.013** | 1.18 | **4.64x10^-03^** | -1.46 | **1.45x10^-04^** |
| C7-DC | 1.23 | **2.76x10^-03^** | 1.28 | **1.62x10^-03^** | -0.42 | 0.359 |
| C10 | -1.12 | **8.32x10^-03^** | -1.31 | **1.20x10^-03^** | 1.68 | **9.92x10^-07^** |
| C14 | 1.13 | **7.17x10^-03^** | 1.31 | **1.15x10^-03^** | -0.95 | **0.030** |
| C14:1 | 1.12 | **7.93x10^-03^** | 1.10 | **9.19x10^-03^** | 0.06 | 0.904 |
| C16 | 1.16 | **5.82x10^-03^** | 1.28 | **1.63x10^-03^** | -1.27 | **0.002** |
| C16:1 | 0.97 | **0.025** | 1.31 | **1.11x10^-03^** | 0.83 | 0.060 |
| C16:1-OH | 1.05 | **0.015** | 1.42 | **2.61x10^-04^** | -0.48 | 0.291 |
| C16:2 | 1.12 | **8.03x10^-03^** | 1.24 | **2.53x10^-03^** | -0.27 | 0.563 |
| C18 | 1.20 | **3.74x10^-03^** | 0.86 | 0.053 | 0.22 | 0.638 |
| C18:1 | 1.51 | **6.56x10^-05^** | 1.27 | **1.91x10^-03^** | 0.23 | 0.620 |
| C18:1-OH | 1.09 | **0.010** | 1.52 | **4.92x10^-05^** | 0.09 | 0.849 |
| C18:2 | 1.18 | **4.51x10^-03^** | 0.98 | **0.023** | 0.23 | 0.613 |
| Ala | -1.31 | **1.14x10^-03^** | -0.39 | 0.392 | 0.33 | 0.477 |
| Asn | -0.96 | **0.028** | -1.04 | **0.015** | -0.52 | 0.255 |
| Lys | -1.04 | **0.015** | -1.11 | **8.52x10^-03^** | -1.30 | **1.36x10^-03^** |
| Met | -1.48 | **1.07x10^-04^** | -1.34 | **8.35x10^-04^** | -1.11 | **0.009** |
| Pro | -1.55 | **2.89x10^-05^** | -1.44 | **2.07x10^-04^** | 0.72 | 0.111 |
| Ser | -1.34 | **8.21x10^-04^** | -1.43 | **2.19x10^-04^** | 0.10 | 0.828 |
| Thr | -1.25 | **2.31x10^-03^** | -1.50 | **7.12x10^-05^** | -0.36 | 0.435 |
| Tyr | -1.14 | **6.98x10^-03^** | -1.34 | **7.82x10^-04^** | 1.37 | **0.001** |
| Putrescine | 1.07 | **0.013** | 0.91 | **0.039** | -1.72 | **2.50x10^-07^** |
| Sarcosine | 0.90 | **0.042** | -0.80 | 0.073 | 0.35 | 0.450 |
| Spermidine | 1.18 | **4.55x10^-03^** | 1.24 | **2.49x10^-03^** | -1.12 | **0.008** |
| Met-SO | -1.40 | **3.47x10^-04^** | -0.90 | **0.041** | -0.51 | 0.269 |
| PC aa C24:0 | 1.01 | **0.019** | 0.30 | 0.510 | 0.85 | 0.054 |
| PC aa C32:0 | 1.26 | **2.03x10^-03^** | 0.61 | **0.178** | -1.41 | **3.26x10^-04^** |
| PC aa C32:3 | 1.02 | **0.018** | 0.28 | **0.550** | 0.99 | **0.023** |
| PC aa C34:2 | 1.23 | **2.97x10^-03^** | -0.18 | **0.704** | 1.09 | **0.010** |
| PC aa C36:2 | 0.88 | **0.045** | -0.24 | **0.609** | 1.62 | **5.11x10^-06^** |
| PC aa C38:0 | 1.29 | **1.53x10^-03^** | 0.77 | **0.086** | 0.16 | 0.736 |
| PC aa C38:1 | 1.08 | **0.011** | 0.73 | **0.105** | 0.76 | 0.091 |
| PC.aa.C40.1 | 1.06 | **0.013** | 0.62 | **0.171** | -0.29 | 0.531 |
| PC aa C40:2 | 1.14 | **7.04x10^-03^** | 0.29 | 0.538 | -0.17 | 0.714 |
| PC aa C42:0 | 1.08 | **0.012** | 0.53 | 0.243 | 0.81 | 0.070 |
| PC aa C42:1 | 1.31 | **1.23x10^-03^** | 0.87 | **0.047** | -0.36 | 0.442 |
| PC aa C42:2 | 0.95 | **0.029** | 0.14 | 0.766 | -0.58 | 0.199 |
| PC aa C42:4 | 1.05 | **0.015** | 0.64 | 0.158 | -1.36 | **6.14x10^-04^** |
| PC ae C30:0 | 1.21 | **3.64x10^-03^** | 0.37 | 0.419 | -0.53 | 0.249 |
| PC ae C30:2 | 1.12 | **8.27x10^-03^** | 0.23 | 0.622 | 0.30 | 0.520 |
| PC ae C32:1 | 1.00 | **0.020** | 0.16 | 0.737 | -0.92 | **0.036** |
| PC ae C32:2 | 1.02 | **0.018** | -0.14 | 0.772 | -1.26 | **2.07x10^-03^** |
| PC ae C34:1 | 1.13 | **7.58x10^-03^** | 0.64 | 0.157 | -1.62 | **5.56x10^-06^** |
| PC.ae.C34.2 | 1.06 | **0.013** | 0.08 | 0.866 | -1.37 | **5.86x10^-04^** |
| PC ae C36:1 | 1.00 | **0.020** | 0.08 | 0.864 | -0.65 | 0.149 |
| PC ae C36:2 | 1.13 | **7.21x10^-03^** | -0.51 | 0.266 | 0.91 | **0.039** |
| PC.ae.C36.3 | 1.06 | **0.013** | -0.03 | 0.945 | -0.62 | 0.175 |
| PC ae C36:4 | 1.21 | **3.46x10^-03^** | 0.44 | 0.344 | -0.94 | **0.032** |
| PC ae C38:2 | 1.41 | **3.13x10^-04^** | -0.31 | 0.505 | -0.73 | 0.104 |
| PC ae C38:3 | 0.92 | **0.036** | -0.04 | 0.927 | 1.47 | **1.24x10^-04^** |
| PC ae C38:4 | 1.04 | **0.015** | -0.08 | 0.857 | 0.05 | 0.906 |
| PC ae C38:5 | 1.24 | **2.66x10^-03^** | 0.59 | 0.192 | -0.83 | 0.060 |
| PC ae C38:6 | 0.97 | **0.026** | 0.49 | 0.288 | -0.27 | 0.555 |
| PC ae C40:2 | 0.87 | **0.048** | 0.19 | 0.681 | 0.81 | 0.070 |
| PC ae C40:3 | 0.91 | **0.039** | 0.38 | 0.414 | 1.20 | **4.00x10^-03^** |
| PC ae C40:4 | 1.27 | **1.80x10^-03^** | -0.10 | 0.824 | -0.79 | 0.075 |
| PC ae C40:5 | 1.15 | **6.36x10^-03^** | 0.54 | 0.239 | -0.92 | **0.036** |
| PC ae C40:6 | 1.18 | **4.61x10^-03^** | 0.42 | 0.356 | -0.58 | 0.205 |
| PC ae C42:0 | 0.98 | **0.024** | 0.60 | 0.186 | -1.29 | **1.43x10^-03^** |
| PC ae C42:1 | 1.09 | **0.011** | 0.64 | 0.157 | 0.09 | 0.849 |
| PC ae C42:2 | 0.94 | **0.031** | 0.38 | 0.414 | -0.74 | 0.098 |
| PC ae C42:5 | 1.20 | **3.90x10^-03^** | 0.49 | 0.286 | -1.00 | **0.020** |
| PC ae C44:4 | 0.97 | **0.026** | 0.02 | **0.958** | 0.64 | 0.160 |
| PC ae C44:5 | 1.08 | **0.011** | 0.65 | 0.151 | -0.99 | **0.023** |
| PC ae C44:6 | 0.91 | **0.038** | 0.21 | 0.644 | -0.60 | 0.190 |
| LysoPC a C14:0 | -1.16 | **5.42x10^-03^** | -1.25 | **2.25x10^-03^** | -1.17 | **0.005** |
| LysoPC a C18:1 | -1.06 | **0.014** | -1.09 | **0.010** | 0.58 | 0.202 |
| LysoPC a C18:2 | -0.99 | **0.022** | -1.08 | **0.012** | 1.18 | **4.72x10^-03^** |
| LysoPC a C24:0 | 1.12 | **8.31x10^-03^** | 0.26 | 0.582 | -0.28 | 0.548 |
| LysoPC a C26:0 | 1.03 | **0.017** | 0.26 | **0.572** | 0.29 | 0.528 |
| LysoPC a C28:0 | 1.01 | **0.020** | 0.07 | **0.879** | 1.28 | **1.61x10^-03^** |
| SM C16:1 | 1.14 | **6.60x10^-03^** | 0.74 | 0.097 | 0.82 | 0.064 |
| SM C24:0 | 1.20 | **3.84x10^-03^** | 0.63 | 0.163 | -0.15 | 0.742 |
| SM (OH) C22:1 | 1.00 | **0.020** | 0.65 | 0.153 | 0.39 | 0.402 |
| SM (OH) C22:2 | 1.24 | **2.67x10^-03^** | 0.47 | 0.309 | -1.09 | **0.010** |
|  |  |  |  |  |  |  |

The table shows results from univariate linear regression analyses (β and *P*-values) in three pairwise comparisons: COMBI-db/db vs. MET-db/db, COMBI-db/db vs. VG-db/db, and VG-db/db vs. WT. A *P*‑value < 0.05 was considered statistically significant and is shown in bold. **Abbreviations:** WT, wild type mice; VG-db/db, vehicle‑gavaged diabetic mice; MET-db/db, metformin-treated diabetic mice; COMBI-db/db, Sodium-glucose-cotransporter-2-inhibitor and metformin-treated diabetic mice.

## Table S6. 52 significant liver metabolites in COMBI vs. MET-db/db

|  | COMBI-db/db vs. MET-db/db | | COMBI-db/db vs. VG-db/db | | VG-db/db vs. WT | |
| --- | --- | --- | --- | --- | --- | --- |
| Metabolites | β | *P*-values | β | *P*-values | β | *P*-values |
| H1 | -1.21 | **3.56x10^-03^** | -0.13 | **0.772** | 0.74 | 0.096 |
| C3-DC (C4-OH) | 1.26 | **2.03x10^-03^** | 1.22 | **3.03x10^-03^** | 0.57 | 0.212 |
| C4:1 | -1.00 | **0.020** | 0.14 | 0.771 | 0.47 | 0.308 |
| C5-DC (C6-OH) | 1.22 | **3.31x10^-03^** | 1.31 | **1.21x10^-03^** | -1.12 | **8.02x10^-03^** |
| Ala | -0.88 | **0.047** | 0.08 | **0.860** | -0.55 | 0.229 |
| Cit | 1.36 | **6.38x10^-04^** | -0.17 | **0.717** | -1.29 | **1.47x10^-03^** |
| Gln | -0.98 | **0.023** | 0.27 | 0.562 | -0.69 | 0.125 |
| Gly | 0.88 | **0.047** | 0.64 | 0.153 | -1.76 | **5.23x10^-08^** |
| His | -0.93 | **0.034** | -0.81 | 0.068 | -0.75 | 0.092 |
| Ile | 1.07 | **0.012** | 1.25 | **2.32x10^-03^** | 0.20 | 0.661 |
| Leu | 1.10 | **9.60x10^-03^** | 0.71 | 0.113 | 0.17 | 0.719 |
| Orn | 1.13 | **7.30x10^-03^** | 0.99 | **0.022** | -1.60 | **8.51x10^-06^** |
| Thr | 1.19 | **4.46x10^-03^** | 0.83 | 0.063 | -0.95 | **0.029** |
| Tyr | 0.89 | **0.044** | 0.63 | 0.162 | -0.33 | 0.481 |
| Val | 1.02 | **0.018** | 0.94 | **0.031** | 0.71 | 0.114 |
| alpha-AAA | 1.13 | **7.21x10^-03^** | 0.50 | 0.278 | -0.97 | **0.026** |
| Ac-Orn | 1.03 | **0.017** | 0.46 | 0.317 | -1.56 | **2.49x10^-05^** |
| ADMA | 1.27 | **1.91x10^-03^** | 0.25 | 0.593 | -1.34 | **7.99x10^-04^** |
| Serotonin | 1.02 | **0.018** | 1.24 | **2.64x10^-03^** | -0.95 | **0.030** |
| PC aa C30:0 | 1.00 | **0.021** | 0.51 | 0.264 | 0.67 | 0.136 |
| PC aa C32:0 | 0.90 | **0.040** | 0.47 | 0.303 | -0.41 | 0.376 |
| PC aa C34:1 | 0.99 | **0.023** | 0.88 | **0.045** | 0.34 | 0.461 |
| PC aa C34:2 | 0.98 | **0.024** | 0.89 | **0.044** | 0.06 | 0.906 |
| PC aa C36:4 | 1.20 | **3.88x10^-03^** | 0.94 | **0.032** | 1.16 | **5.82x10^-03^** |
| PC aa C38:0 | 0.94 | **0.031** | 0.58 | 0.203 | 0.09 | 0.848 |
| PC aa C38:4 | 0.90 | **0.041** | 0.43 | 0.353 | 1.72 | **3.09x10^-07^** |
| PC aa C38:6 | 1.01 | **0.020** | 0.57 | 0.213 | 1.26 | **2.12x10^-03^** |
| PC aa C40:1 | 1.04 | **0.015** | 0.66 | 0.144 | 0.09 | 0.847 |
| PC aa C40:4 | 0.89 | **0.042** | 0.58 | 0.200 | 0.56 | 0.221 |
| PC aa C40:5 | 0.87 | **0.048** | 0.80 | 0.071 | 0.03 | 0.946 |
| PC aa C42:1 | 1.01 | **0.020** | 0.35 | 0.448 | 0.07 | 0.883 |
| PC aa C42:5 | 0.95 | **0.030** | 0.56 | 0.219 | -0.71 | 0.113 |
| PC ae C34:0 | 0.92 | **0.035** | 0.41 | 0.376 | -0.93 | **0.033** |
| PC ae C36:2 | 0.88 | **0.046** | 0.46 | 0.311 | 0.08 | 0.869 |
| PC ae C36:3 | 1.00 | **0.020** | 0.46 | 0.315 | -1.31 | **1.20x10^-03^** |
| PC ae C36:4 | 1.13 | **7.24x10^-03^** | 0.90 | **0.040** | -1.59 | **1.08x10^-05^** |
| PC ae C36:5 | 1.21 | **3.42x10^-03^** | 1.13 | **7.48x10^-03^** | -0.11 | 0.813 |
| PC ae C38:4 | 1.09 | **0.010** | 0.64 | 0.160 | 0.16 | 0.728 |
| PC ae C38:5 | 1.10 | **9.19x10^-03^** | 0.72 | 0.108 | -1.17 | **5.22x10^-03^** |
| PC ae C38:6 | 1.00 | **0.021** | 0.88 | **0.046** | -1.34 | **8.20x10^-04^** |
| PC ae C40:3 | 0.87 | **0.049** | 0.28 | 0.542 | 0.95 | **0.029** |
| PC ae C40:4 | 1.20 | **3.84x10^-03^** | 0.37 | 0.422 | -0.74 | 0.101 |
| PC ae C40:5 | 1.16 | **5.70x10^-03^** | 0.33 | 0.469 | -0.98 | **0.023** |
| PC ae C40:6 | 1.02 | **0.017** | 0.62 | 0.169 | 0.47 | 0.302 |
| PC ae C44:6 | 0.97 | **0.026** | -0.26 | 0.568 | 0.91 | **0.037** |
| SM C16:0 | 0.91 | **0.037** | 0.54 | 0.238 | 1.60 | **9.13x10^-06^** |
| SM C16:1 | 1.01 | **0.019** | 0.84 | 0.057 | 0.30 | 0.518 |
| SM C18:1 | 1.01 | **0.019** | 0.71 | 0.115 | 1.60 | **9.47x10^-06^** |
| SM C24:1 | 0.87 | **0.049** | 0.41 | 0.379 | -0.41 | 0.379 |
| SM (OH) C14:1 | 0.87 | **0.048** | 0.54 | 0.237 | 1.11 | **9.15x10^-03^** |
| SM (OH) C22:2 | 1.14 | **6.98x10^-03^** | 0.02 | 0.967 | 0.60 | 0.183 |
| SM (OH) C24:1 | 0.92 | **0.037** | 0.45 | 0.333 | 0.17 | 0.715 |

The table shows results from univariate linear regression analyses (β and P-values) in three pairwise comparisons: COMBI-db/db vs. MET-db/db, COMBI-db/db vs. VG-db/db, and VG-db/db vs. WT. A *P*‑value < 0.05 was considered statistically significant and is shown in bold. **Abbreviations:** WT, wild type mice; VG-db/db, vehicle‑gavaged diabetic mice; MET-db/db, metformin-treated diabetic mice; COMBI-db/db, Sodium-glucose-cotransporter-2-inhibitor and metformin-treated diabetic mice.

## Table S7. 30 significant adrenal gland metabolites in COMBI vs. MET-db/db

|  | COMBI-db/db vs. MET-db/db | | COMBI-db/db vs. VG-db/db | | VG-db/db vs. WT | |
| --- | --- | --- | --- | --- | --- | --- |
| Metabolites | β | *P*-values | β | *P*-values | β | *P*-values |
| C3 | 1.03 | **0.017** | 0.35 | 0.451 | -0.34 | **0.040** |
| C3-DC (C4-OH) | 1.51 | **5.89x10^-05^** | 1.43 | **2.47x10^-04^** | 0.18 | 0.400 |
| C5 | 0.91 | **0.037** | -0.05 | 0.912 | 0.17 | 0.314 |
| C5-DC (C6-OH) | 1.29 | **1.49x10^-03^** | 1.04 | **0.016** | 0.27 | 0.126 |
| C9 | 1.23 | **2.90x10^-03^** | 0.90 | **0.041** | 0.14 | 0.473 |
| C10 | 0.95 | **0.030** | 0.58 | 0.206 | 0.11 | 0.439 |
| C14:2 | 1.17 | **5.24x10^-03^** | 0.70 | 0.118 | -0.30 | 0.167 |
| Sarcosine | 0.90 | **0.041** | 0.84 | 0.058 | -0.27 | 0.096 |
| PC aa C24:0 | 1.19 | **4.24x10^-03^** | 0.46 | 0.312 | 0.41 | **1.18x10^-04^** |
| PC aa C28:1 | 1.12 | **8.05x10^-03^** | 0.70 | 0.121 | 0.46 | **1.45x10^-05^** |
| PC aa C32:2 | 0.91 | **0.039** | 0.87 | **0.048** | -0.39 | **5.50x10^-04^** |
| PC aa C34:3 | 1.07 | **0.012** | 1.12 | **8.36x10^-03^** | -0.16 | 0.073 |
| PC aa C34:4 | 1.07 | **0.012** | 0.91 | **0.038** | 0.06 | 0.511 |
| PC aa C36:5 | 1.03 | **0.016** | 0.76 | 0.089 | 0.41 | **1.12x10^-03^** |
| PC aa C40:6 | 1.00 | **0.021** | 1.23 | **2.94x10^-03^** | 0.24 | 0.081 |
| PC aa C42:1 | 0.91 | **0.037** | 0.98 | **0.024** | -0.15 | 0.323 |
| PC aa C42:6 | 0.87 | **0.048** | 1.07 | **0.012** | 0.13 | 0.282 |
| PC ae C38:0 | 0.98 | **0.024** | 0.69 | 0.125 | 0.28 | **0.005** |
| PC ae C38:2 | 0.99 | **0.022** | 0.79 | 0.078 | -0.56 | **3.55x10^-06^** |
| PC ae C40:2 | 0.98 | **0.024** | 0.84 | 0.058 | 0.13 | 0.277 |
| PC ae C42:0 | 0.88 | **0.047** | 1.16 | **5.41x10^-03^** | 0.22 | 0.053 |
| PC ae C44:3 | 1.16 | **5.55x10^-03^** | 1.45 | **1.81x10^-04^** | 0.34 | **0.026** |
| LysoPC a C20:4 | 0.87 | **0.049** | 0.38 | 0.410 | 0.36 | **0.034** |
| LysoPC a C26:0 | 1.16 | **5.40x10^-03^** | 0.94 | **0.032** | 0.04 | 0.765 |
| SM C16:1 | 0.98 | **0.024** | 0.92 | **0.036** | 0.15 | 0.127 |
| SM C18:1 | 1.07 | **0.013** | 1.12 | **8.11x10^-03^** | 0.02 | 0.874 |
| SM C24:1 | 0.87 | **0.047** | 1.06 | **0.013** | -0.06 | 0.531 |
| SM (OH) C14:1 | 1.17 | **5.04x10^-03^** | 1.14 | **6.51x10^-03^** | 0.14 | 0.247 |
| SM (OH) C16:1 | 0.99 | **0.022** | 0.67 | 0.139 | -0.09 | 0.465 |
| SM (OH) C22:2 | 1.06 | **0.014** | 1.02 | **0.018** | 0.08 | 0.367 |

The table shows results from univariate linear regression analyses (β and *P*-values) in three pairwise comparisons: COMBI-db/db vs. MET-db/db, COMBI-db/db vs. VG-db/db, and VG-db/db vs. WT. A *P*‑value < 0.05 was considered statistically significant and is shown in bold. **Abbreviations:** WT, wild type mice; VG-db/db, vehicle‑gavaged diabetic mice; MET-db/db, metformin-treated diabetic mice; COMBI-db/db, Sodium-glucose-cotransporter-2-inhibitor and metformin-treated diabetic mice.

## Table S8. 12 significant adipose tissue metabolites in COMBI vs. MET-db/db

|  | COMBI-db/db vs. MET-db/db | | COMBI-db/db vs. VG-db/db | | VG-db/db vs. WT | |
| --- | --- | --- | --- | --- | --- | --- |
| Metabolites | β | *P*-values | β | *P*-values | β | *P*-values |
| H1 | -0.94 | **0.031** | -1.27 | **1.76x10^-03^** | 1.18 | **4.71x10^-03^** |
| C0 | -1.28 | **1.71x10^-03^** | -0.31 | 0.498 | -1.18 | **4.76x10^-03^** |
| C3-DC (C4-OH) | 1.27 | **1.76x10^-03^** | 1.15 | **6.06x10^-03^** | 1.06 | **0.014** |
| C18 | 1.05 | **0.015** | 0.76 | 0.089 | -1.16 | **5.72x10^-03^** |
| Ala | -0.90 | **0.041** | -0.35 | 0.450 | 0.26 | 0.579 |
| Glu | 1.06 | **0.013** | 0.71 | 0.116 | 0.55 | 0.230 |
| Lys | -1.08 | **0.011** | -0.10 | 0.830 | -1.67 | **1.59x10^-06^** |
| Putrescine | -1.03 | **0.017** | -0.53 | 0.246 | -1.05 | **0.014** |
| Taurine | -0.95 | **0.030** | 0.31 | 0.508 | -0.68 | 0.133 |
| PC aa C42:1 | 0.93 | **0.034** | 0.23 | 0.621 | -1.14 | **6.77x10^-03^** |
| LysoPC a C18:2 | -1.05 | **0.014** | -0.97 | **0.025** | -1.39 | **4.31x10^-04^** |
| LysoPC a C26:0 | 0.89 | **0.043** | 0.73 | 0.102 | -0.16 | 0.734 |

The table shows results from univariate linear regression analyses (β and *P*-values) in three pairwise comparisons: COMBI-db/db vs. MET-db/db, COMBI-db/db vs. VG-db/db, and VG-db/db vs. WT. A *P*‑value < 0.05 was considered statistically significant and is shown in bold. **Abbreviations:** WT, wild type mice; VG-db/db, vehicle‑gavaged diabetic mice; MET-db/db, metformin-treated diabetic mice; COMBI-db/db, Sodium-glucose-cotransporter-2-inhibitor and metformin-treated diabetic mice.

## Table S9. Seven significant testis metabolites in COMBI vs. MET-db/db

|  | COMBI-db/db vs. MET-db/db | | COMBI-db/db vs. VG-db/db | | VG-db/db vs. WT | |
| --- | --- | --- | --- | --- | --- | --- |
| Metabolites | β | *P*-values | β | *P*-values | β | *P*-values |
| H1 | -1.35 | **7.29x10^-04^** | -1.64 | **2.91x10^-06^** | 1.88 | **4.52x10^-12^** |
| C3-DC (C4-OH) | 1.17 | **5.31x10^-03^** | 0.67 | 0.139 | 0.25 | 0.590 |
| C4:1 | 1.11 | **8.50x10^-03^** | 0.31 | 0.507 | 0.86 | 0.052 |
| C5-DC (C6-OH) | 1.00 | **0.021** | 0.48 | 0.298 | 0.62 | 0.174 |
| Gln | 0.88 | **0.044** | 0.90 | **0.040** | 0.22 | 0.633 |
| LysoPC a C18:2 | -1.00 | **0.021** | -1.39 | **4.27x10^-04^** | 0.88 | **0.046** |
| LysoPC a C26:1 | 0.94 | **0.032** | 0.52 | 0.259 | 0.26 | 0.573 |

The table shows results from univariate linear regression analyses (β and *P*-values) in three pairwise comparisons: COMBI-db/db vs. MET-db/db, COMBI-db/db vs. VG-db/db, and VG-db/db vs. WT. A *P*‑value < 0.05 was considered statistically significant and is shown in bold. **Abbreviations:** WT, wild type mice; VG-db/db, vehicle‑gavaged diabetic mice; MET-db/db, metformin-treated diabetic mice; COMBI-db/db, Sodium-glucose-cotransporter-2-inhibitor and metformin-treated diabetic mice.

## Table S10. Seven significant lung metabolites in COMBI vs. MET-db/db

|  | COMBI-db/db vs. MET-db/db | | COMBI-db/db vs. VG-db/db | | VG-db/db vs. WT | |
| --- | --- | --- | --- | --- | --- | --- |
| Metabolites | β | *P*-values | β | *P*-values | β | *P*-values |
| H1 | -1.44 | **1.97x10^-04^** | -1.69 | **8.61x10^-07^** | 1.90 | **5.70x10^-13^** |
| C3:1 | 1.29 | **1.54x10^-03^** | 1.16 | **5.55x10^-03^** | -0.28 | 0.539 |
| C6 (C4:1-DC) | -0.88 | **0.046** | -0.53 | 0.249 | -1.69 | **8.54x10^-07^** |
| C10 | -0.89 | **0.042** | -0.59 | 0.191 | -1.23 | **2.87x10^-03^** |
| Ile | 1.25 | **2.42x10^-03^** | 1.13 | **7.52x10^-03^** | 0.42 | 0.360 |
| Leu | 1.27 | **1.77x10^-03^** | 0.39 | 0.398 | 0.57 | 0.210 |
| PC ae C30:2 | 0.98 | **0.025** | 0.19 | 0.686 | -0.09 | 0.844 |

The table shows results from univariate linear regression analyses (β and *P*-values) in three pairwise comparisons: COMBI-db/db vs. MET-db/db, COMBI-db/db vs. VG-db/db, and VG-db/db vs. WT. A *P*‑value < 0.05 was considered statistically significant and is shown in bold. **Abbreviations:** WT, wild type mice; VG-db/db, vehicle‑gavaged diabetic mice; MET-db/db, metformin-treated diabetic mice; COMBI-db/db, Sodium-glucose-cotransporter-2-inhibitor and metformin-treated diabetic mice.

## Table S11. Six significant cerebellum metabolites in COMBI vs. MET-db/db

|  | COMBI-db/db vs. MET-db/db | | COMBI-db/db vs. VG-db/db | | VG-db/db vs. WT | |
| --- | --- | --- | --- | --- | --- | --- |
| Metabolites | β | *P*-values | β | *P*-values | β | *P*-values |
| Arg | 0.95 | **0.029** | 0.61 | **0.176** | 0.58 | 0.201 |
| Ile | 1.22 | **3.18x10^-03^** | 1.39 | **4.16x10^-04^** | 1.48 | **1.01x10^-04^** |
| Pro | 0.89 | **0.042** | 0.34 | 0.465 | 1.32 | **1.02x10^-03^** |
| Tyr | -1.08 | **0.011** | -0.78 | 0.079 | 0.38 | 0.414 |
| Val | 1.16 | **5.50x10^-03^** | -0.01 | 0.982 | 1.09 | **0.011** |
| PC ae C40:3 | 0.88 | **0.045** | 0.68 | 0.129 | -0.41 | 0.373 |

The table shows results from univariate linear regression analyses (β and *P*-values) in three pairwise comparisons: COMBI-db/db vs. MET-db/db, COMBI-db/db vs. VG-db/db, and VG-db/db vs. WT. A *P*‑value < 0.05 was considered statistically significant and is shown in bold. **Abbreviations:** WT, wild type mice; VG-db/db, vehicle‑gavaged diabetic mice; MET-db/db, metformin-treated diabetic mice; COMBI-db/db, Sodium-glucose-cotransporter-2-inhibitor and metformin-treated diabetic mice.

## Table S12. Threonine metabolism-associated hepatic transcripts in COMBI-db/db mice compared with control groups

|  | COMBI-db/db vs. MET-db/db | | COMBI-db/db vs. VG-db/db | | VG-db/db vs. WT | |
| --- | --- | --- | --- | --- | --- | --- |
| Transcripts | Log_2_FC | *P*-values | Log_2_FC | *P*-values | Log_2_FC | *P*-values |
| *Slc38a2* | 1.05 | **4.08x10^-08^** | 1.02 | **4.27x10^-07^** | 0.02 | 0.87 |
| *Shmt2* | -0.15 | 0.15 | -0.05 | 0.57 | 0.28 | **4.54x10^-04^** |
| *Shmt1* | 0.06 | 0.38 | -0.12 | 0.21 | 0.38 | **1.59x10^-04^** |
| *Gldc* | 0.05 | 0.45 | -0.11 | 0.35 | 0.49 | **5.27x10^-06^** |

The table shows results from limma moderated t-tests with Surrogate variable analysis for adjusting batch effect (Log_2_ Fold Change [Log_2_FC] and *P*-values) in three pairwise comparisons: COMBI-db/db vs. MET-db/db, COMBI-db/db vs. VG-db/db, and VG-db/db vs. WT. A *P*‑value < 0.05 are shown in bold. **Abbreviations:** WT, wild type mice; VG-db/db, vehicle‑gavaged diabetic mice; MET-db/db, metformin-treated diabetic mice; COMBI-db/db, Sodium-glucose-cotransporter-2-inhibitor and metformin-treated diabetic mice. *Slc38a2*, [sodium-coupled neutral amino acid transporter 2](https://www.google.com/search?q=sodium-coupled+neutral+amino+acid+transporter+2&client=firefox-b-d&sca_esv=6fc980339ac54f7c&sxsrf=AE3TifNOE7LRoK5sTefiVUzp4YdhuDnvfA%3A1761999479882&ei=d_oFadHGNdmI7NYP04be-QM&ved=2ahUKEwjy_-eu39WQAxW7BdsEHW5yCcsQgK4QegQIARAB&uact=5&oq=Abbreviation+of+Slc38a2&gs_lp=Egxnd3Mtd2l6LXNlcnAiF0FiYnJldmlhdGlvbiBvZiBTbGMzOGEyMgUQIRigATIFECEYoAEyBRAhGKABMgUQIRigAUiPKVC_A1jtJHABeAGQAQCYAfkBoAGqGqoBBjAuMTEuNrgBA8gBAPgBAfgBApgCEqAC1hvCAgoQABiwAxjWBBhHwgILEAAYgAQYkQIYigXCAhAQLhiABBjRAxhDGMcBGIoFwgILEC4YgAQY0QMYxwHCAgUQABiABMICChAAGIAEGEMYigXCAg0QLhiABBhDGNQCGIoFwgIKEC4YgAQYQxiKBcICEhAAGIAEGEMYyQMYigUYRhj5AcICCxAAGIAEGJIDGIoFwgIFEC4YgATCAiwQABiABBhDGMkDGIoFGEYY-QEYlwUYjAUY3QQYRhj5ARj0Axj1Axj2A9gBAZgDAIgGAZAGCLoGBggBEAEYE5IHBTEuOS44oAeBxAGyBwUwLjkuOLgHxBvCBwwwLjIuMTIuMy4wLjHIB4UB&sclient=gws-wiz-serp&mstk=AUtExfCerJkNeuL0XDbihQ-3U0Y1Iw1qc2aF62Y185xS0b_eaPnfCDdtZ1Fba4gM5O0ZLd7FfeKLW7jWCnvRPfof4mzpiWmlVBLbP4YozjgbyCTT7ko3sQBKNRMvwVgXzl-cUnIZS6-QMn0y2_OpoMNSRmylRfkXY8eynhUI0bO3_2gpKag&csui=3); *Shmt2*, Serine Hydroxymethyltransferase 2; *Shmt1*, Serine Hydroxymethyltransferase 1; *Gldc*, glycine decarboxylase.

## Table S13. Group comparisons between COMBI-T2D (N = 25) and MET-T2D (N = 138): effect sizes, confidence intervals, and post-hoc power

| Metabolite | COMBI (n=25) Mean ± SD | MET-T2D (n=138) Mean ± SD | Cohen’s d (95% CI) | Power | MDE |
| --- | --- | --- | --- | --- | --- |
| C2 | 0.36 ± 1.11 | -0.07 ± 0.97 | -0.43 (-0.86 – 0.00) | 0.50 | 0.61 |
| Arg | -0.47 ± 0.98 | 0.08 ± 0.98 | 0.56 (0.13 – 0.99) | 0.73 | 0.61 |
| Ile | 0.53 ± 0.83 | -0.10 ± 1.00 | -0.64 (-1.07 – -0.20) | **0.83** | 0.61 |
| Leu | 0.58 ± 0.86 | -0.10 ± 0.99 | -0.70 (-1.13 – -0.26) | **0.89** | 0.61 |
| Thr | -0.41 ± 0.86 | 0.07 ± 1.01 | 0.49 (0.06 – 0.92) | 0.61 | 0.61 |
| Val | 0.52 ± 0.91 | -0.09 ± 0.99 | -0.62 (-1.06 – -0.19) | **0.81** | 0.61 |
| 3-IAA | 0.26 ± 1.07 | -0.05 ± 0.98 | -0.30 (-0.73 – 0.13) | 0.28 | 0.61 |
| DCA | 0.38 ± 0.61 | -0.07 ± 1.04 | -0.45 (-0.88 – -0.02) | 0.54 | 0.61 |
| HexCer (d16:1/22:0) | 0.46 ± 1.09 | -0.08 ± 0.97 | -0.54 (-0.98 – -0.11) | 0.70 | 0.61 |
| TG (20:4_36:4) | 0.53 ± 0.78 | -0.10 ± 1.01 | -0.64 (-1.07 – -0.20) | **0.83** | 0.61 |

Data are presented as mean ± standard deviation. Cohen’s d with 95% confidence interval (CI), post-hoc power (α = 0.05), and minimum detectable effect size (MDE, d at 80% power) are shown. For several branched-chain amino acids (Leu, Ile, Val) and TG (20:4/36:4), moderate-to-large effects were observed with sufficient power (≥ 0.8). For other metabolites, confidence intervals were wide and/or power was < 0.8, indicating exploratory findings.

## Table S14. Sensitivity analyses of the 10 metabolites in six models

|  | S-model 1 | | S-model 2 | | S-model 3 | | S-model 4 | | S-model 5 | | S-model 6 | |
| --- | --- | --- | --- | --- | --- | --- | --- | --- | --- | --- | --- | --- |
| Metabolites | β | *P* | β | *P* | β | *P* | β | *P* | β | *P* | β | *P* |
| C2 | 0.47 | **0.031** | 0.51 | **0.019** | 0.45 | **0.034** | 0.45 | **0.040** | 0.40 | 0.06 | 0.44 | **0.042** |
| Arg | -0.54 | **0.013** | -0.58 | **6.54x10^-3^** | -0.58 | **6.50x10^-3^** | -0.62 | **4.91x10^-3^** | -0.57 | **7.68x10^-3^** | -0.57 | **6.13x10^-3^** |
| Ile | 0.73 | **3.56x10^-4^** | 0.75 | **2.73x10^-4^** | 0.75 | **2.92x10^-4^** | 0.63 | **1.94x10^-4^** | 0.63 | **1.88x10^-3^** | 0.82 | **4.79x10^-5^** |
| Leu | 0.77 | **1.38x10^-4^** | 0.77 | **1.54x10^-4^** | 0.77 | **1.67x10^-4^** | 0.67 | **1.08x10^-3^** | 0.67 | **1.02x10^-3^** | 0.84 | **4.05x10^-5^** |
| Thr | -0.44 | **0.039** | -0.45 | **0.032** | -0.47 | **0.028** | -0.46 | **0.034** | -0.44 | **0.048** | -0.50 | **0.018** |
| Val | 0.67 | **1.65x10^-3^** | 0.66 | **1.98x10^-3^** | 0.64 | **2.66x10^-3^** | 0.55 | **0.012** | 0.58 | **7.64x10^-3^** | 0.72 | **7.03x10^-4^** |
| 3-IAA | 0.38 | 0.08 | 0.42 | 0.06 | 0.39 | 0.07 | 0.41 | 0.07 | 0.48 | **0.030** | 0.43 | **0.049** |
| DCA | 0.49 | **0.025** | 0.49 | **0.027** | 0.51 | **0.023** | 0.45 | **0.048** | 0.47 | **0.044** | 0.53 | **0.019** |
| HexCer(d16:1/22:0) | 0.45 | **0.030** | 0.44 | **0.034** | 0.48 | **0.021** | 0.44 | **0.042** | 0.44 | **0.046** | 0.45 | **0.031** |
| TG(20:4_36:4) | 0.55 | **9.77x10^-3^** | 0.54 | **0.012** | 0.54 | **0.012** | 0.47 | **0.037** | 0.44 | 0.05 | 0.44 | **0.038** |

The table shows results from multivariable linear regression analyses (β and *P*) adjusted for six sensitivity models between COMBI-T2D and MET-T2D groups. A *P* < 0.05 is shown in bold.

S-model 1: metabolite ~ COMBI/MET + age + sex

S-model 2: S-model 1 + smoking + Physical activity

S-model 3: S-model 2 + BMI

S-model 4: S-model 3 + HDL cholesterol + Fasting glucose

S-model 5: S-model 4+ alcohol consumption + hypertension

S-model 6: metabolite ~ COMBI/MET + age + sex + BMI + smoking + Physical activity + HDL cholesterol + alcohol consumption

## Table S15. Correlation between HbA_1C_ and 10 metabolites in T2D patients treated with COMBI or MET

| Metabolite | *r* | *P*-value |
| --- | --- | --- |
| C2 | -0.08 | 0.284 |
| Arg | -0.18 | **0.023** |
| Ile | 0.19 | **0.013** |
| Leu | 0.16 | **0.043** |
| Thr | -0.18 | **0.024** |
| Val | 0.17 | **0.032** |
| 3-IAA | -0.03 | 0.665 |
| DCA | 0.13 | 0.103 |
| HexCer (d16:1/22:0) | 0.13 | 0.096 |
| TG (20:4_36:4) | 0.22 | **0.005** |

This table presents Spearman’s correlation coefficients (*r*) and *P*‑values between HbA_1C_ and the concentrations of the 10 metabolites in patients treated with COMBI therapy (N = 25) or metformin monotherapy (N = 138).

## Figure S1. Comparisons of the liver transcripts between COMBI-db/db and MET-db/db


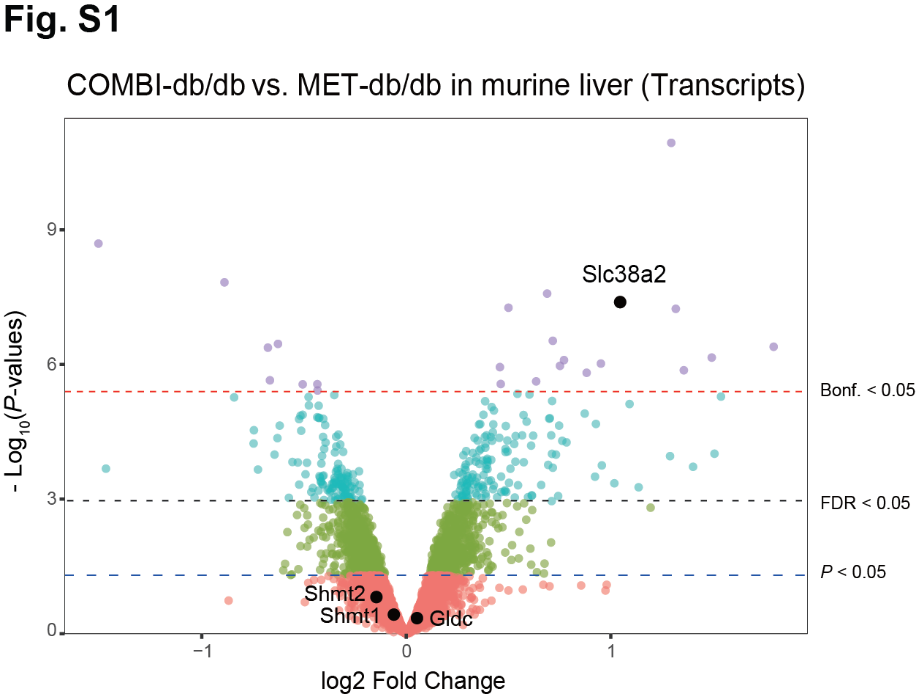


Hepatic expression of Slc38a2 (also known as Snat2), a member of the SLC38 system, a family of neutral amino acid transporters, was significantly higher in the COMBI group compared with metformin monotherapy. In contrast, we did not observe significant differences in transcripts related to threonine metabolism (such as Gldc, Shmt1, and Shmt2), suggesting that the paradoxical distribution pattern of threonine is unlikely to be explained by altered catabolism.

## Figure S2. Sensitivity analyses using propensity score matching (PSM)


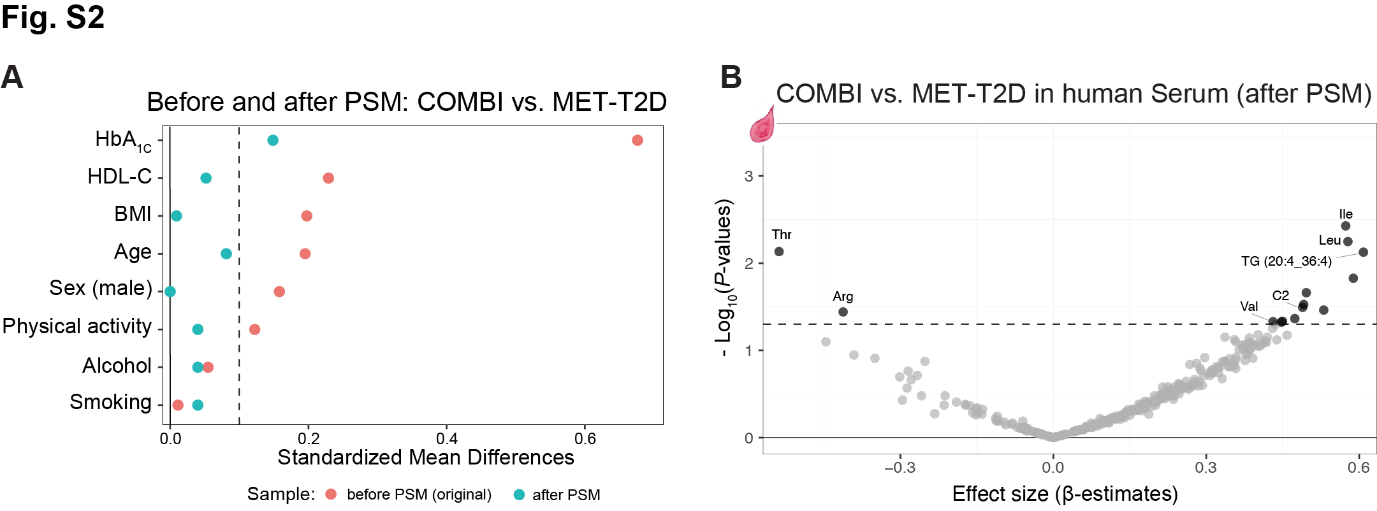


A. Standardized mean differences (SMD) for clinical covariates in the COMBI-T2D vs. MET-T2D comparison before and after PSM using (1:2 ratio, COMBI:MET). After matching, HbA_1C_ slightly higher in the COMBI group (SMD < 0.2, while all other covariates achieved good balance (SMD < 0.1).

B. showing metabolite associations between propensity‑score–matched COMBI‑T2D and MET‑T2D groups based on multivariable regression using the primary adjustment model. Seven metabolites (Thr, Arg, Val, Ile, Leu, C2, and TG(20:4_36:4)) remained significantly associated with COMBI therapy.
